# Risk-based antihypertensive treatment allocation in Peru: comparison of local and international guidelines analysing national health surveys between 2015-2020

**DOI:** 10.1016/j.lana.2021.100022

**Published:** 2021-07-26

**Authors:** Rodrigo M. Carrillo-Larco, Wilmer Cristobal Guzman-Vilca, Antonio Bernabe-Ortiz

**Affiliations:** 1 Department of Epidemiology and Biostatistics, School of Public Health, Imperial College London, London, UK.; 2 CRONICAS Centre of Excellence in Chronic Diseases, Universidad Peruana Cayetano Heredia, Lima, Peru; 3 School of Medicine “Alberto Hurtado”, Universidad Peruana Cayetano Heredia, Lima, Peru; 4 Sociedad Científica de Estudiantes de Medicina Cayetano Heredia (SOCEMCH), Universidad Peruana Cayetano Heredia, Lima, Peru; 5 Universidad Científica del Sur, Lima, Peru

**Keywords:** cardiovascular risk, cardiovascular diseases, health metrics

## Abstract

**Background:**

While there is a growing interest in antihypertensive medication rates among people with hypertension in low- and middle-income countries, little has been described about antihypertensive medication rates among eligible people based on the absolute cardiovascular risk approach. Following the risk-based approach, we described the proportion of eligible people receiving antihypertensive medication in Peru.

**Methods:**

Analysis of six (2015-2020) national health surveys. Absolute cardiovascular risk was computed with the 2019 WHO cardiovascular risk charts and based on local guidelines. Antihypertensive treatment allocation based on the absolute cardiovascular risk was defined using the Package of essential noncommunicable (PEN) disease interventions for primary health care in low-resource settings and the HEARTS guidelines by the WHO; we also followed the recommendations by local guidelines.

**Results:**

There were 120,059 people. Overall, according to the local guidelines the 17.9% of the population would be eligible for antihypertensive medication while this estimate was 8.1% based on the WHO guidelines. At the national level, depending on the guidelines, we observed a steady trend of eligible people receiving antihypertension medication (e.g., men, local guidelines), a decreasing trend (e.g., men, <60, local guidelines) or an increasing trend (e.g., men, ≥60, local guidelines). At the sub-national level, seventeen regions showed an increasing antihypertensive treatment rate based on the local guidelines; when based on the WHO guidelines, eleven regions showed a decreasing rate.

**Conclusions:**

Peru needs to define a tool for surveillance of absolute cardiovascular risk and to monitor antihypertensive treatment allocation among high-risk people.

**Funding:**

Wellcome Trust (214185/Z/18/Z).

**Research in context**

**Evidence before this study**

We searched PubMed on June 5th, 2021 without restrictions. The search was: ("Peru" OR "Latin America" OR "South America") AND ("antihypertensive treatment" OR "hypertensive treatment") AND ("risk-based approach"). This search did not retrieve any results, which suggests that the absolute cardiovascular risk approach to identify people eligible for antihypertensive treatment has been poorly studied in Latin America. Most of the evidence about antihypertensive treatment rates have focused on the hypertension treatment cascade: among people with hypertension, how many were aware of their condition, how many were receiving treatment and how many have reached blood pressure control. Recently, a global work using STEPS surveys followed the absolute cardiovascular risk approach to study antihypertensive treatment eligibility. Although this work opened the route to study antihypertensive treatment rates among eligible people based on their absolute cardiovascular risk, they did not include Peru, did not study time trends and did not provide sub-national results.

**Added value of this study**

We analysed six national health surveys in Peru. We defined eligibility for antihypertensive medication based on the absolute cardiovascular risk following guidelines by the World Health Organization (WHO) and those proposed by local authorities. We computed the proportion of people eligible for antihypertensive treatment, and how many of these were receiving this medication. We informed about the proportion of the population not eligible for antihypertensive treatment who were receiving this medication. Our results are at the national and sub-national levels, also by sex and age.

**Implications of all available evidence**

The preliminary literature search suggested that in Latin America, there are limited evidence about the allocation of antihypertensive medication based on the risk-based approach. Our work complemented other global efforts focusing on one country in Latin America, delivering results for six years and at the sub-national level. The results are uniquely positioned to spark the interest in the risk-based approach to assess antihypertensive treatment allocation in Latin America. Furthermore, our results have the potential to inform the monitoring of antihypertensive treatment rates in Peru according to the WHO guidelines and indicators. Finally, our results invite authorities and practitioners in Peru to revise how antihypertensive treatment rates are monitored and where treatment rates remain poor.

Alt-text: Unlabelled box

## 1 Introduction

From a clinical perspective, someone with high blood pressure (e.g., ≥160/100 mmHg) would undoubtedly need antihypertensive medication regardless of their absolute cardiovascular risk [1], [2], [3], [4], [5] however, the decision to initiate such medication when the blood pressure is not high (e.g., 130-139/80-89 mmHg) may require additional variables to take an appropriate decision. This is when the absolute cardiovascular risk plays a relevant role in informing antihypertensive treatment prescription. [1], [2], [3], [4], [5], [6], [7], [8]

Antihypertensive treatment rates have been usually assessed as the proportion of people with hypertension receiving antihypertensive medication, and this has been often studied in the context of the hypertension treatment cascade. [9], [10], [11], [12], [13] However, this rate could hide people with mild high blood pressure and low absolute cardiovascular risk who may not necessarily need antihypertensive medication. [7],[8] Therefore, it is relevant to revisit the prevalence of self-reported antihypertensive medication among eligible subjects according to the risk-based approach. A recent global work has delivered this evidence for forty-five low- and middle-income countries. [14] Nonetheless, some low- and middle-income countries were not included, time trends were not studied, and national estimates may hide sub-national inequalities which need to be addressed by local authorities with the support of global partners (e.g., the World Health Organization (WHO)).

We aimed to describe the rates of antihypertensive medication among eligible and non-eligible subjects based on the absolute cardiovascular risk approach. The eligibility criteria were based on high cardiovascular risk according to the ten-year 2019 WHO cardiovascular risk charts and the local official guidelines in Peru. [2],[15] We used six (2015-2020) national health surveys and delivered estimates at the national and sub-national levels, stratified by sex and age.

## 2 Methods

### Data sources

This is an analysis of six consecutive national health surveys in Peru. We analysed the Demographic and Health Survey (DHS or ENDES for its name in Spanish) between 2015 and 2020. During the study period, the ENDES included a national and regional representative sample of men and women. The ENDES included a health questionnaire (e.g., self-reported diagnosis) and measurements of weight, height, waist circumference and two blood pressure measurements. The ENDES follows standard methods as in other DHS surveys. The ENDES follows a probability sampling approach. This is done utilizing two-stage cluster sampling, where the clusters are defined according to rural and urban settings. The two-stage cluster sampling involves selection of a limited number of villages in each stratum. In a second step, a limited and fixed number of households within each cluster is selected.

Even though the WHO HEARTS Technical Package for Cardiovascular Disease Management in Primary Health Care suggests that our outcome of interest should be assessed every five years, [6] in here we used all six national surveys to also study geographic and time trends.

### Study population

We studied men and women aged between 30 and 80 years (inclusive). Although the 2019 ten-year WHO cardiovascular risk charts were developed for people aged 40-80, [15] consistent with recommendations by the Package of essential noncommunicable (PEN) disease interventions for primary health care in low-resource settings, [16] for people younger than 40 years we assumed they were 40 years old for the absolute cardiovascular risk estimation as done in a recent global work. [14]

### Variables

#### Original variables

The ENDES survey applies a standard protocol and questionnaire from which we extracted: sex, age and region (there are 25 regions in Peru). These variables were used to stratify the main outcomes.

We defined smoker based on one question which was coded as no versus yes: *do you smoke every day?* Self-reported information about antihypertensive treatment was gathered from one question also coded as no (including “does not know”) versus yes (Supplementary Table 1): *in the last twelve months, have you received and/or bought medicines to control your blood pressure?* Information was also available for self-reported diabetes diagnosis: *have you ever been diagnosed by a physician with diabetes or high blood sugar?* This variable was coded as no (including “does not know”) versus yes (Supplementary Table 1).

The ENDES survey also collects anthropometrics and two blood pressure measurements; these are taken by trained fieldworkers following a standard protocol. Measured weight (kg) and height (m) were used to compute the body mass index (BMI). BMI records outside the range 10-80 kg/m^2^ were discarded. We only used the second blood pressure measurement. Systolic blood pressure records outside the range 70-270 mmHg were discarded, and so were diastolic blood pressure records outside the 30-150 mmHg range (Supplementary Table 2).

Consistent with the PEN protocol and with a previous global work, [14],[16] for people with self-reported antihypertensive treatment we used the pre-treatment blood pressure. In other words, for people who self-reported being under antihypertensive treatment, we calculated the pre-treatment blood pressure; conversely, for those who were not taking antihypertensive medicines, we used the recorded blood pressure as was. Pre-treatment systolic blood pressure was computed as: pre-treatment systolic blood pressure = (current systolic blood pressure-6.3)/0.9. [17] Similarly, pre-treatment diastolic blood pressure was computed as: pre-treatment diastolic blood pressure = (current diastolic blood pressure-5.17)/0.89. [17]

#### Derived variables

First, we computed the absolute cardiovascular risk based on the 2019 ten-year WHO cardiovascular risk charts. [15] Because we did not have information on total cholesterol we used the office-based WHO cardiovascular risk charts. [15] The office-based ten-year WHO cardiovascular risk charts include age, sex, smoking status, systolic blood pressure (pre-treatment) and BMI. We used the *whocvdrisk c*ommand in STATA by the authors of the 2019 ten-year WHO cardiovascular risk charts. [18] Based on the WHO HEARTS protocol for risk-based cardiovascular prevention, [5] we considered the following people as eligible for antihypertensive treatment: i) absolute cardiovascular risk between 10%-19% and blood pressure ≥140 (systolic) or ≥90 (diastolic) mmHg; ii) absolute cardiovascular risk ≥20% and blood pressure ≥130 or ≥80 mmHg; and iii) all individuals with blood pressure ≥160 or ≥100 mmHg. [5] Observations which did not fell into any of these groups were not considered eligible for antihypertensive treatment.

Second, we followed the local hypertension diagnostic, treatment and control guidelines issued in 2015 by the Peruvian Ministry of Health. [2] This document defines hypertension as ≥140 or 90 mmHg and suggests to initiate antihypertensive treatment in: i) people with hypertension and low cardiovascular risk who have failed to achieve blood pressure targets after 3-6 months of lifestyle changes; ii) blood pressure ≥160 (systolic) or ≥100 (diastolic) regardless of other variables; and iii) people with hypertension with moderate or high cardiovascular risk. We used the second and third criteria to define people eligible for antihypertensive treatment because we did not have information on whether the participants had tried lifestyle changes for 3-6 months. Observations which did not fell into any of those two groups were not considered eligible for antihypertensive treatment. The Peruvian guidelines defined cardiovascular risk based on the presence of risk factors. [19] According to the available data we used five risk factors: [2],[20] i) overweight (BMI ≥25 kg/m^2^); ii) male sex; iii) men aged ≥55 or women aged ≥65; iv) smoker; and v) self-reported diabetes.

#### Outcomes

The fifth indicator in the monitoring plan of the WHO HEARTS Technical Package for Cardiovascular Disease Management in Primary Health Care refers to the *proportion of eligible persons receiving drug therapy and counselling to prevent heart attacks and strokes.* *^6^* The numerator refers to the number of eligible people receiving drug therapy and counselling. [6] The denominator refers to the total number of eligible participants. [6] In this study, the numerator was the self-reported antihypertensive treatment variable, whereas the denominator was the eligible or non-eligible population according to the 2019 ten-year WHO cardiovascular risk charts or the Peruvian guidelines (defined in the previous section). [2],[15] The proportion of people receiving antihypertensive treatment among those who were eligible or non-eligible for treatment was summarized at the national and regional level, and also stratified by age (<60 and ≥60) and sex when possible.

### Analysis

The datasets we used and the analysis code in R (version 3.6.1) and STATA (version 16.1, College Station, Texas 77845, USA) are available as supplementary materials. We summarized the outcome of interest along with the 95% confidence interval (95% CI) accounting for the complex survey design of the ENDES. The outcome was summarised at the national level, stratified by sex and age group (<60 and ≥60). The results were also summarised at the macro-region level; that is, the 25 regions in Peru were grouped according to their geographic location: Coast, Highlands and Amazon. The results were summarised for each of the 25 regions in Peru as well. The absolute cardiovascular risk computed from the 2019 ten-year WHO cardiovascular risk charts was categorised and the proportion in each stratum multiplied by the number of people in Peru aged ≥30 years according to the 2017 national census (15,098,012 people). [21]

### Ethics

We did not seek approval by an Institutional Review Board. This was an analysis of open-access surveys which do not include any personal identifiers.

### Role of the funding source

The funder of the study had no role in study design, data collection, data analysis, data interpretation, or writing of the report. RMC-L and WCG-V had full access to the data in the study and vouch for the data accuracy. All authors collectively had final responsibility for the decision to submit for publication. The authors alone are responsible for the opinions in the manuscript, which do not necessarily represent those of their institutions.

## 3 Results

### Study population

In total, there were 120,059 people and each year contributed equally to the pooled analysis database (except the 2020 survey which represented 12% of the pooled dataset). Overall, there were discretely more women (51.2%) and the mean age was 48.6 years. The mean systolic blood pressure was 123.1 mmHg, and the mean pre-treatment systolic blood pressure was 123.8 mmHg. The mean BMI was 27.8 kg.m^2^, and 2.0% of the population were smokers.

### Absolute cardiovascular risk according to the 2019 WHO cardiovascular risk charts

Overall, the mean absolute cardiovascular risk based on the 2019 ten-year WHO cardiovascular risk charts was 3.6% (95% CI: 3.5%-3.6%). There were no substantial differences between study years, where the minimum absolute risk was 3.5% (95% CI: 3.4%-3.6%) in 2015 and the maximum absolute risk was 3.7% (95% CI: 3.6%-3.7%) in 2018 (Supplementary Table 3). The absolute cardiovascular risk was higher in men (4.1% (95% CI: 4.1%-4.2%)) than in women (3.1% (95% CI: 3.0%-3.1%)). The absolute cardiovascular risk was higher among people aged ≥60 years (9.0 (95% CI: 8.9%-9.1%)) than in their younger peers (2.0% (95% CI: 2.0%-2.0%)).

In general, 78.6% (95% CI: 78.2%-79.0%) of the population had an absolute cardiovascular risk <5%; which is equivalent to 11,867,037 people (i.e., 78% of total population in Peru aged ≥30 years). [21] The proportion of the population with an absolute cardiovascular risk between 5%-9% was 13.8% (95% CI: 13.5%-14.1%); equivalent to 2,083,526 people. The frequency of absolute cardiovascular risk between 10%-19% was 7.0% (95% CI: 6.7%-7.2%) or 1,056,860 people. The 0.6% (95% CI: 0.5%-0.7%) of the population had an absolute risk of 20%-29%; equivalent to 90,588 people. Finally, 0.04% of the population had an absolute risk of ≥30%; equivalent to 6,039 people.

**Figure 1:**
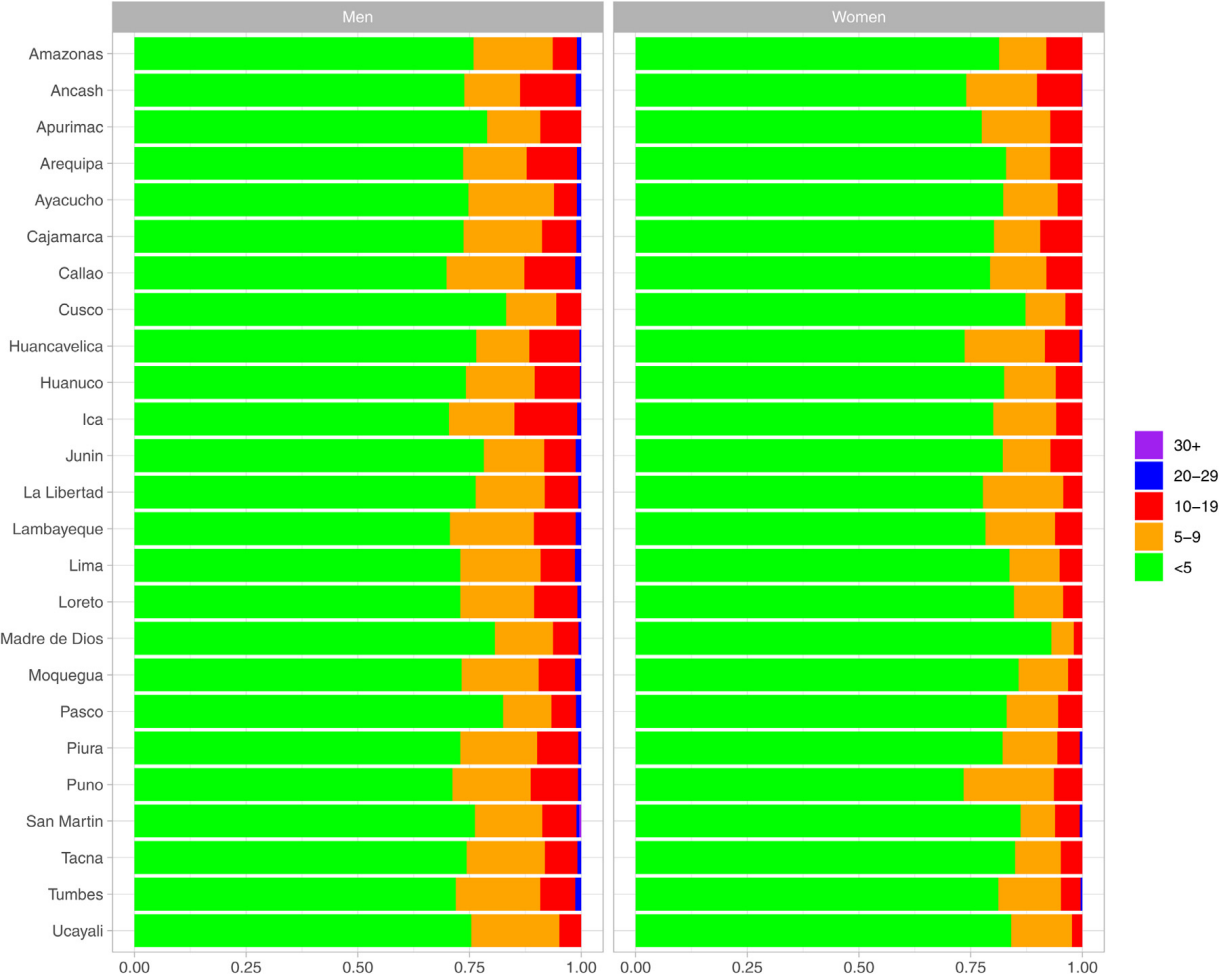
Absolute cardiovascular risk in categories by sex and region in 2019. Exact numeric estimates, and results for all study years, are available in Supplementary Table 5.

In 2020, the largest proportion of men with an absolute cardiovascular risk <5% was observed in Cusco (83.2%), and the smallest in Callao (69.9%); for women these numbers were 93.2% (Madre de Dios) and 73.5% (Puno) (Figure 1; Supplementary Table 5).

### Eligible population for antihypertensive treatment

According to the local guidelines and across the observation period, the 17.9% (95% CI: 17.5%-18.3%) of the population would be eligible for antihypertensive treatment (Supplementary Table 4). Conversely, according to the WHO guidelines and across the study period, the 8.1% (95% CI: 7.9%-8.4%) of the population would be eligible for antihypertensive treatment (Supplementary Table 4).

### National results of eligible people receiving antihypertensive treatment

**Figure 2:**
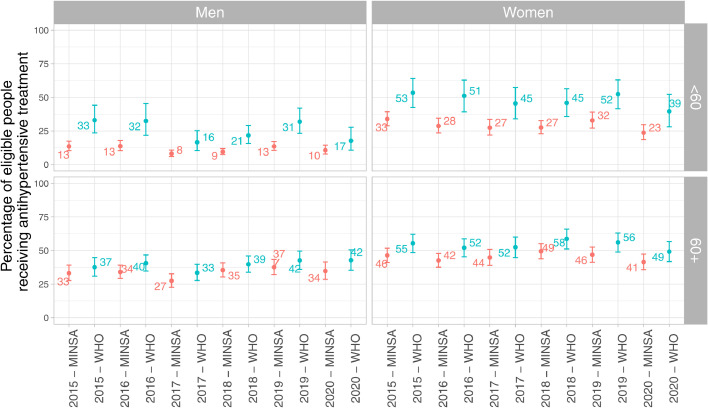
Percentage of people receiving antihypertensive medication among eligible subjects by sex and age at the national level. Exact numeric estimates are available in Supplementary Table 6.

In men, and according to the WHO guidelines, we observed a U-shaped time trend whereby the largest proportion of eligible people receiving antihypertensive treatment was seen in 2015 (2016 for men aged <60 years) and in 2019, though the smallest proportion was found in 2017 yet also in 2020 (Figure 2; Supplementary Table 6). The same profile was observed according to the local guidelines, yet with a smaller magnitude (Figure 2; Supplementary Table 6). On the other hand, the proportion of not eligible men aged <60 years receiving antihypertensive medication has remained stable regardless of the guidelines; in men aged ≥60 years the proportion of not eligible men receiving antihypertensive medication has increased by 4 (WHO guidelines) and 5 (local guidelines) percentage points between 2015 and 2019; a smaller proportion was registered in 2020 (Supplementary Figure 1; Supplementary Table 6).

In women aged <60 years and regardless of the selected guidelines, a U-shaped time trend was also observed; in 2020 we observed smallest proportions (Figure 2; Supplementary Table 6). In women aged ≥60 according to the WHO guidelines there was a marginal increase in the proportion of eligible women receiving antihypertensive medication: 55% in 2015 and 56% in 2019; the smallest proportions were observed in 2020 (Figure 2; Supplementary Table 6). On the other hand, the proportion of not eligible women aged <60 years receiving antihypertensive medication remained stable; the proportion of not eligible women aged ≥60 years receiving antihypertensive medication marginally increased by 4 (WHO guidelines) and 5 (local guidelines) percentage points (Supplementary Figure 1; Supplementary Table 6).

### Sub-national results of eligible people receiving antihypertensive treatment

At the macro-region level in men and regardless of the guidelines, we observed a U-shaped curve in the Coast whereby the proportion of eligible men receiving antihypertensive treatment improved in 2018 and 2019, yet there was a marginal decrease in 2020 (Supplementary Figure 1). In men in the Amazon and regardless of the guidelines, we observed a decreasing trend since 2016 (Supplementary Figure 1). In the Highlands there was a discrete increase since 2015 (Supplementary Figure 1).

At the macro-region level in women and regardless of the guidelines, in the Coast we observed a decreasing trend (Supplementary Figure 1). In women in the Amazon there was a U-shaped curve based on the WHO guidelines and an increasing trend based on the local guidelines; regardless of the guidelines, smaller proportions were found in 2020 (Supplementary Figure 1). In the Highlands there was an increasing trend until 2019, and smaller proportions in 2020 (Supplementary Figure 1).

No region showed a substantial improvement in allocation of antihypertensive treatment among eligible subjects (Supplementary Figure 4). Similarly, no region showed a large improvement in reducing antihypertensive mediation among not eligible people (Supplementary Figure 5). It was not possible to further stratify these sub-national estimates by sex and age because of few observations in some groups which led to unstable estimates.

Following the local guidelines between 2015 and 2019, we observed that seventeen regions slightly increased the antihypertensive treatment rate among eligible subjects; we observed a marginal decrease in seven regions (Supplementary Figure 4; Supplementary Table 7). In eight regions in 2020 we observed the smallest proportions (Supplementary Figure 4; Supplementary Table 7). Conversely, the variation of antihypertensive treatment allocation among not eligible people was minimal during the study period (Supplementary Figure 5; Supplementary Table 7).

Following the WHO guidelines between 2015 and 2019, the antihypertensive treatment rate among eligible individuals increased in twelve regions; in eleven regions this treatment rate decreased (Supplementary Figure 4; Supplementary Table 7). In seven regions in 2020 we observed the smallest proportions (Supplementary Figure 4; Supplementary Table 7). The variation of antihypertensive treatment allocation among not eligible people was minimal across the study period (Supplementary Figure 5; Supplementary Table 5).

## Discussion

### Main findings

Elaborating on six national health surveys in Peru, the mean absolute cardiovascular risk based on the 2019 ten-year WHO cardiovascular risk charts was 3.5%-3.7% with higher absolute risk in people aged ≥60 years. Over three quarters of the population have an absolute cardiovascular risk <5%, and less than one in a hundred people had an absolute cardiovascular risk ≥20%. The proportion of people eligible for antihypertensive treatment was higher with the local guidelines than with the WHO guidelines: a 2-fold difference. The proportion of eligible people receiving antihypertensive medication would consistently be higher with the WHO guidelines than with the local guidelines. At the national level in men of any age and regardless of the guidelines, the proportion of eligible men receiving antihypertensive treatment has slightly improved in the last years with a marginal decrease in 2020. In women aged ≥60 according to the WHO guidelines the proportion of eligible women receiving antihypertensive treatment has marginally decreased. Also, at the national level, the proportion of not eligible people aged ≥60 years receiving antihypertensive medication marginally increased in both men and women and regardless of the guidelines. At the sub-national level, we observed a heterogeneous profile. Following the local guidelines there were seventeen regions with a discrete increase in the proportion of eligible people receiving treatment; conversely, following the WHO guidelines there were sixteen regions with a marginal decrease in the proportion of eligible people receiving antihypertensive treatment. The variation in the proportion of not eligible people receiving antihypertensive medication was minimal. Together with the available evidence of antihypertensive treatment rate among people with hypertension, both local and global, our results suggest that antihypertensive treatment coverage needs to improve in Peru (as it the case in other low- and middle-income countries).

Peru has not adopted the WHO cardiovascular guidelines for risk-based primary cardiovascular prevention; to the best of our knowledge, Peru is not planning to adopt these guidelines soon. This work was not designed to support or advocate for one approach over the other (e.g., whether local guidelines should be replaced by the WHO guidelines). This work was a description of the current antihypertension treatment coverage according to two definitions. This comparison is relevant to advance and move beyond the usual hypertension care cascade (i.e., proportion of people with hypertension receiving antihypertensive medication); furthermore, these estimates could inform discussions about the antihypertensive treatment rate by advancing the available evidence with a new analytical approach (i.e., based on the absolute cardiovascular risk). If Peru decides to revisit its guidelines for primary cardiovascular prevention and incorporate the absolute risk approach, our estimates could anticipate what the treatment coverage may look like, so that policymakers can plan accordingly and set realistic targets. A further elaboration on the reasons why Peru has opted for one approach and has not decided to change that strategy (yet), is beyond the scope of this work.

### Interpretation of results

A global work with forty-five low- and middle-income countries using the office-based 2019 WHO cardiovascular risk charts showed that the median ten-year absolute cardiovascular risk was 2.7% in men and 1.6% in women; [14] the absolute ten-year cardiovascular risk in Latin America ranged from 1.2% (women in Costa Rica/Ecuador) to 4.4% (men in Chile). [14] The GLOBORISK [22] office-based model for fatal and non-fatal cardiovascular outcomes in Mexico showed that the mean ten-year cardiovascular risk was 6.6% in men and 4.2% in women. [23] Overall, our estimates are similar yet our results for women are slightly higher than those reported in the global work. [14] Our study also advanced this evidence by studying two age groups and finding higher absolute risk in older people. This last observation has two implications. First, older people need primary and secondary care to reduce their absolute cardiovascular risk. Second, primary prevention interventions should not wait until they are 60 years old, they should start in adulthood to avoid people reaching the age of 60 with high cardiovascular risk.

The proportion of people receiving antihypertensive medication among those eligible was higher when using the WHO guidelines versus the local guidelines. In other words, the health system performance seems better when based on the WHO guidelines. This could be owed to the different methodology to assess cardiovascular risk. The 2019 ten-year WHO cardiovascular risk charts adopted an absolute risk approach whereby risk factors are combined in a formula, and each risk factor has its own coefficient (i.e., weights). Conversely, the local guidelines suggested to count the number of risk factors and to combine this with blood pressure levels. This approach would assume each risk factor weights the same and there are no variations within risk factor levels; for example, with the local guidelines anyone with overweight would be assigned one point (i.e., presence of a risk factor) regardless of whether his/her BMI was 25.1 kg/m^2^ or 29.9 kg/m^2^. In addition to this methodological difference, we must acknowledge that differences between the local and international guidelines could be explained by the fact that we used the office-based model of the 2019 ten-year WHO cardiovascular risk charts and not the laboratory-based model. [15] The laboratory-based model could have identified more eligible subjects, diluting the proportion of eligible subjects receiving antihypertensive medication, thus reducing the gap between the local and international guidelines regarding treatment allocation. Our results, although relevant because they provide the first estimates of antihypertensive treatment allocation in Peru following a risk-based approach, deserve further verification using the laboratory-based model before public health interventions and policies are implemented based on these findings.

### Implications for clinical management

The absolute cardiovascular risk-based prevention approach, endorsed by the WHO, [5],[6] is also suggested by several clinical guidelines. [1],[3],[4],[6] Moreover, the use of cardiovascular risk scores may reduce risk factor levels and may increase preventive medication allocation among high-risk people. [24] Possibly, the absolute risk-based approach would be better than the one currently suggested in Peru for clinical management of people with hypertension, and could therefore be adopted by the next version of the local recommendations following the example from several global guidelines. [1],[3],[4],[6] This hypothesis warrants solid research, including cost-effectiveness analyses, to make informed changes in local guidelines.

There was a small increase in the proportion of not eligible people aged ≥60 years receiving antihypertensive medication (particularly in women). From a clinical management perspective, careful consideration should be warranted when starting pharmacological treatment in the elderly, amongst whom polypharmacy is frequent. This decision should be made based on all available evidence, both theorical (e.g., guidelines) and clinical (e.g., unique profile of the patient), and in consultation with the patient's preferences (i.e., shared decision). Furthermore, multidisciplinary care including all relevant specialists (e.g., diabetologist and nephrologist) would be ideal.

### Implications for epidemiological research

Public health experts, [8] epidemiologists and cardiologists could argue that the lack of a local cardiovascular risk score or chart would rest value to instituting the absolute risk-based approach in Peru. This has pragmatic implications for health research in Peru and other similar countries. Prospective studies are warranted to validate, re-calibrate or develop a Peruvian cardiovascular risk score. In this line, (electronic) health records, surveillance and summary data could be used as a preliminary step. There are some successful experiences in Colombia and Chile, [25],[26] and soon will there be a cardiovascular risk score for Latin America [27] to overcome the limitations of other studies in Latin America which aimed to validate cardiovascular risk scores. [28] Until then, global efforts could be adopted including the 2019 WHO cardiovascular risk charts and the GLOBORISK. [15],[22]

### Implications for public health

We are not aware of any efforts to quantify the risk-based antihypertensive treatment allocation among eligible people according to local and international guidelines in Peru; thus, we do not have empirical evidence to contrast our results. Our work, interpreted along with its limitations, is uniquely positioned to inform public health interventions to improve the epidemiology of hypertension in Peru.

Our results inform about the antihypertensive treatment allocation following the absolute risk-based approach advancing the evidence from the hypertension treatment cascade. [13] Results from the hypertension treatment cascade in 2018 showed that ~23% of men and ~44% of women with hypertension were receiving antihypertensive drugs in Peru. [13] Our results are not far apart from these estimates. Antihypertensive drugs are in the WHO list of essential medicines, yet often these are not available or affordable. [29] The public health system should identify the barriers to have antihypertensive medication in all public pharmacies, and work with local authorities to secure treatment availability to all patients. If the risk-based approach were to be followed, patients would need access to adequate laboratory facilities for a full absolute cardiovascular risk assessment. Novel interventions that could be tested include self-monitoring blood pressure, which appears to improve antihypertensive medication adherence. [30]

Even though antihypertensive drugs are considered essential and should be available to all patients with hypertension, another problem is adherence which becomes more problematic when multiple drugs are prescribed. The polypill approach could be a solution, and this has been recommended by the Latin American Consensus in Hypertension. [31] Future cost-effectiveness studies could assess whether polypills would render better health effects to the patients along with more efficient allocation and purchases from the health system perspective.

Our results pinpoint population groups (e.g., older women and some regions) in which interventions may be needed to improve antihypertensive treatment allocation amongst eligible people. In this line, the sub-national results showed large inequalities amongst regions; this urgently calls for joint efforts between national and regional health authorities. For example, they could secure that there are cardiologists and other health professionals with proper training to manage hypertension in all regions. They could also work to plan the purchase and distribution of antihypertensive drugs so that these are never unavailable in public health facilities.

Our results could spark interest and advocacy for the revision of local guidelines to consider the absolute risk-based approach. The latest version of the local guidelines was published in 2015. Arguably, it is about time for them to be revisited in a joint effort including health authorities, practitioners, researchers and also patients. Our work, international clinical guidelines, [1],[3], [4], [5], [6] and novel tools for cardiovascular risk stratification could be discussed and incorporated in local guidelines.

Finally, we observed that the proportion of eligible subjects receiving antihypertensive medication slightly decreased in 2020 in relation to the previous years. The COVID-19 pandemic affected the Peruvian health system above and beyond the limited resources available for COVID-19 patients. Although speculative, we hypothesize that the eligible patients did not have frequent contact with their healthcare provider limiting their access to medications. This lack of contact could be because primary care facilities were closed, hospitals prioritized care for COVID-19 patients, local restrictions (e.g., quarantines), or people were afraid of going to healthcare facilities where there were COVID-19 patients. While Peru improves their capacity to look after COVID-19 patients, other programs (e.g., non-communicable diseases) need to catch up with their goals and services.

### Strengths and limitations

We studied six national health surveys with measured risk factors, and these are representative at the national and regional level; in addition, we included most of the adult population (i.e., ≥30 years). Notwithstanding, there are limitations we acknowledge. First, the ENDES survey measures blood pressure twice, not three times like an ideal epidemiological study. In here, we only used the second measurement. We would expect this approach to give slightly higher blood pressure records than those from an ideal epidemiological study. If so, then the absolute cardiovascular risk could be minimally overestimated. However, because we presented aggregated summaries (e.g., at the national level), we would not expect this to have substantially biased our results. Second, the ENDES does not ask about history of cardiovascular events and we therefore assumed all participants were free of cardiovascular diseases to use the 2019 WHO cardiovascular risk charts. [15] People who have already had a cardiovascular event (e.g., myocardial infarction) are at higher risk of another cardiovascular event. Assuming everyone was free of cardiovascular diseases could have underestimated the overall cardiovascular risk in our population. Third, high cardiovascular risk according to the local guidelines was based on the presence of some risk factors according to data availability; in other words, we could not include all possible risk factors. This limitation could have also underestimated the risk based on the local guidelines. Fourth, Peru has cities at sea level and at high altitude above the sea level (e.g., Puno at >3,800 meters above sea level). It is worth remembering this feature when interpreting and comparing results, particularly between regions at different altitude above the sea level because high altitude above the sea level could influence blood pressure.

Other potential limitation with relevant ramifications is that we used the office-based cardiovascular risk charts and not the laboratory-based cardiovascular risk charts, [15] because the ENDES does not collect biomarkers (e.g., total cholesterol). This limitation rests on the fact that the laboratory- and office-based models do not consistently identify the same individuals. The authors of the 2019 WHO cardiovascular risk charts described that, when the laboratory-based model was set at ≥20% cardiovascular risk and the office-based model was set at ≥10%, they identified ~97% of the same individuals; conversely, when the laboratory-based model was set at ≥20% cardiovascular risk and the office-based model was set at ≥20%, they identified ~65% and ~35% of the same men and women, respectively. [15] Had we used the laboratory-based model, the pool of eligible subjects would have increased (denominator), and at a fixed number of people receiving antihypertensive medication (numerator), the proportion of eligible subject receiving antihypertensive treatment would have decreased hence the differences between the local and international guidelines would have shortened. Even though our work benefited from large datasets and from the office-based cardiovascular risk charts currently endorsed by the WHO, [15] our results deservers further verification using the laboratory-based model. Peru needs a national survey with measured biomarkers not only to better quantify this treatment gap, but also to update means and prevalences of cardio-metabolic risk factors. With the last national survey with measured biomarkers conducted in 2005, [32] Peru does not have recent robust evidence to characterize the cardio-metabolic profile of its population.

## Conclusion

At the population level, between 2015 and 2020, the absolute cardiovascular risk was below 5% and less than one in a hundred people would have an absolute cardiovascular risk above 20% in Peru. At the national level there was a mild improvement in antihypertensive treatment allocation among eligible individuals, though in older women this rate has decreased. At the sub-national level, depending on whether local or WHO guidelines were followed, some regions showed improving rates of antihypertensive treatment allocation yet other showed decreasing rates. Peru needs to define a tool for surveillance of absolute cardiovascular risk and to monitor antihypertensive treatment allocation among high-risk people.

## Contributors

RMC-L conceived the idea, conducted the analysis and wrote the first draft of the manuscript. WCG-V conducted the analysis, edited and provided critical input to improve the manuscript. AB-O edited and provided critical input to improve the manuscript and research idea. All authors approved the submitted version.

## Declaration of interests

No conflict of interest to declare.

## Appendix Supplementary materials

Image, application 1

Image, application 2

Image, application 3

Image, application 4

Image, application 5

Image, application 6

Image, application 7

Image, application 8

Image, application 9

Image, application 10

Image, application 11

## References

[1] Whelton PK, Carey RM, Aronow WS (2017). ACC/AHA/AAPA/ABC/ACPM/AGS/APhA/ASH/ASPC/NMA/PCNA Guideline for the Prevention, Detection, Evaluation, and Management of High Blood Pressure in Adults: A Report of the American College of Cardiology/American Heart Association Task Force on Clinical Practice Guidelines. *Circulation* 2018.

[2] Ministerio de Salud Peru. Guía Técnica: Guía de Práctica Clínica para el Diagnóstico, Tratamiento y Control de la Enfermedad Hipertensiva. 2021 URL: https://www.gob.pe/institucion/minsa/normas-legales/195692-031-2015-minsa.

[3] Williams B, Mancia G, Spiering W (2018). ESC/ESH Guidelines for the management of arterial hypertension. *European heart journal* 2018.

[4] (2021). NICE National Institute for Health and Care Excellence. Hypertension in adults: diagnosis and management.

[5] World Health Organization. HEARTS Technical Package - Risk-based CVD management. URL: https://apps.who.int/iris/bitstream/handle/10665/333221/9789240001367-eng.pdf?ua=1 2021.

[6] Wolrd Health Organization. HEARTS Technical Package - Systems for monitoring. URL: https://apps.who.int/iris/bitstream/handle/10665/260423/WHO-NMH-NVI-18.5-eng.pdf?sequence=1. 2021.

[7] Jones DW, Whelton PK, Allen N (2021). Management of Stage 1 Hypertension in Adults With a Low 10-Year Risk for Cardiovascular Disease: Filling a Guidance Gap: A Scientific Statement From the. American Heart Association. *Hypertension (Dallas, Tex: 1979)*.

[8] Morales-Salinas A, Olsen MH, Kones R (2020). Second Consensus on Treatment of Patients Recently Diagnosed With Mild Hypertension and Low Cardiovascular Risk. Current problems in cardiology.

[9] Chow CK, Teo KK, Rangarajan S (2013). Prevalence, awareness, treatment, and control of hypertension in rural and urban communities in high-, middle-, and low-income countries. Jama.

[10] Geldsetzer P, Manne-Goehler J, Marcus ME (2019). The state of hypertension care in 44 low-income and middle-income countries: a cross-sectional study of nationally representative individual-level data from 1•1 million adults. Lancet (London, England).

[11] Kayima J, Wanyenze RK, Katamba A, Leontsini E, Nuwaha F. (2013). Hypertension awareness, treatment and control in Africa: a systematic review. BMC cardiovascular disorders.

[12] Risk Factor Collaboration NCD (2019). (NCD-RisC). Long-term and recent trends in hypertension awareness, treatment, and control in 12 high-income countries: an analysis of 123 nationally representative surveys. Lancet (London, England).

[13] Villarreal-Zegarra D, Carrillo-Larco RM, Bernabe-Ortiz A. (2020). Short-term trends in the prevalence, awareness, treatment, and control of arterial hypertension in Peru. Journal of human hypertension.

[14] Peiris D, Ghosh A, Manne-Goehler J (2021). Cardiovascular disease risk profile and management practices in 45 low-income and middle-income countries: A cross-sectional study of nationally representative individual-level survey data. PLoS medicine.

[15] WHO CVD Risk Chart Working Group (2019). World Health Organization cardiovascular disease risk charts: revised models to estimate risk in 21 global regions. The Lancet Global health.

[16] Package of essential noncommunicable (PEN) disease interventions for primary health care in low-resource setting. WHO PEN Protocol 1 Preventiion of HEart Attacks, Stroke and Kidney Disease through Integrated Management of Diabetes and Hypertension. URL: https://www.who.int/ncds/management/Protocol1_HeartAttack_strokes_kidneyDisease.pdf?ua=1. 2021.

[17] Law MR, Morris JK, Wald NJ. (2009). Use of blood pressure lowering drugs in the prevention of cardiovascular disease: meta-analysis of 147 randomised trials in the context of expectations from prospective epidemiological studies. BMJ (Clinical research ed).

[18] University of Cambridge (2021). Cardiovascular Epidemiology Unit. Programs.

[19] Mancia G, Fagard R, Narkiewicz K (2013). ESH/ESC guidelines for the management of arterial hypertension: the Task Force for the Management of Arterial Hypertension of the European Society of Hypertension (ESH) and of the European Society of Cardiology (ESC). *European heart journal* 2013.

[20] Ministerio de Salud Peru. Guía de Práctica Clínica para prevención y Control de la Enfermedad Hipertensiva en el Primer Nivel de Atención. URL: https://www.gob.pe/institucion/minsa/normas-legales/246435-491-2009-minsa.2021.

[21] (2017). Instituto nacional de informatica y estadistica. Censos nacionales.

[22] Hajifathalian K, Ueda P, Lu Y (2015). A novel risk score to predict cardiovascular disease risk in national populations (Globorisk): a pooled analysis of prospective cohorts and health examination surveys. The lancet Diabetes & endocrinology.

[23] Ueda P, Woodward M, Lu Y (2017). Laboratory-based and office-based risk scores and charts to predict 10-year risk of cardiovascular disease in 182 countries: a pooled analysis of prospective cohorts and health surveys. The lancet Diabetes & endocrinology.

[24] Karmali KN, Persell SD, Perel P, Lloyd-Jones DM, Berendsen MA, Huffman MD (2017). Risk scoring for the primary prevention of cardiovascular disease. The Cochrane database of systematic reviews.

[25] Muñoz OM, Rodríguez NI, Ruiz A, Rondón M. (2014). Validación de los modelos de predicción de Framingham y PROCAM como estimadores del riesgo cardiovascular en una población colombiana. Rev colomb cardiol.

[26] Icaza G, Núñez L, Marrugat J (2009). [Estimation of coronary heart disease risk in Chilean subjects based on adapted Framingham equations]. Revista medica de Chile.

[27] Cohorts Consortium of Latin America and the Caribbean (CC-LAC) (2020). Cohort Profile: The Cohorts Consortium of Latin America and the Caribbean (CC-LAC). International journal of epidemiology.

[28] Carrillo-Larco RM, Altez-Fernandez C, Pacheco-Barrios N (2019). Cardiovascular Disease Prognostic Models in Latin America and the Caribbean: A Systematic Review. Global heart.

[29] Attaei MW, Khatib R, McKee M (2017). Availability and affordability of blood pressure-lowering medicines and the effect on blood pressure control in high-income, middle-income, and low-income countries: an analysis of the PURE study data. The Lancet Public health.

[30] Fletcher BR, Hartmann-Boyce J, Hinton L, McManus RJ. (2015). The Effect of Self-Monitoring of Blood Pressure on Medication Adherence and Lifestyle Factors: A Systematic Review and Meta-Analysis. American journal of hypertension.

[31] López-Jaramillo P, Barbosa E, Molina DI (2019). Latin American Consensus on the management of hypertension in the patient with diabetes and the metabolic syndrome. Journal of hypertension.

[32] Ministerio de Salud. (2006). Encuesta Nacional de Indicadores Nutricionales, Bioquimicos, Socioeconomicos y Culturales Relacionados con las Enfermedades Cronicas Degenerativas. Lima: MINSA.

