# Supplementary material for: Risk-based antihypertensive treatment allocation in Peru: comparison of local and international guidelines analysing national health surveys between 2015-2020
Source: Lancet Reg Health Am. 2021 Jul 26;1:100022. doi: 10.1016/j.lana.2021.100022 (PMC8438602; doi:10.1016/j.lana.2021.100022)
Supplement: Supplementary file 1 [file mmc1.pdf]

Risk-based antihypertensive treatment allocation in Peru: comparison of local and international guidelines analysing national health surveys between 2015-2020

Correspondence author:

Rodrigo M Carrillo-Larco, MD

Department of Epidemiology and Biostatistics

School of Public Health

Imperial College London

rcarrill@ic.ac.uk

Supplementary Table 1. Number of observations in each possible answer to the questions about self-reported hypertension diagnosis, self-reported antihypertensive treatment and self-reported diabetes diagnosis. 3

Supplementary Table 2. Number of excluded observations due to implausible ranges in systolic blood pressure and diastolic blood pressure 4

Supplementary Table 3. Absolute cardiovascular risk at the national level by year 5

Supplementary Table 4. Proportion of eligible and non-eligible subjects by guideline 6

Supplementary Table 5. Proportion (%) in each category of absolute cardiovascular risk by region, sex and year 7

Supplementary Table 6. Proportion of eligible and non-eligible subjects for antihypertensive treatment according to the selected guidelines by year sex, and age group (national level) 59

Supplementary Table 7. Proportion of eligible and non-eligible subjects for antihypertensive treatment according to the selected guidelines by year and region (sub-national level) 63

Supplementary Figure 1. Percentage of people receiving antihypertensive medication among not eligible subjects by sex and age at the national level 84

Supplementary Figure 2. Percentage of people receiving antihypertensive medication among eligible subjects by sex and macro-region 85

Supplementary Figure 3. Percentage of people receiving antihypertensive medication among eligible subjects by sex and macro-region 86

Supplementary Figure 4. Percentage of people receiving antihypertensive medication among eligible subjects at the sub-national level 87

Supplementary Figure 5. Percentage of people receiving antihypertensive medication among not eligible subjects at the sub-national level 88

STROBE Statement—Checklist of items that should be included in reports of *cross-sectional studies* 89

Supplementary Table 1. Number of observations in each possible answer to the questions about self-reported hypertension diagnosis, self-reported antihypertensive treatment and self-reported diabetes diagnosis.

	Yes	No	Does not know/does not remember
	**Self-reported hypertension (absolute number of observations)**		
2015	2,801	30,072	33
2016	2,559	29,074	26
2017	2,556	29,913	45
2018	2,894	30,875	25
2019	2,892	29,983	46
2020	2,708	29,091	28
	**Self-reported antihypertensive treatment (absolute number of observations)**		
2015	1,712	1,085	1
2016	1,669	889	1
2017	1,623	930	3
2018	1,807	1,084	3
2019	1,906	981	5
2020	1,783	923	2
	**Self-reported diabetes (absolute number of observations)**		
2015	804	32,086	16
2016	816	30,819	24
2017	865	31,619	30
2018	931	32,835	28
2019	966	31,922	33
2020	1,157	30,629	39

Supplementary Table 2. Number of excluded observations due to implausible ranges in systolic blood pressure and diastolic blood pressure

	1st systolic blood pressure < 70	1st systolic blood pressure > 270	2nd systolic blood pressure < 70	2nd systolic blood pressure > 270
2015	1	0	1	0
2016	0	0	0	0
2017	0	0	0	0
2018	2	0	1	0
2019	4	0	3	0
2020	1	0	3	0

	1st diastolic blood pressure < 30	1st diastolic blood pressure > 150	2nd diastolic blood pressure < 30	2nd diastolic blood pressure > 150
2015	0	2	0	1
2016	0	0	0	0
2017	0	0	0	0
2018	0	0	0	0
2019	0	0	0	0
2020	0	0	0	0

Supplementary Table 3. Absolute cardiovascular risk at the national level by year

Year	Absolute cardiovascular risk (95% confidence interval)
2015	3.5% (3.4%-3.6%)
2016	3.5% (3.4%-3.6%)
2017	3.6% (3.5%-3.6%)
2018	3.7% (3.6%-3.7%)
2019	3.6% (3.5%-3.7%)
2020	3.6% (3.5%-3.7%)

Supplementary Table 4. Proportion of eligible and non-eligible subjects by guideline

	Eligible for antihypertensive treatment	MINSA (%) (95% confidence interval)	WHO (%) (95% confidence interval)
2015	No	84.0 (83.1-84.8)	93.1 (92.5-93.6)
2016	No	83.7 (82.9-84.5)	92.4 (91.8-93.0)
2017	No	82.6 (81.6-83.6)	92.1 (91.3-92.7)
2018	No	80.9 (80.1-81.8)	91.2 (90.5-91.8)
2019	No	81.7 (80.8-82.6)	91.4 (90.7-92.1)
2020	No	79.0 (77.8-80.2)	91.0 (90.2-91.8)
2015	Yes	16.0 (15.2-16.9)	7.0 (6.4-7.5)
2016	Yes	16.3 (15.5-17.1)	7.6 (7.0-8.2)
2017	Yes	17.4 (16.4-18.4)	7.9 (7.3-8.7)
2018	Yes	19.1 (18.2-19.9)	8.9 (8.2-9.5)
2019	Yes	18.3 (17.4-19.2)	8.6 (7.9-9.3)
2020	Yes	21.0 (19.8-22.2)	9.0 (8.2-9.8)

	No	Yes
	Eligible MINSA (observed data)	
2015	17,712	2,775
2016	17,491	2,790
2017	17,831	2,956
2018	18,848	3,398
2019	18,304	3,194
2020	12,326	2,443
	Eligible WHO (observed data)	
2015	19,348	1,139
2016	19,064	1,217
2017	19,442	1,345
2018	20,793	1,444
2019	20,083	1,415
2020	13,738	1,031

Supplementary Table 5. Proportion (%) in each category of absolute cardiovascular risk by region, sex and year

Region	Sex	Year	Risk group	Proportion	Proportion lower limit	Proportion upper limit	Numerator	Denominator
Amazonas	Men	2015	<5	78.32	73.78	82.27	313	385
Ancash	Men	2015	<5	74.96	69.19	79.96	276	347
Apurimac	Men	2015	<5	77.43	72.21	81.92	267	329
Arequipa	Men	2015	<5	76.67	70.18	82.11	287	348
Ayacucho	Men	2015	<5	76.64	70.67	81.7	343	422
Cajamarca	Men	2015	<5	79.03	73.88	83.39	278	325
Callao	Men	2015	<5	70.53	64.37	76.02	269	347
Cusco	Men	2015	<5	78.5	72.99	83.14	288	347
Huancavelica	Men	2015	<5	79.12	74.48	83.12	259	323
Huanuco	Men	2015	<5	77.94	72.5	82.57	322	386
Ica	Men	2015	<5	73.95	68.63	78.66	287	362
Junin	Men	2015	<5	80.04	74.04	84.94	258	314
La Libertad	Men	2015	<5	77.15	69.63	83.26	249	311
Lambayeque	Men	2015	<5	72	66.45	76.94	269	365
Lima	Men	2015	<5	75.23	71.61	78.52	902	1120
Loreto	Men	2015	<5	80.33	74.85	84.85	318	392
Madre de Dios	Men	2015	<5	82.7	77.02	87.21	286	337
Moquegua	Men	2015	<5	71.1	64.09	77.23	265	350
Pasco	Men	2015	<5	80.62	73.45	86.22	288	332
Piura	Men	2015	<5	70.3	64	75.91	283	361
Puno	Men	2015	<5	78.65	73.12	83.31	251	322
San Martin	Men	2015	<5	78.67	73.29	83.22	350	417
Tacna	Men	2015	<5	75.27	68.32	81.12	294	355
Tumbes	Men	2015	<5	75.91	70.69	80.45	344	412
Ucayali	Men	2015	<5	79.49	73.86	84.17	282	349

Amazonas	Women	2015	<5	82.83	78.58	86.39	349	406
Ancash	Women	2015	<5	79.73	75.03	83.74	345	421
Apurimac	Women	2015	<5	75.57	69.26	80.94	264	337
Arequipa	Women	2015	<5	86.05	79.86	90.56	359	417
Ayacucho	Women	2015	<5	80.3	74.62	84.97	395	461
Cajamarca	Women	2015	<5	84.78	79.5	88.9	326	381
Callao	Women	2015	<5	81.2	76.09	85.43	371	437
Cusco	Women	2015	<5	81.35	76.06	85.69	295	363
Huancavelica	Women	2015	<5	77.85	72.64	82.32	305	379
Huanuco	Women	2015	<5	81.64	76.03	86.18	395	459
Ica	Women	2015	<5	79.83	75.16	83.82	359	441
Junin	Women	2015	<5	83.13	77.93	87.3	346	410
La Libertad	Women	2015	<5	81.45	76.45	85.59	377	438
Lambayeque	Women	2015	<5	83.28	78.71	87.03	366	426
Lima	Women	2015	<5	82.41	79.43	85.04	1079	1278
Loreto	Women	2015	<5	88.84	84.8	91.91	315	355
Madre de Dios	Women	2015	<5	91.38	86.41	94.65	306	330
Moquegua	Women	2015	<5	85	80.28	88.74	335	395
Pasco	Women	2015	<5	83.61	78.64	87.61	310	367
Piura	Women	2015	<5	79.29	73.69	83.95	346	414
Puno	Women	2015	<5	80.31	75.4	84.44	321	395
San Martin	Women	2015	<5	83.29	77.94	87.55	314	369
Tacna	Women	2015	<5	83.02	77.36	87.5	319	376
Tumbes	Women	2015	<5	84.98	80.33	88.68	333	376
Ucayali	Women	2015	<5	86.85	82.01	90.54	352	398
Amazonas	Men	2015	5-9	13.73	10.36	17.97	48	385
Ancash	Men	2015	5-9	16.78	12.13	22.75	43	347
Apurimac	Men	2015	5-9	16.61	12.68	21.46	44	329
Arequipa	Men	2015	5-9	13.49	9.24	19.27	35	348

Ayacucho	Men	2015	5-9	14.31	10.39	19.38	45	422
Cajamarca	Men	2015	5-9	12.41	8.66	17.48	26	325
Callao	Men	2015	5-9	19.12	15	24.06	52	347
Cusco	Men	2015	5-9	12.96	9.72	17.08	42	347
Huancavelica	Men	2015	5-9	13.06	10	16.87	41	323
Huanuco	Men	2015	5-9	14.28	10.36	19.36	36	386
Ica	Men	2015	5-9	18.79	14.62	23.81	55	362
Junin	Men	2015	5-9	13.87	9.94	19.02	42	314
La Libertad	Men	2015	5-9	12.59	8.83	17.64	33	311
Lambayeque	Men	2015	5-9	18.74	14.18	24.35	65	365
Lima	Men	2015	5-9	14.29	11.84	17.14	126	1120
Loreto	Men	2015	5-9	12.42	9.26	16.45	51	392
Madre de Dios	Men	2015	5-9	13.63	9.34	19.46	38	337
Moquegua	Men	2015	5-9	17.38	11.73	24.99	49	350
Pasco	Men	2015	5-9	12.06	8.18	17.42	28	332
Piura	Men	2015	5-9	20.37	15.1	26.9	54	361
Puno	Men	2015	5-9	15.19	11.37	20	52	322
San Martin	Men	2015	5-9	13.74	10.59	17.63	46	417
Tacna	Men	2015	5-9	13.79	9.64	19.35	35	355
Tumbes	Men	2015	5-9	15.55	11.69	20.39	48	412
Ucayali	Men	2015	5-9	13.72	9.73	18.99	46	349
Amazonas	Women	2015	5-9	9.32	6.57	13.06	33	406
Ancash	Women	2015	5-9	12.96	9.55	17.35	49	421
Apurimac	Women	2015	5-9	16.84	12.27	22.68	51	337
Arequipa	Women	2015	5-9	8.88	5.99	12.98	42	417
Ayacucho	Women	2015	5-9	12.39	8.71	17.33	41	461
Cajamarca	Women	2015	5-9	10.76	7.63	14.97	41	381
Callao	Women	2015	5-9	13.76	10.48	17.86	52	437
Cusco	Women	2015	5-9	13.6	10.21	17.89	47	363

Huancavelica	Women	2015	5-9	15.04	11.34	19.68	51	379
Huanuco	Women	2015	5-9	12.94	8.93	18.39	46	459
Ica	Women	2015	5-9	13.24	10.22	16.99	56	441
Junin	Women	2015	5-9	14.2	10.49	18.94	49	410
La Libertad	Women	2015	5-9	12.8	9.29	17.37	42	438
Lambayeque	Women	2015	5-9	12.78	9.55	16.9	45	426
Lima	Women	2015	5-9	10.94	8.88	13.4	132	1278
Loreto	Women	2015	5-9	9.24	6.35	13.27	29	355
Madre de Dios	Women	2015	5-9	5.99	3.48	10.13	17	330
Moquegua	Women	2015	5-9	10.78	7.64	15	44	395
Pasco	Women	2015	5-9	11.56	8.48	15.57	38	367
Piura	Women	2015	5-9	14.81	10.97	19.71	46	414
Puno	Women	2015	5-9	13.05	9.44	17.76	47	395
San Martin	Women	2015	5-9	13.1	9.46	17.87	37	369
Tacna	Women	2015	5-9	11.33	8.13	15.59	38	376
Tumbes	Women	2015	5-9	12.56	9.09	17.11	34	376
Ucayali	Women	2015	5-9	10.34	7.54	14.01	39	398
Amazonas	Men	2015	10-19	6.42	4.21	9.68	20	385
Ancash	Men	2015	10-19	7.66	4.48	12.79	25	347
Apurimac	Men	2015	10-19	5.96	3.47	10.05	18	329
Arequipa	Men	2015	10-19	9.35	5.98	14.34	25	348
Ayacucho	Men	2015	10-19	7.77	5.39	11.08	28	422
Cajamarca	Men	2015	10-19	7.72	4.81	12.17	19	325
Callao	Men	2015	10-19	8.8	5.5	13.79	23	347
Cusco	Men	2015	10-19	7.25	3.98	12.85	15	347
Huancavelica	Men	2015	10-19	7.51	4.67	11.84	21	323
Huanuco	Men	2015	10-19	6.31	4.32	9.12	23	386
Ica	Men	2015	10-19	6.66	4.03	10.81	18	362
Junin	Men	2015	10-19	5.46	2.92	9.97	12	314

La Libertad	Men	2015	10-19	9.27	5.73	14.68	26	311
Lambayeque	Men	2015	10-19	7.88	5.55	11.06	27	365
Lima	Men	2015	10-19	8.98	6.83	11.73	78	1120
Loreto	Men	2015	10-19	5.94	3.73	9.33	20	392
Madre de Dios	Men	2015	10-19	3.67	2.14	6.24	13	337
Moquegua	Men	2015	10-19	10.81	7.48	15.38	34	350
Pasco	Men	2015	10-19	6.71	3.33	13.05	14	332
Piura	Men	2015	10-19	7.28	3.98	12.95	18	361
Puno	Men	2015	10-19	6.16	3.84	9.74	19	322
San Martin	Men	2015	10-19	6.26	3.62	10.61	17	417
Tacna	Men	2015	10-19	10.05	6.32	15.6	23	355
Tumbes	Men	2015	10-19	7.78	4.69	12.62	18	412
Ucayali	Men	2015	10-19	6.47	4.02	10.24	20	349
Amazonas	Women	2015	10-19	7.84	5.27	11.52	24	406
Ancash	Women	2015	10-19	7.31	4.95	10.67	27	421
Apurimac	Women	2015	10-19	7.19	4.61	11.05	21	337
Arequipa	Women	2015	10-19	5.07	2.88	8.77	16	417
Ayacucho	Women	2015	10-19	7.21	4.5	11.35	24	461
Cajamarca	Women	2015	10-19	3.66	1.92	6.87	12	381
Callao	Women	2015	10-19	4.6	2.36	8.75	12	437
Cusco	Women	2015	10-19	4.72	2.89	7.62	20	363
Huancavelica	Women	2015	10-19	6.97	4.16	11.44	22	379
Huanuco	Women	2015	10-19	4.65	2.69	7.92	16	459
Ica	Women	2015	10-19	6.05	3.89	9.3	24	441
Junin	Women	2015	10-19	2.67	1.55	4.55	15	410
La Libertad	Women	2015	10-19	5.54	3.35	9.02	18	438
Lambayeque	Women	2015	10-19	3.79	2.19	6.47	14	426
Lima	Women	2015	10-19	5.77	4.28	7.73	61	1278
Loreto	Women	2015	10-19	1.92	1.04	3.51	11	355

Madre de Dios	Women	2015	10-19	2.17	0.82	5.61	5	330
Moquegua	Women	2015	10-19	3.7	2.22	6.12	15	395
Pasco	Women	2015	10-19	4.83	2.98	7.73	19	367
Piura	Women	2015	10-19	5.9	3.67	9.35	22	414
Puno	Women	2015	10-19	6.64	4.38	9.93	27	395
San Martin	Women	2015	10-19	3.61	1.83	7.02	18	369
Tacna	Women	2015	10-19	5.65	3.31	9.48	19	376
Tumbes	Women	2015	10-19	2.46	1.16	5.16	9	376
Ucayali	Women	2015	10-19	2.81	1.31	5.92	7	398
Amazonas	Men	2015	20-29	1.53	0.57	4.02	4	385
Ancash	Men	2015	20-29	0.6	0.15	2.39	3	347
Apurimac	Men	2015	20-29	0	0	0	0	329
Arequipa	Men	2015	20-29	0.49	0.07	3.37	1	348
Ayacucho	Men	2015	20-29	1.28	0.55	2.93	6	422
Cajamarca	Men	2015	20-29	0.84	0.2	3.41	2	325
Callao	Men	2015	20-29	1.54	0.49	4.76	3	347
Cusco	Men	2015	20-29	1.3	0.25	6.36	2	347
Huancavelica	Men	2015	20-29	0.31	0.07	1.41	2	323
Huanuco	Men	2015	20-29	1.47	0.55	3.85	5	386
Ica	Men	2015	20-29	0.6	0.15	2.39	2	362
Junin	Men	2015	20-29	0.64	0.16	2.57	2	314
La Libertad	Men	2015	20-29	0.63	0.15	2.56	2	311
Lambayeque	Men	2015	20-29	1.38	0.46	4.13	4	365
Lima	Men	2015	20-29	1.37	0.72	2.59	13	1120
Loreto	Men	2015	20-29	1.32	0.37	4.55	3	392
Madre de Dios	Men	2015	20-29	0	0	0	0	337
Moquegua	Men	2015	20-29	0.71	0.17	2.9	2	350
Pasco	Men	2015	20-29	0.61	0.15	2.44	2	332
Piura	Men	2015	20-29	2.05	0.86	4.78	6	361

Puno	Men	2015	20-29	0	0	0	0	322
San Martin	Men	2015	20-29	1.33	0.48	3.64	4	417
Tacna	Men	2015	20-29	0.89	0.25	3.08	3	355
Tumbes	Men	2015	20-29	0.76	0.19	3.03	2	412
Ucayali	Men	2015	20-29	0.32	0.05	2.26	1	349
Amazonas	Women	2015	20-29	0	0	0	0	406
Ancash	Women	2015	20-29	0	0	0	0	421
Apurimac	Women	2015	20-29	0.4	0.06	2.85	1	337
Arequipa	Women	2015	20-29	0	0	0	0	417
Ayacucho	Women	2015	20-29	0.1	0.01	0.71	1	461
Cajamarca	Women	2015	20-29	0.79	0.19	3.16	2	381
Callao	Women	2015	20-29	0.44	0.11	1.78	2	437
Cusco	Women	2015	20-29	0.33	0.05	2.34	1	363
Huancavelica	Women	2015	20-29	0.14	0.02	1.01	1	379
Huanuco	Women	2015	20-29	0.59	0.08	4.2	1	459
Ica	Women	2015	20-29	0.88	0.22	3.43	2	441
Junin	Women	2015	20-29	0	0	0	0	410
La Libertad	Women	2015	20-29	0.21	0.03	1.53	1	438
Lambayeque	Women	2015	20-29	0	0	0	0	426
Lima	Women	2015	20-29	0.78	0.29	2.11	5	1278
Loreto	Women	2015	20-29	0	0	0	0	355
Madre de Dios	Women	2015	20-29	0.29	0.04	2.1	1	330
Moquegua	Women	2015	20-29	0.52	0.07	3.56	1	395
Pasco	Women	2015	20-29	0	0	0	0	367
Piura	Women	2015	20-29	0	0	0	0	414
Puno	Women	2015	20-29	0	0	0	0	395
San Martin	Women	2015	20-29	0	0	0	0	369
Tacna	Women	2015	20-29	0	0	0	0	376
Tumbes	Women	2015	20-29	0	0	0	0	376

Ucayali	Women	2015	20-29	0	0	0	0	398
Amazonas	Men	2015	30+	0	0	0	0	385
Ancash	Men	2015	30+	0	0	0	0	347
Apurimac	Men	2015	30+	0	0	0	0	329
Arequipa	Men	2015	30+	0	0	0	0	348
Ayacucho	Men	2015	30+	0	0	0	0	422
Cajamarca	Men	2015	30+	0	0	0	0	325
Callao	Men	2015	30+	0	0	0	0	347
Cusco	Men	2015	30+	0	0	0	0	347
Huancavelica	Men	2015	30+	0	0	0	0	323
Huanuco	Men	2015	30+	0	0	0	0	386
Ica	Men	2015	30+	0	0	0	0	362
Junin	Men	2015	30+	0	0	0	0	314
La Libertad	Men	2015	30+	0.35	0.05	2.51	1	311
Lambayeque	Men	2015	30+	0	0	0	0	365
Lima	Men	2015	30+	0.13	0.02	0.89	1	1120
Loreto	Men	2015	30+	0	0	0	0	392
Madre de Dios	Men	2015	30+	0	0	0	0	337
Moquegua	Men	2015	30+	0	0	0	0	350
Pasco	Men	2015	30+	0	0	0	0	332
Piura	Men	2015	30+	0	0	0	0	361
Puno	Men	2015	30+	0	0	0	0	322
San Martin	Men	2015	30+	0	0	0	0	417
Tacna	Men	2015	30+	0	0	0	0	355
Tumbes	Men	2015	30+	0	0	0	0	412
Ucayali	Men	2015	30+	0	0	0	0	349
Amazonas	Women	2015	30+	0	0	0	0	406
Ancash	Women	2015	30+	0	0	0	0	421
Apurimac	Women	2015	30+	0	0	0	0	337

Arequipa	Women	2015	30+	0	0	0	0	417
Ayacucho	Women	2015	30+	0	0	0	0	461
Cajamarca	Women	2015	30+	0	0	0	0	381
Callao	Women	2015	30+	0	0	0	0	437
Cusco	Women	2015	30+	0	0	0	0	363
Huancavelica	Women	2015	30+	0	0	0	0	379
Huanuco	Women	2015	30+	0.17	0.02	1.25	1	459
Ica	Women	2015	30+	0	0	0	0	441
Junin	Women	2015	30+	0	0	0	0	410
La Libertad	Women	2015	30+	0	0	0	0	438
Lambayeque	Women	2015	30+	0.15	0.02	1.09	1	426
Lima	Women	2015	30+	0.11	0.01	0.76	1	1278
Loreto	Women	2015	30+	0	0	0	0	355
Madre de Dios	Women	2015	30+	0.17	0.02	1.23	1	330
Moquegua	Women	2015	30+	0	0	0	0	395
Pasco	Women	2015	30+	0	0	0	0	367
Piura	Women	2015	30+	0	0	0	0	414
Puno	Women	2015	30+	0	0	0	0	395
San Martin	Women	2015	30+	0	0	0	0	369
Tacna	Women	2015	30+	0	0	0	0	376
Tumbes	Women	2015	30+	0	0	0	0	376
Ucayali	Women	2015	30+	0	0	0	0	398
Amazonas	Men	2016	<5	80.01	75.74	83.69	301	381
Ancash	Men	2016	<5	73.63	68.04	78.55	246	314
Apurimac	Men	2016	<5	76.47	70.4	81.62	269	330
Arequipa	Men	2016	<5	74.98	68.33	80.64	279	355
Ayacucho	Men	2016	<5	77.68	72.2	82.35	297	392
Cajamarca	Men	2016	<5	78.84	73.02	83.68	249	317
Callao	Men	2016	<5	69.17	61.73	75.73	248	325

Cusco	Men	2016	<5	75.81	70.96	80.07	295	365
Huancavelica	Men	2016	<5	78.39	72.65	83.2	238	298
Huanuco	Men	2016	<5	81.68	76.73	85.76	312	374
Ica	Men	2016	<5	73.53	65.97	79.92	270	354
Junin	Men	2016	<5	76.98	71.59	81.61	243	308
La Libertad	Men	2016	<5	76.82	71.15	81.65	259	325
Lambayeque	Men	2016	<5	72.91	67.12	78.02	285	366
Lima	Men	2016	<5	74.36	70.4	77.95	816	1061
Loreto	Men	2016	<5	76.96	72.18	81.14	297	378
Madre de Dios	Men	2016	<5	82.29	76.91	86.63	273	329
Moquegua	Men	2016	<5	73.06	65.09	79.77	265	337
Pasco	Men	2016	<5	80.44	74.8	85.07	275	334
Piura	Men	2016	<5	75.91	70.01	80.97	270	346
Puno	Men	2016	<5	77.07	70.06	82.84	235	303
San Martin	Men	2016	<5	82.28	76.97	86.57	356	417
Tacna	Men	2016	<5	76.11	71.2	80.41	285	362
Tumbes	Men	2016	<5	75.76	69.73	80.92	304	378
Ucayali	Men	2016	<5	80.35	75.08	84.74	261	317
Amazonas	Women	2016	<5	81.97	75.55	86.99	348	421
Ancash	Women	2016	<5	78.52	71.72	84.06	327	403
Apurimac	Women	2016	<5	79.96	74.28	84.64	300	356
Arequipa	Women	2016	<5	84.09	78.12	88.66	327	371
Ayacucho	Women	2016	<5	80.1	75.23	84.21	425	517
Cajamarca	Women	2016	<5	81.64	76.54	85.84	290	353
Callao	Women	2016	<5	81.81	77.22	85.65	361	424
Cusco	Women	2016	<5	81.85	75.08	87.1	301	363
Huancavelica	Women	2016	<5	79.26	74.18	83.56	313	395
Huanuco	Women	2016	<5	82.89	78.45	86.56	420	488
Ica	Women	2016	<5	82.46	78.24	86.01	336	409

Junin	Women	2016	<5	82.92	78.5	86.58	381	444
La Libertad	Women	2016	<5	82.95	77.29	87.43	315	369
Lambayeque	Women	2016	<5	82	77.27	85.93	394	468
Lima	Women	2016	<5	81.66	78.37	84.54	1027	1209
Loreto	Women	2016	<5	83.93	78.23	88.36	328	369
Madre de Dios	Women	2016	<5	91.68	85.18	95.48	325	343
Moquegua	Women	2016	<5	81.85	76.2	86.41	361	411
Pasco	Women	2016	<5	85.85	81.39	89.38	318	370
Piura	Women	2016	<5	81.44	76.97	85.2	372	434
Puno	Women	2016	<5	81.91	76.3	86.44	333	416
San Martin	Women	2016	<5	82.46	75.97	87.49	302	351
Tacna	Women	2016	<5	84.1	77.85	88.84	380	428
Tumbes	Women	2016	<5	85.03	79.31	89.37	356	391
Ucayali	Women	2016	<5	88.51	83.64	92.07	381	412
Amazonas	Men	2016	5-9	11.63	8.42	15.86	43	381
Ancash	Men	2016	5-9	15.31	11.57	20	47	314
Apurimac	Men	2016	5-9	14.59	10.44	20.02	39	330
Arequipa	Men	2016	5-9	14.64	10.46	20.11	48	355
Ayacucho	Men	2016	5-9	13.04	9.94	16.93	56	392
Cajamarca	Men	2016	5-9	12.44	8.86	17.2	42	317
Callao	Men	2016	5-9	19.12	13.89	25.72	47	325
Cusco	Men	2016	5-9	16.19	12.36	20.92	47	365
Huancavelica	Men	2016	5-9	14.29	10.59	19.02	42	298
Huanuco	Men	2016	5-9	11.86	8.55	16.22	39	374
Ica	Men	2016	5-9	13.12	9.16	18.45	44	354
Junin	Men	2016	5-9	14.45	10.17	20.11	44	308
La Libertad	Men	2016	5-9	13.4	9.62	18.37	37	325
Lambayeque	Men	2016	5-9	16.14	11.78	21.74	47	366
Lima	Men	2016	5-9	14.13	11.53	17.21	131	1061

Loreto	Men	2016	5-9	16.29	12.54	20.89	59	378
Madre de Dios	Men	2016	5-9	10.88	7.78	15.02	37	329
Moquegua	Men	2016	5-9	16.79	12.31	22.48	47	337
Pasco	Men	2016	5-9	12.05	8.35	17.1	36	334
Piura	Men	2016	5-9	15.67	11.54	20.94	49	346
Puno	Men	2016	5-9	17.63	12.54	24.22	48	303
San Martin	Men	2016	5-9	10.14	6.92	14.62	35	417
Tacna	Men	2016	5-9	15.86	12.13	20.46	48	362
Tumbes	Men	2016	5-9	14.37	9.71	20.77	44	378
Ucayali	Men	2016	5-9	15.03	11.12	20	42	317
Amazonas	Women	2016	5-9	10.46	6.83	15.7	43	421
Ancash	Women	2016	5-9	15.82	11.51	21.37	58	403
Apurimac	Women	2016	5-9	15.3	11.27	20.43	43	356
Arequipa	Women	2016	5-9	11.57	8.04	16.38	36	371
Ayacucho	Women	2016	5-9	13.84	10.34	18.29	65	517
Cajamarca	Women	2016	5-9	11.94	8.35	16.79	40	353
Callao	Women	2016	5-9	11.46	8.35	15.53	45	424
Cusco	Women	2016	5-9	13.66	9.21	19.79	49	363
Huancavelica	Women	2016	5-9	13.95	10.35	18.56	59	395
Huanuco	Women	2016	5-9	10.48	7.52	14.41	43	488
Ica	Women	2016	5-9	10.39	7.72	13.84	45	409
Junin	Women	2016	5-9	13.29	10.22	17.12	48	444
La Libertad	Women	2016	5-9	12.99	8.89	18.6	36	369
Lambayeque	Women	2016	5-9	12.16	8.9	16.39	54	468
Lima	Women	2016	5-9	13.13	10.7	16	131	1209
Loreto	Women	2016	5-9	12.08	8.34	17.17	33	369
Madre de Dios	Women	2016	5-9	5.86	3.1	10.79	14	343
Moquegua	Women	2016	5-9	14.46	10.21	20.08	41	411
Pasco	Women	2016	5-9	7.84	5.37	11.32	29	370

Piura	Women	2016	5-9	13.7	10.7	17.36	46	434
Puno	Women	2016	5-9	12.82	9.39	17.28	54	416
San Martin	Women	2016	5-9	12.42	8.6	17.6	35	351
Tacna	Women	2016	5-9	10.81	7.77	14.83	34	428
Tumbes	Women	2016	5-9	9.98	6.57	14.88	25	391
Ucayali	Women	2016	5-9	7.83	4.76	12.61	22	412
Amazonas	Men	2016	10-19	7.3	5.2	10.15	32	381
Ancash	Men	2016	10-19	10.25	6.22	16.46	19	314
Apurimac	Men	2016	10-19	8.95	5.64	13.89	22	330
Arequipa	Men	2016	10-19	8.81	5.62	13.56	24	355
Ayacucho	Men	2016	10-19	9	6.31	12.67	37	392
Cajamarca	Men	2016	10-19	7.27	4.86	10.74	23	317
Callao	Men	2016	10-19	10.34	6.73	15.56	27	325
Cusco	Men	2016	10-19	6.62	4.12	10.45	19	365
Huancavelica	Men	2016	10-19	7.15	4.41	11.4	17	298
Huanuco	Men	2016	10-19	5.84	3.65	9.23	21	374
Ica	Men	2016	10-19	12.74	8.36	18.94	37	354
Junin	Men	2016	10-19	7.55	4.58	12.2	19	308
La Libertad	Men	2016	10-19	8.18	5.3	12.41	24	325
Lambayeque	Men	2016	10-19	9.72	6.82	13.68	31	366
Lima	Men	2016	10-19	10.4	8.31	12.94	102	1061
Loreto	Men	2016	10-19	5.46	3.35	8.76	18	378
Madre de Dios	Men	2016	10-19	5.62	3.03	10.21	16	329
Moquegua	Men	2016	10-19	9.45	5.55	15.64	22	337
Pasco	Men	2016	10-19	6.98	4.55	10.57	22	334
Piura	Men	2016	10-19	7.05	4.54	10.8	21	346
Puno	Men	2016	10-19	4.67	2.54	8.41	18	303
San Martin	Men	2016	10-19	6.93	4.52	10.48	23	417
Tacna	Men	2016	10-19	7.3	4.99	10.58	26	362

Tumbes	Men	2016	10-19	9.34	6.3	13.62	28	378
Ucayali	Men	2016	10-19	4.05	2.11	7.65	12	317
Amazonas	Women	2016	10-19	7.25	4.41	11.69	29	421
Ancash	Women	2016	10-19	5.54	3.07	9.81	17	403
Apurimac	Women	2016	10-19	4.75	2.64	8.37	13	356
Arequipa	Women	2016	10-19	4.34	1.87	9.75	8	371
Ayacucho	Women	2016	10-19	5.8	3.75	8.86	26	517
Cajamarca	Women	2016	10-19	6.13	3.98	9.33	22	353
Callao	Women	2016	10-19	6.73	3.97	11.19	18	424
Cusco	Women	2016	10-19	4.49	2.44	8.12	13	363
Huancavelica	Women	2016	10-19	6.1	3.76	9.75	20	395
Huanuco	Women	2016	10-19	6.06	4.15	8.78	24	488
Ica	Women	2016	10-19	6.7	4.19	10.56	27	409
Junin	Women	2016	10-19	3.79	2.11	6.71	15	444
La Libertad	Women	2016	10-19	3.85	2.27	6.47	17	369
Lambayeque	Women	2016	10-19	5.67	3.21	9.82	19	468
Lima	Women	2016	10-19	5.01	3.61	6.92	47	1209
Loreto	Women	2016	10-19	3.99	1.81	8.59	8	369
Madre de Dios	Women	2016	10-19	1.9	0.51	6.8	3	343
Moquegua	Women	2016	10-19	3.68	1.8	7.38	9	411
Pasco	Women	2016	10-19	6.05	3.79	9.53	22	370
Piura	Women	2016	10-19	4.87	2.9	8.06	16	434
Puno	Women	2016	10-19	5	3.35	7.41	28	416
San Martin	Women	2016	10-19	5.12	2.83	9.1	14	351
Tacna	Women	2016	10-19	4.93	2.45	9.69	13	428
Tumbes	Women	2016	10-19	4.43	2.1	9.09	9	391
Ucayali	Women	2016	10-19	3.66	1.87	7.04	9	412
Amazonas	Men	2016	20-29	1.06	0.43	2.6	5	381
Ancash	Men	2016	20-29	0.81	0.2	3.19	2	314

Apurimac	Men	2016	20-29	0	0	0	0	330
Arequipa	Men	2016	20-29	1.57	0.57	4.25	4	355
Ayacucho	Men	2016	20-29	0.28	0.06	1.21	2	392
Cajamarca	Men	2016	20-29	1.45	0.31	6.48	3	317
Callao	Men	2016	20-29	1.38	0.39	4.77	3	325
Cusco	Men	2016	20-29	1.39	0.36	5.19	4	365
Huancavelica	Men	2016	20-29	0.17	0.02	1.21	1	298
Huanuco	Men	2016	20-29	0.62	0.13	2.92	2	374
Ica	Men	2016	20-29	0.61	0.17	2.2	3	354
Junin	Men	2016	20-29	1.02	0.21	4.81	2	308
La Libertad	Men	2016	20-29	1.27	0.43	3.66	4	325
Lambayeque	Men	2016	20-29	1.22	0.32	4.55	3	366
Lima	Men	2016	20-29	0.8	0.33	1.92	9	1061
Loreto	Men	2016	20-29	1.01	0.29	3.42	3	378
Madre de Dios	Men	2016	20-29	1.21	0.31	4.63	3	329
Moquegua	Men	2016	20-29	0.71	0.21	2.39	3	337
Pasco	Men	2016	20-29	0.52	0.07	3.51	1	334
Piura	Men	2016	20-29	1.37	0.61	3.05	6	346
Puno	Men	2016	20-29	0.32	0.04	2.27	1	303
San Martin	Men	2016	20-29	0.66	0.2	2.09	3	417
Tacna	Men	2016	20-29	0.45	0.09	2.24	2	362
Tumbes	Men	2016	20-29	0.39	0.05	2.77	1	378
Ucayali	Men	2016	20-29	0.57	0.14	2.31	2	317
Amazonas	Women	2016	20-29	0.32	0.05	2.2	1	421
Ancash	Women	2016	20-29	0.11	0.01	0.78	1	403
Apurimac	Women	2016	20-29	0	0	0	0	356
Arequipa	Women	2016	20-29	0	0	0	0	371
Ayacucho	Women	2016	20-29	0.27	0.04	1.91	1	517
Cajamarca	Women	2016	20-29	0.3	0.04	2.11	1	353

Callao	Women	2016	20-29	0	0	0	0	424
Cusco	Women	2016	20-29	0	0	0	0	363
Huancavelica	Women	2016	20-29	0.68	0.2	2.33	3	395
Huanuco	Women	2016	20-29	0.57	0.08	3.99	1	488
Ica	Women	2016	20-29	0.45	0.06	3.04	1	409
Junin	Women	2016	20-29	0	0	0	0	444
La Libertad	Women	2016	20-29	0.21	0.03	1.45	1	369
Lambayeque	Women	2016	20-29	0.17	0.02	1.25	1	468
Lima	Women	2016	20-29	0.2	0.06	0.66	4	1209
Loreto	Women	2016	20-29	0	0	0	0	369
Madre de Dios	Women	2016	20-29	0.56	0.08	3.71	1	343
Moquegua	Women	2016	20-29	0	0	0	0	411
Pasco	Women	2016	20-29	0.26	0.03	1.84	1	370
Piura	Women	2016	20-29	0	0	0	0	434
Puno	Women	2016	20-29	0.26	0.04	1.83	1	416
San Martin	Women	2016	20-29	0	0	0	0	351
Tacna	Women	2016	20-29	0.16	0.02	1.16	1	428
Tumbes	Women	2016	20-29	0.57	0.08	3.95	1	391
Ucayali	Women	2016	20-29	0	0	0	0	412
Amazonas	Men	2016	30+	0	0	0	0	381
Ancash	Men	2016	30+	0	0	0	0	314
Apurimac	Men	2016	30+	0	0	0	0	330
Arequipa	Men	2016	30+	0	0	0	0	355
Ayacucho	Men	2016	30+	0	0	0	0	392
Cajamarca	Men	2016	30+	0	0	0	0	317
Callao	Men	2016	30+	0	0	0	0	325
Cusco	Men	2016	30+	0	0	0	0	365
Huancavelica	Men	2016	30+	0	0	0	0	298
Huanuco	Men	2016	30+	0	0	0	0	374

Ica	Men	2016	30+	0	0	0	0	354
Junin	Men	2016	30+	0	0	0	0	308
La Libertad	Men	2016	30+	0.34	0.05	2.4	1	325
Lambayeque	Men	2016	30+	0	0	0	0	366
Lima	Men	2016	30+	0.31	0.1	0.95	3	1061
Loreto	Men	2016	30+	0.28	0.04	2.03	1	378
Madre de Dios	Men	2016	30+	0	0	0	0	329
Moquegua	Men	2016	30+	0	0	0	0	337
Pasco	Men	2016	30+	0	0	0	0	334
Piura	Men	2016	30+	0	0	0	0	346
Puno	Men	2016	30+	0.31	0.04	2.25	1	303
San Martin	Men	2016	30+	0	0	0	0	417
Tacna	Men	2016	30+	0.28	0.04	1.95	1	362
Tumbes	Men	2016	30+	0.13	0.02	0.97	1	378
Ucayali	Men	2016	30+	0	0	0	0	317
Amazonas	Women	2016	30+	0	0	0	0	421
Ancash	Women	2016	30+	0	0	0	0	403
Apurimac	Women	2016	30+	0	0	0	0	356
Arequipa	Women	2016	30+	0	0	0	0	371
Ayacucho	Women	2016	30+	0	0	0	0	517
Cajamarca	Women	2016	30+	0	0	0	0	353
Callao	Women	2016	30+	0	0	0	0	424
Cusco	Women	2016	30+	0	0	0	0	363
Huancavelica	Women	2016	30+	0	0	0	0	395
Huanuco	Women	2016	30+	0	0	0	0	488
Ica	Women	2016	30+	0	0	0	0	409
Junin	Women	2016	30+	0	0	0	0	444
La Libertad	Women	2016	30+	0	0	0	0	369
Lambayeque	Women	2016	30+	0	0	0	0	468

Lima	Women	2016	30+	0	0	0	0	1209
Loreto	Women	2016	30+	0	0	0	0	369
Madre de Dios	Women	2016	30+	0	0	0	0	343
Moquegua	Women	2016	30+	0	0	0	0	411
Pasco	Women	2016	30+	0	0	0	0	370
Piura	Women	2016	30+	0	0	0	0	434
Puno	Women	2016	30+	0	0	0	0	416
San Martin	Women	2016	30+	0	0	0	0	351
Tacna	Women	2016	30+	0	0	0	0	428
Tumbes	Women	2016	30+	0	0	0	0	391
Ucayali	Women	2016	30+	0	0	0	0	412
Amazonas	Men	2017	<5	77.05	71.75	81.61	316	396
Ancash	Men	2017	<5	73.03	67.22	78.14	243	330
Apurimac	Men	2017	<5	77.67	71.42	82.88	288	352
Arequipa	Men	2017	<5	76.93	71.8	81.37	282	362
Ayacucho	Men	2017	<5	77.34	72.43	81.59	297	382
Cajamarca	Men	2017	<5	77.28	70.28	83.03	288	352
Callao	Men	2017	<5	71.85	66.1	76.96	255	340
Cusco	Men	2017	<5	78.24	72.28	83.21	263	336
Huancavelica	Men	2017	<5	78.8	74.24	82.74	277	360
Huanuco	Men	2017	<5	77.31	71.38	82.32	312	389
Ica	Men	2017	<5	74.81	70.15	78.96	283	375
Junin	Men	2017	<5	76.49	70.38	81.66	267	337
La Libertad	Men	2017	<5	75.77	70.16	80.62	262	339
Lambayeque	Men	2017	<5	74.44	68.02	79.96	268	348
Lima	Men	2017	<5	74.17	70.63	77.43	827	1069
Loreto	Men	2017	<5	76.6	71.6	80.95	294	386
Madre de Dios	Men	2017	<5	82	77.56	85.72	276	346
Moquegua	Men	2017	<5	71.78	65.93	76.98	277	366

Pasco	Men	2017	<5	81.79	75.88	86.51	291	344
Piura	Men	2017	<5	75.05	70.04	79.46	248	338
Puno	Men	2017	<5	76.1	70.42	80.98	229	328
San Martin	Men	2017	<5	78.68	73.48	83.1	300	373
Tacna	Men	2017	<5	75.51	69.52	80.65	313	384
Tumbes	Men	2017	<5	72.96	66.89	78.27	282	371
Ucayali	Men	2017	<5	82.45	77.15	86.73	216	273
Amazonas	Women	2017	<5	82.31	77.63	86.19	346	414
Ancash	Women	2017	<5	78.57	73.81	82.67	369	453
Apurimac	Women	2017	<5	81.4	76.61	85.4	329	403
Arequipa	Women	2017	<5	83.86	79.92	87.15	354	409
Ayacucho	Women	2017	<5	81.01	77.09	84.41	414	511
Cajamarca	Women	2017	<5	81.16	76.6	85	322	400
Callao	Women	2017	<5	81.06	75.69	85.47	340	406
Cusco	Women	2017	<5	84.47	79.91	88.15	306	365
Huancavelica	Women	2017	<5	80.75	76.41	84.45	342	435
Huanuco	Women	2017	<5	83.96	79.22	87.78	381	451
Ica	Women	2017	<5	80.79	76.37	84.55	390	477
Junin	Women	2017	<5	84.56	80.01	88.23	347	409
La Libertad	Women	2017	<5	81.58	77.53	85.04	334	405
Lambayeque	Women	2017	<5	80.31	76	84	382	456
Lima	Women	2017	<5	81.51	78.09	84.5	1046	1210
Loreto	Women	2017	<5	84.47	80.2	87.96	300	360
Madre de Dios	Women	2017	<5	90.08	84.89	93.62	332	362
Moquegua	Women	2017	<5	82.1	76.78	86.42	366	432
Pasco	Women	2017	<5	84.72	79.41	88.85	298	349
Piura	Women	2017	<5	83.81	78.84	87.79	384	443
Puno	Women	2017	<5	81.44	76.57	85.48	345	431
San Martin	Women	2017	<5	85.28	80.28	89.18	345	387

Tacna	Women	2017	<5	82.11	77.25	86.11	332	390
Tumbes	Women	2017	<5	86.22	80.81	90.29	381	412
Ucayali	Women	2017	<5	88.2	84.33	91.21	397	441
Amazonas	Men	2017	5-9	16.4	12.36	21.43	58	396
Ancash	Men	2017	5-9	17.04	12.89	22.19	51	330
Apurimac	Men	2017	5-9	14.34	10.08	19.99	37	352
Arequipa	Men	2017	5-9	14.13	10.77	18.33	50	362
Ayacucho	Men	2017	5-9	14.61	11.17	18.88	47	382
Cajamarca	Men	2017	5-9	11.31	7.03	17.7	33	352
Callao	Men	2017	5-9	17.12	13.03	22.17	49	340
Cusco	Men	2017	5-9	13.01	9.21	18.07	42	336
Huancavelica	Men	2017	5-9	14.61	11.15	18.9	52	360
Huanuco	Men	2017	5-9	12.71	9	17.65	43	389
Ica	Men	2017	5-9	13.76	10.31	18.14	45	375
Junin	Men	2017	5-9	15.98	11.56	21.67	45	337
La Libertad	Men	2017	5-9	14.16	10.35	19.09	49	339
Lambayeque	Men	2017	5-9	16.24	11.9	21.78	49	348
Lima	Men	2017	5-9	15.31	12.73	18.3	143	1069
Loreto	Men	2017	5-9	14.36	10.93	18.64	61	386
Madre de Dios	Men	2017	5-9	12.72	9.4	17	50	346
Moquegua	Men	2017	5-9	18.89	14.69	23.96	59	366
Pasco	Men	2017	5-9	12.19	8.08	17.98	38	344
Piura	Men	2017	5-9	16.86	12.86	21.8	58	338
Puno	Men	2017	5-9	15.57	11.71	20.42	61	328
San Martin	Men	2017	5-9	12.65	9.19	17.17	43	373
Tacna	Men	2017	5-9	13.37	9.51	18.48	34	384
Tumbes	Men	2017	5-9	18.55	14.51	23.4	64	371
Ucayali	Men	2017	5-9	9.85	6.65	14.34	28	273
Amazonas	Women	2017	5-9	9.2	6.45	12.95	38	414

Ancash	Women	2017	5-9	13.95	10.48	18.33	47	453
Apurimac	Women	2017	5-9	12.67	9.36	16.92	44	403
Arequipa	Women	2017	5-9	11.13	8.24	14.88	38	409
Ayacucho	Women	2017	5-9	12.51	9.54	16.24	64	511
Cajamarca	Women	2017	5-9	12.64	9.72	16.28	55	400
Callao	Women	2017	5-9	13.23	9.83	17.57	48	406
Cusco	Women	2017	5-9	10.43	7.48	14.34	38	365
Huancavelica	Women	2017	5-9	12.83	9.99	16.33	65	435
Huanuco	Women	2017	5-9	12.12	8.5	17	49	451
Ica	Women	2017	5-9	14.77	11.67	18.53	69	477
Junin	Women	2017	5-9	10.96	7.96	14.9	40	409
La Libertad	Women	2017	5-9	12.73	9.6	16.7	49	405
Lambayeque	Women	2017	5-9	14.35	11.09	18.37	54	456
Lima	Women	2017	5-9	11.73	9.46	14.46	112	1210
Loreto	Women	2017	5-9	10.22	7.48	13.83	41	360
Madre de Dios	Women	2017	5-9	6.08	3.9	9.34	22	362
Moquegua	Women	2017	5-9	13.54	9.85	18.33	44	432
Pasco	Women	2017	5-9	9.92	7.01	13.86	33	349
Piura	Women	2017	5-9	11.5	8.61	15.22	43	443
Puno	Women	2017	5-9	13.36	9.94	17.71	62	431
San Martin	Women	2017	5-9	10.11	6.96	14.47	32	387
Tacna	Women	2017	5-9	14.78	11.2	19.26	46	390
Tumbes	Women	2017	5-9	8.92	5.63	13.84	20	412
Ucayali	Women	2017	5-9	7.77	5.21	11.44	30	441
Amazonas	Men	2017	10-19	5.55	3.11	9.72	19	396
Ancash	Men	2017	10-19	9.04	6.2	12.99	34	330
Apurimac	Men	2017	10-19	6.8	4.08	11.12	22	352
Arequipa	Men	2017	10-19	8.4	6	11.63	28	362
Ayacucho	Men	2017	10-19	7.45	5.12	10.74	35	382

Cajamarca	Men	2017	10-19	9.87	6.4	14.92	26	352
Callao	Men	2017	10-19	9.38	6.42	13.52	30	340
Cusco	Men	2017	10-19	8.11	5.1	12.67	28	336
Huancavelica	Men	2017	10-19	5.75	3.83	8.55	27	360
Huanuco	Men	2017	10-19	9.77	6.87	13.72	33	389
Ica	Men	2017	10-19	9.06	6.69	12.16	37	375
Junin	Men	2017	10-19	7.02	4.62	10.53	24	337
La Libertad	Men	2017	10-19	9.41	6.25	13.92	26	339
Lambayeque	Men	2017	10-19	8.76	5.72	13.2	29	348
Lima	Men	2017	10-19	9.74	7.6	12.41	90	1069
Loreto	Men	2017	10-19	9.05	6.3	12.83	31	386
Madre de Dios	Men	2017	10-19	5.28	3.26	8.45	20	346
Moquegua	Men	2017	10-19	8.96	6.13	12.93	29	366
Pasco	Men	2017	10-19	5.9	3.44	9.92	14	344
Piura	Men	2017	10-19	7.59	5.24	10.88	30	338
Puno	Men	2017	10-19	7.52	5.04	11.07	35	328
San Martin	Men	2017	10-19	8.02	5.22	12.12	28	373
Tacna	Men	2017	10-19	9.03	6.1	13.17	29	384
Tumbes	Men	2017	10-19	7.66	5	11.57	23	371
Ucayali	Men	2017	10-19	7.35	4.63	11.46	26	273
Amazonas	Women	2017	10-19	7.78	5.42	11.06	28	414
Ancash	Women	2017	10-19	6.75	4.58	9.83	35	453
Apurimac	Women	2017	10-19	5.82	4.03	8.35	29	403
Arequipa	Women	2017	10-19	5.01	3.08	8.03	17	409
Ayacucho	Women	2017	10-19	6.03	4.31	8.36	31	511
Cajamarca	Women	2017	10-19	5.93	3.72	9.32	22	400
Callao	Women	2017	10-19	5.42	3.27	8.86	17	406
Cusco	Women	2017	10-19	5.1	3.24	7.94	21	365
Huancavelica	Women	2017	10-19	6.09	3.8	9.61	26	435

Huanuco	Women	2017	10-19	3.92	2.42	6.28	21	451
Ica	Women	2017	10-19	4.44	2.7	7.23	18	477
Junin	Women	2017	10-19	4.48	2.74	7.24	22	409
La Libertad	Women	2017	10-19	5.69	3.62	8.83	22	405
Lambayeque	Women	2017	10-19	5.09	3.18	8.06	19	456
Lima	Women	2017	10-19	6.19	4.34	8.74	47	1210
Loreto	Women	2017	10-19	5.31	3.19	8.7	19	360
Madre de Dios	Women	2017	10-19	2.62	1.2	5.61	7	362
Moquegua	Women	2017	10-19	3.91	2.37	6.4	21	432
Pasco	Women	2017	10-19	5.24	2.92	9.23	17	349
Piura	Women	2017	10-19	4.57	2.24	9.1	15	443
Puno	Women	2017	10-19	4.88	3.02	7.78	23	431
San Martin	Women	2017	10-19	4.61	2.37	8.77	10	387
Tacna	Women	2017	10-19	2.87	1.53	5.32	11	390
Tumbes	Women	2017	10-19	4.86	2.58	8.96	11	412
Ucayali	Women	2017	10-19	3.69	2.15	6.27	13	441
Amazonas	Men	2017	20-29	1	0.28	3.46	3	396
Ancash	Men	2017	20-29	0.89	0.17	4.53	2	330
Apurimac	Men	2017	20-29	1.2	0.5	2.85	5	352
Arequipa	Men	2017	20-29	0.54	0.13	2.21	2	362
Ayacucho	Men	2017	20-29	0.6	0.19	1.85	3	382
Cajamarca	Men	2017	20-29	1.54	0.58	3.98	5	352
Callao	Men	2017	20-29	0.95	0.35	2.54	4	340
Cusco	Men	2017	20-29	0.64	0.18	2.21	3	336
Huancavelica	Men	2017	20-29	0.84	0.3	2.33	4	360
Huanuco	Men	2017	20-29	0.21	0.03	1.49	1	389
Ica	Men	2017	20-29	2.12	1.05	4.26	9	375
Junin	Men	2017	20-29	0.52	0.07	3.66	1	337
La Libertad	Men	2017	20-29	0.46	0.06	3.22	1	339

Lambayeque	Men	2017	20-29	0.55	0.13	2.34	2	348
Lima	Men	2017	20-29	0.78	0.31	1.93	9	1069
Loreto	Men	2017	20-29	0	0	0	0	386
Madre de Dios	Men	2017	20-29	0	0	0	0	346
Moquegua	Men	2017	20-29	0	0	0	0	366
Pasco	Men	2017	20-29	0.12	0.02	0.84	1	344
Piura	Men	2017	20-29	0.5	0.13	2	2	338
Puno	Men	2017	20-29	0.81	0.25	2.6	3	328
San Martin	Men	2017	20-29	0.65	0.16	2.65	2	373
Tacna	Men	2017	20-29	2.09	1.05	4.1	8	384
Tumbes	Men	2017	20-29	0.83	0.21	3.33	2	371
Ucayali	Men	2017	20-29	0.36	0.11	1.14	3	273
Amazonas	Women	2017	20-29	0.71	0.18	2.8	2	414
Ancash	Women	2017	20-29	0.73	0.16	3.24	2	453
Apurimac	Women	2017	20-29	0.11	0.02	0.8	1	403
Arequipa	Women	2017	20-29	0	0	0	0	409
Ayacucho	Women	2017	20-29	0.45	0.11	1.79	2	511
Cajamarca	Women	2017	20-29	0.27	0.04	1.97	1	400
Callao	Women	2017	20-29	0.29	0.04	2.06	1	406
Cusco	Women	2017	20-29	0	0	0	0	365
Huancavelica	Women	2017	20-29	0.33	0.07	1.52	2	435
Huanuco	Women	2017	20-29	0	0	0	0	451
Ica	Women	2017	20-29	0	0	0	0	477
Junin	Women	2017	20-29	0	0	0	0	409
La Libertad	Women	2017	20-29	0	0	0	0	405
Lambayeque	Women	2017	20-29	0.25	0.04	1.81	1	456
Lima	Women	2017	20-29	0.57	0.2	1.65	5	1210
Loreto	Women	2017	20-29	0	0	0	0	360
Madre de Dios	Women	2017	20-29	1.23	0.17	8.31	1	362

Moquegua	Women	2017	20-29	0.45	0.06	3.13	1	432
Pasco	Women	2017	20-29	0.12	0.02	0.88	1	349
Piura	Women	2017	20-29	0.12	0.02	0.87	1	443
Puno	Women	2017	20-29	0.33	0.05	2.31	1	431
San Martin	Women	2017	20-29	0	0	0	0	387
Tacna	Women	2017	20-29	0.24	0.03	1.73	1	390
Tumbes	Women	2017	20-29	0	0	0	0	412
Ucayali	Women	2017	20-29	0.34	0.05	2.41	1	441
Amazonas	Men	2017	30+	0	0	0	0	396
Ancash	Men	2017	30+	0	0	0	0	330
Apurimac	Men	2017	30+	0	0	0	0	352
Arequipa	Men	2017	30+	0	0	0	0	362
Ayacucho	Men	2017	30+	0	0	0	0	382
Cajamarca	Men	2017	30+	0	0	0	0	352
Callao	Men	2017	30+	0.7	0.17	2.78	2	340
Cusco	Men	2017	30+	0	0	0	0	336
Huancavelica	Men	2017	30+	0	0	0	0	360
Huanuco	Men	2017	30+	0	0	0	0	389
Ica	Men	2017	30+	0.24	0.03	1.75	1	375
Junin	Men	2017	30+	0	0	0	0	337
La Libertad	Men	2017	30+	0.2	0.03	1.43	1	339
Lambayeque	Men	2017	30+	0	0	0	0	348
Lima	Men	2017	30+	0	0	0	0	1069
Loreto	Men	2017	30+	0	0	0	0	386
Madre de Dios	Men	2017	30+	0	0	0	0	346
Moquegua	Men	2017	30+	0.36	0.05	2.53	1	366
Pasco	Men	2017	30+	0	0	0	0	344
Piura	Men	2017	30+	0	0	0	0	338
Puno	Men	2017	30+	0	0	0	0	328

San Martin	Men	2017	30+	0	0	0	0	373
Tacna	Men	2017	30+	0	0	0	0	384
Tumbes	Men	2017	30+	0	0	0	0	371
Ucayali	Men	2017	30+	0	0	0	0	273
Amazonas	Women	2017	30+	0	0	0	0	414
Ancash	Women	2017	30+	0	0	0	0	453
Apurimac	Women	2017	30+	0	0	0	0	403
Arequipa	Women	2017	30+	0	0	0	0	409
Ayacucho	Women	2017	30+	0	0	0	0	511
Cajamarca	Women	2017	30+	0	0	0	0	400
Callao	Women	2017	30+	0	0	0	0	406
Cusco	Women	2017	30+	0	0	0	0	365
Huancavelica	Women	2017	30+	0	0	0	0	435
Huanuco	Women	2017	30+	0	0	0	0	451
Ica	Women	2017	30+	0	0	0	0	477
Junin	Women	2017	30+	0	0	0	0	409
La Libertad	Women	2017	30+	0	0	0	0	405
Lambayeque	Women	2017	30+	0	0	0	0	456
Lima	Women	2017	30+	0	0	0	0	1210
Loreto	Women	2017	30+	0	0	0	0	360
Madre de Dios	Women	2017	30+	0	0	0	0	362
Moquegua	Women	2017	30+	0	0	0	0	432
Pasco	Women	2017	30+	0	0	0	0	349
Piura	Women	2017	30+	0	0	0	0	443
Puno	Women	2017	30+	0	0	0	0	431
San Martin	Women	2017	30+	0	0	0	0	387
Tacna	Women	2017	30+	0	0	0	0	390
Tumbes	Women	2017	30+	0	0	0	0	412
Ucayali	Women	2017	30+	0	0	0	0	441

Amazonas	Men	2018	<5	80.23	75.36	84.34	375	444
Ancash	Men	2018	<5	71.92	66.34	76.89	270	354
Apurimac	Men	2018	<5	78.7	72.93	83.51	315	381
Arequipa	Men	2018	<5	74.67	69.21	79.45	258	339
Ayacucho	Men	2018	<5	75.9	69.28	81.47	328	397
Cajamarca	Men	2018	<5	76.5	71.05	81.2	307	380
Callao	Men	2018	<5	74.05	69.07	78.47	268	347
Cusco	Men	2018	<5	75.83	68.62	81.82	282	354
Huancavelica	Men	2018	<5	75.35	69.82	80.16	315	395
Huanuco	Men	2018	<5	79.62	75.29	83.35	313	393
Ica	Men	2018	<5	72.16	67.34	76.52	268	354
Junin	Men	2018	<5	77.7	71.13	83.13	236	296
La Libertad	Men	2018	<5	75.73	70.07	80.62	262	330
Lambayeque	Men	2018	<5	70.02	63.71	75.65	298	395
Lima	Men	2018	<5	72.33	69.19	75.26	973	1263
Loreto	Men	2018	<5	74.58	69.22	79.29	283	374
Madre de Dios	Men	2018	<5	78.39	72.94	83.01	272	352
Moquegua	Men	2018	<5	73.56	68	78.46	295	383
Pasco	Men	2018	<5	78.01	72.18	82.91	276	358
Piura	Men	2018	<5	73.01	67.27	78.08	289	369
Puno	Men	2018	<5	74.1	68.92	78.69	263	374
San Martin	Men	2018	<5	77.95	73.22	82.04	349	413
Tacna	Men	2018	<5	76.07	71.03	80.48	299	380
Tumbes	Men	2018	<5	75.78	69.6	81.06	337	411
Ucayali	Men	2018	<5	79.18	73.96	83.58	244	309
Amazonas	Women	2018	<5	83.35	78.25	87.44	398	454
Ancash	Women	2018	<5	76.9	71.81	81.32	393	471
Apurimac	Women	2018	<5	82.38	77.79	86.19	376	436
Arequipa	Women	2018	<5	83.33	79.24	86.75	392	452

Ayacucho	Women	2018	<5	81.95	77.62	85.59	442	532
Cajamarca	Women	2018	<5	82.5	78.32	86.02	405	482
Callao	Women	2018	<5	80.24	75.94	83.94	352	422
Cusco	Women	2018	<5	81.61	75.81	86.27	357	415
Huancavelica	Women	2018	<5	79.9	75.93	83.35	368	461
Huanuco	Women	2018	<5	84.08	80.18	87.33	410	484
Ica	Women	2018	<5	82.67	78.68	86.05	410	477
Junin	Women	2018	<5	82.41	77.22	86.62	399	454
La Libertad	Women	2018	<5	81.51	77.86	84.68	384	451
Lambayeque	Women	2018	<5	81.26	77.23	84.73	392	473
Lima	Women	2018	<5	79.78	77.08	82.23	1232	1474
Loreto	Women	2018	<5	84.09	79.72	87.66	328	380
Madre de Dios	Women	2018	<5	90.02	85.72	93.13	317	346
Moquegua	Women	2018	<5	82.25	76.8	86.65	384	443
Pasco	Women	2018	<5	81.79	76.28	86.25	303	367
Piura	Women	2018	<5	82.58	77.76	86.54	401	463
Puno	Women	2018	<5	79.78	74.51	84.19	353	454
San Martin	Women	2018	<5	84.19	78.93	88.33	328	370
Tacna	Women	2018	<5	85.61	80.16	89.76	422	472
Tumbes	Women	2018	<5	85.91	81.42	89.46	343	388
Ucayali	Women	2018	<5	85.24	81.14	88.57	406	471
Amazonas	Men	2018	5-9	10.77	7.37	15.48	36	444
Ancash	Men	2018	5-9	17.61	13.5	22.65	49	354
Apurimac	Men	2018	5-9	13.14	9.58	17.75	43	381
Arequipa	Men	2018	5-9	13.9	10.53	18.13	45	339
Ayacucho	Men	2018	5-9	14.26	10.24	19.51	45	397
Cajamarca	Men	2018	5-9	14.98	11.04	19.99	45	380
Callao	Men	2018	5-9	13.82	10.56	17.88	47	347
Cusco	Men	2018	5-9	16.22	11.34	22.68	47	354

Huancavelica	Men	2018	5-9	16.38	11.88	22.15	52	395
Huanuco	Men	2018	5-9	12.02	8.85	16.12	45	393
Ica	Men	2018	5-9	15.46	11.59	20.33	45	354
Junin	Men	2018	5-9	13.15	9.32	18.24	38	296
La Libertad	Men	2018	5-9	15.56	11.62	20.52	42	330
Lambayeque	Men	2018	5-9	19.52	15.15	24.78	69	395
Lima	Men	2018	5-9	16.85	14.46	19.54	166	1263
Loreto	Men	2018	5-9	18.11	14.1	22.97	60	374
Madre de Dios	Men	2018	5-9	13.63	10.06	18.2	48	352
Moquegua	Men	2018	5-9	15.62	11.91	20.2	50	383
Pasco	Men	2018	5-9	14.12	10.33	19	46	358
Piura	Men	2018	5-9	16.48	12.94	20.76	53	369
Puno	Men	2018	5-9	18.79	14.74	23.63	76	374
San Martin	Men	2018	5-9	13.3	10.08	17.34	41	413
Tacna	Men	2018	5-9	14.28	10.75	18.72	48	380
Tumbes	Men	2018	5-9	17.34	13.56	21.91	57	411
Ucayali	Men	2018	5-9	14.31	10.67	18.92	47	309
Amazonas	Women	2018	5-9	10.36	7.37	14.39	36	454
Ancash	Women	2018	5-9	15.48	11.89	19.91	55	471
Apurimac	Women	2018	5-9	10.46	7.5	14.42	39	436
Arequipa	Women	2018	5-9	12.78	9.73	16.61	45	452
Ayacucho	Women	2018	5-9	12.7	9.48	16.82	64	532
Cajamarca	Women	2018	5-9	11.68	8.77	15.39	51	482
Callao	Women	2018	5-9	12.53	9.51	16.34	44	422
Cusco	Women	2018	5-9	11.67	8.37	16.04	40	415
Huancavelica	Women	2018	5-9	14.99	11.94	18.66	66	461
Huanuco	Women	2018	5-9	12.15	9.17	15.93	55	484
Ica	Women	2018	5-9	13.46	10.53	17.05	56	477
Junin	Women	2018	5-9	11.94	8.43	16.64	39	454

La Libertad	Women	2018	5-9	12.92	9.95	16.61	45	451
Lambayeque	Women	2018	5-9	11.76	9.07	15.11	53	473
Lima	Women	2018	5-9	13.42	11.48	15.63	169	1474
Loreto	Women	2018	5-9	10.58	7.76	14.27	36	380
Madre de Dios	Women	2018	5-9	8.22	5.36	12.41	23	346
Moquegua	Women	2018	5-9	14.26	10.29	19.44	45	443
Pasco	Women	2018	5-9	13.86	10.38	18.28	51	367
Piura	Women	2018	5-9	13.53	10.34	17.51	52	463
Puno	Women	2018	5-9	13.69	9.81	18.79	69	454
San Martin	Women	2018	5-9	10.34	7.04	14.93	28	370
Tacna	Women	2018	5-9	10.52	7.67	14.27	41	472
Tumbes	Women	2018	5-9	10.63	7.55	14.75	34	388
Ucayali	Women	2018	5-9	9.65	6.98	13.19	47	471
Amazonas	Men	2018	10-19	8.15	5.51	11.91	29	444
Ancash	Men	2018	10-19	8.12	5.3	12.25	29	354
Apurimac	Men	2018	10-19	7.25	4.5	11.48	21	381
Arequipa	Men	2018	10-19	10.46	7.17	15	33	339
Ayacucho	Men	2018	10-19	8.55	5.17	13.84	20	397
Cajamarca	Men	2018	10-19	7.16	4.58	11.03	24	380
Callao	Men	2018	10-19	11.54	8.14	16.1	31	347
Cusco	Men	2018	10-19	7.24	4.08	12.55	23	354
Huancavelica	Men	2018	10-19	7.72	5.37	10.99	27	395
Huanuco	Men	2018	10-19	7.66	5.24	11.07	33	393
Ica	Men	2018	10-19	11.19	8.22	15.06	38	354
Junin	Men	2018	10-19	8.86	5.42	14.16	21	296
La Libertad	Men	2018	10-19	7.27	4.83	10.79	21	330
Lambayeque	Men	2018	10-19	9.86	6.65	14.39	26	395
Lima	Men	2018	10-19	9.51	7.67	11.74	109	1263
Loreto	Men	2018	10-19	6.49	4.3	9.69	28	374

Madre de Dios	Men	2018	10-19	7.87	5.48	11.18	31	352
Moquegua	Men	2018	10-19	9.92	6.65	14.55	35	383
Pasco	Men	2018	10-19	7.87	5.53	11.07	36	358
Piura	Men	2018	10-19	9.85	6.49	14.67	26	369
Puno	Men	2018	10-19	6.5	4.01	10.38	31	374
San Martin	Men	2018	10-19	8.17	5.4	12.18	21	413
Tacna	Men	2018	10-19	8.12	5.22	12.41	29	380
Tumbes	Men	2018	10-19	5.16	2.82	9.25	14	411
Ucayali	Men	2018	10-19	5.74	3.53	9.21	16	309
Amazonas	Women	2018	10-19	5.8	3.57	9.27	19	454
Ancash	Women	2018	10-19	7.07	4.33	11.36	20	471
Apurimac	Women	2018	10-19	6.99	4.39	10.95	20	436
Arequipa	Women	2018	10-19	3.89	2.3	6.5	15	452
Ayacucho	Women	2018	10-19	5.35	3.5	8.1	26	532
Cajamarca	Women	2018	10-19	5.43	3.61	8.09	24	482
Callao	Women	2018	10-19	6.88	4.59	10.19	25	422
Cusco	Women	2018	10-19	6.72	3.72	11.84	18	415
Huancavelica	Women	2018	10-19	4.93	3.3	7.31	26	461
Huanuco	Women	2018	10-19	3.78	2.31	6.11	19	484
Ica	Women	2018	10-19	3.86	2.13	6.92	11	477
Junin	Women	2018	10-19	5.66	3.36	9.38	16	454
La Libertad	Women	2018	10-19	4.09	2.38	6.95	17	451
Lambayeque	Women	2018	10-19	6.85	4.47	10.36	27	473
Lima	Women	2018	10-19	6.8	5.23	8.81	73	1474
Loreto	Women	2018	10-19	5.33	3.18	8.8	16	380
Madre de Dios	Women	2018	10-19	1.76	0.66	4.6	6	346
Moquegua	Women	2018	10-19	3.48	1.66	7.17	14	443
Pasco	Women	2018	10-19	4.35	2.06	8.96	13	367
Piura	Women	2018	10-19	3.39	1.58	7.12	9	463

Puno	Women	2018	10-19	6.36	4.18	9.55	31	454
San Martin	Women	2018	10-19	5.3	2.9	9.49	13	370
Tacna	Women	2018	10-19	3.87	1.65	8.83	9	472
Tumbes	Women	2018	10-19	3.2	1.55	6.52	10	388
Ucayali	Women	2018	10-19	5.12	3.18	8.14	18	471
Amazonas	Men	2018	20-29	0.84	0.31	2.3	4	444
Ancash	Men	2018	20-29	1.7	0.6	4.76	5	354
Apurimac	Men	2018	20-29	0.92	0.22	3.67	2	381
Arequipa	Men	2018	20-29	0.98	0.3	3.14	3	339
Ayacucho	Men	2018	20-29	1.29	0.46	3.54	4	397
Cajamarca	Men	2018	20-29	1.36	0.47	3.86	4	380
Callao	Men	2018	20-29	0	0	0	0	347
Cusco	Men	2018	20-29	0.71	0.16	2.96	2	354
Huancavelica	Men	2018	20-29	0.55	0.08	3.84	1	395
Huanuco	Men	2018	20-29	0.7	0.13	3.6	2	393
Ica	Men	2018	20-29	1.18	0.35	3.96	3	354
Junin	Men	2018	20-29	0.29	0.04	2.11	1	296
La Libertad	Men	2018	20-29	1.13	0.42	2.99	4	330
Lambayeque	Men	2018	20-29	0.6	0.15	2.44	2	395
Lima	Men	2018	20-29	1.2	0.65	2.19	14	1263
Loreto	Men	2018	20-29	0.55	0.14	2.17	2	374
Madre de Dios	Men	2018	20-29	0.11	0.02	0.8	1	352
Moquegua	Men	2018	20-29	0.9	0.29	2.77	3	383
Pasco	Men	2018	20-29	0	0	0	0	358
Piura	Men	2018	20-29	0.66	0.09	4.64	1	369
Puno	Men	2018	20-29	0.61	0.22	1.71	4	374
San Martin	Men	2018	20-29	0.58	0.14	2.37	2	413
Tacna	Men	2018	20-29	1.54	0.52	4.49	4	380
Tumbes	Men	2018	20-29	1.72	0.41	6.89	3	411

Ucayali	Men	2018	20-29	0.77	0.17	3.37	2	309
Amazonas	Women	2018	20-29	0	0	0	0	454
Ancash	Women	2018	20-29	0.54	0.17	1.69	3	471
Apurimac	Women	2018	20-29	0.17	0.02	1.21	1	436
Arequipa	Women	2018	20-29	0	0	0	0	452
Ayacucho	Women	2018	20-29	0	0	0	0	532
Cajamarca	Women	2018	20-29	0.39	0.1	1.58	2	482
Callao	Women	2018	20-29	0.35	0.05	2.41	1	422
Cusco	Women	2018	20-29	0	0	0	0	415
Huancavelica	Women	2018	20-29	0.18	0.02	1.33	1	461
Huanuco	Women	2018	20-29	0	0	0	0	484
Ica	Women	2018	20-29	0	0	0	0	477
Junin	Women	2018	20-29	0	0	0	0	454
La Libertad	Women	2018	20-29	1.39	0.47	4.08	4	451
Lambayeque	Women	2018	20-29	0.13	0.02	0.92	1	473
Lima	Women	2018	20-29	0	0	0	0	1474
Loreto	Women	2018	20-29	0	0	0	0	380
Madre de Dios	Women	2018	20-29	0	0	0	0	346
Moquegua	Women	2018	20-29	0	0	0	0	443
Pasco	Women	2018	20-29	0	0	0	0	367
Piura	Women	2018	20-29	0.5	0.07	3.6	1	463
Puno	Women	2018	20-29	0.17	0.02	1.24	1	454
San Martin	Women	2018	20-29	0.17	0.02	1.24	1	370
Tacna	Women	2018	20-29	0	0	0	0	472
Tumbes	Women	2018	20-29	0.26	0.04	1.86	1	388
Ucayali	Women	2018	20-29	0	0	0	0	471
Amazonas	Men	2018	30+	0	0	0	0	444
Ancash	Men	2018	30+	0.65	0.09	4.47	1	354
Apurimac	Men	2018	30+	0	0	0	0	381

Arequipa	Men	2018	30+	0	0	0	0	339
Ayacucho	Men	2018	30+	0	0	0	0	397
Cajamarca	Men	2018	30+	0	0	0	0	380
Callao	Men	2018	30+	0.6	0.08	4.16	1	347
Cusco	Men	2018	30+	0	0	0	0	354
Huancavelica	Men	2018	30+	0	0	0	0	395
Huanuco	Men	2018	30+	0	0	0	0	393
Ica	Men	2018	30+	0	0	0	0	354
Junin	Men	2018	30+	0	0	0	0	296
La Libertad	Men	2018	30+	0.32	0.04	2.29	1	330
Lambayeque	Men	2018	30+	0	0	0	0	395
Lima	Men	2018	30+	0.11	0.02	0.79	1	1263
Loreto	Men	2018	30+	0.27	0.04	1.92	1	374
Madre de Dios	Men	2018	30+	0	0	0	0	352
Moquegua	Men	2018	30+	0	0	0	0	383
Pasco	Men	2018	30+	0	0	0	0	358
Piura	Men	2018	30+	0	0	0	0	369
Puno	Men	2018	30+	0	0	0	0	374
San Martin	Men	2018	30+	0	0	0	0	413
Tacna	Men	2018	30+	0	0	0	0	380
Tumbes	Men	2018	30+	0	0	0	0	411
Ucayali	Men	2018	30+	0	0	0	0	309
Amazonas	Women	2018	30+	0.49	0.07	3.43	1	454
Ancash	Women	2018	30+	0	0	0	0	471
Apurimac	Women	2018	30+	0	0	0	0	436
Arequipa	Women	2018	30+	0	0	0	0	452
Ayacucho	Women	2018	30+	0	0	0	0	532
Cajamarca	Women	2018	30+	0	0	0	0	482
Callao	Women	2018	30+	0	0	0	0	422

Cusco	Women	2018	30+	0	0	0	0	415
Huancavelica	Women	2018	30+	0	0	0	0	461
Huanuco	Women	2018	30+	0	0	0	0	484
Ica	Women	2018	30+	0	0	0	0	477
Junin	Women	2018	30+	0	0	0	0	454
La Libertad	Women	2018	30+	0.09	0.01	0.63	1	451
Lambayeque	Women	2018	30+	0	0	0	0	473
Lima	Women	2018	30+	0	0	0	0	1474
Loreto	Women	2018	30+	0	0	0	0	380
Madre de Dios	Women	2018	30+	0	0	0	0	346
Moquegua	Women	2018	30+	0	0	0	0	443
Pasco	Women	2018	30+	0	0	0	0	367
Piura	Women	2018	30+	0	0	0	0	463
Puno	Women	2018	30+	0	0	0	0	454
San Martin	Women	2018	30+	0	0	0	0	370
Tacna	Women	2018	30+	0	0	0	0	472
Tumbes	Women	2018	30+	0	0	0	0	388
Ucayali	Women	2018	30+	0	0	0	0	471
Amazonas	Men	2019	<5	77.85	73.07	81.98	301	384
Ancash	Men	2019	<5	72.42	66.85	77.37	228	320
Apurimac	Men	2019	<5	76.57	71.91	80.67	293	376
Arequipa	Men	2019	<5	73.04	66.04	79.06	280	349
Ayacucho	Men	2019	<5	77.11	71.8	81.68	315	422
Cajamarca	Men	2019	<5	79.4	74.25	83.75	292	361
Callao	Men	2019	<5	71.49	65.33	76.95	242	324
Cusco	Men	2019	<5	80.8	75.51	85.17	291	363
Huancavelica	Men	2019	<5	75.29	69.36	80.41	275	378
Huanuco	Men	2019	<5	76.09	70.25	81.09	310	395
Ica	Men	2019	<5	75.84	71.07	80.04	254	335

Junin	Men	2019	<5	76.97	71.48	81.66	244	307
La Libertad	Men	2019	<5	73.2	66.93	78.67	237	315
Lambayeque	Men	2019	<5	71.97	66.19	77.1	246	331
Lima	Men	2019	<5	74.84	71.48	77.92	916	1172
Loreto	Men	2019	<5	75.71	70.82	80.02	284	368
Madre de Dios	Men	2019	<5	79.37	74.85	83.25	287	356
Moquegua	Men	2019	<5	72.99	66.45	78.66	282	375
Pasco	Men	2019	<5	77.43	71.8	82.21	306	373
Piura	Men	2019	<5	72.16	65.61	77.89	281	356
Puno	Men	2019	<5	76.01	70.17	81.01	244	337
San Martin	Men	2019	<5	79.35	73.74	84.01	336	401
Tacna	Men	2019	<5	76.21	70.8	80.89	278	366
Tumbes	Men	2019	<5	73	67.33	78.01	271	348
Ucayali	Men	2019	<5	80.37	75.63	84.39	260	319
Amazonas	Women	2019	<5	80.92	76.86	84.41	370	453
Ancash	Women	2019	<5	79.6	74.55	83.87	410	495
Apurimac	Women	2019	<5	80.8	75.04	85.49	346	421
Arequipa	Women	2019	<5	82.79	78.16	86.6	357	410
Ayacucho	Women	2019	<5	80.9	76.43	84.69	428	526
Cajamarca	Women	2019	<5	82.42	78.12	86.03	393	474
Callao	Women	2019	<5	80.62	76.11	84.46	345	414
Cusco	Women	2019	<5	84.74	79.79	88.65	352	417
Huancavelica	Women	2019	<5	78.96	74.07	83.14	350	472
Huanuco	Women	2019	<5	84.78	79.63	88.8	410	475
Ica	Women	2019	<5	82.71	77.93	86.63	378	437
Junin	Women	2019	<5	81.47	76.47	85.61	408	465
La Libertad	Women	2019	<5	80.29	75.67	84.21	346	418
Lambayeque	Women	2019	<5	80.95	76.6	84.66	369	435
Lima	Women	2019	<5	81.5	78.96	83.8	1138	1361

Loreto	Women	2019	<5	83.68	78.81	87.61	311	361
Madre de Dios	Women	2019	<5	89.8	84.81	93.28	312	341
Moquegua	Women	2019	<5	82.13	77.78	85.79	388	449
Pasco	Women	2019	<5	83.61	78.08	87.96	314	372
Piura	Women	2019	<5	81.68	77.14	85.48	341	409
Puno	Women	2019	<5	78.65	73.52	83.01	375	492
San Martin	Women	2019	<5	82.89	77.85	86.98	328	385
Tacna	Women	2019	<5	82.93	78.42	86.66	385	454
Tumbes	Women	2019	<5	87.75	83.56	90.99	352	396
Ucayali	Women	2019	<5	84.9	80.48	88.46	383	435
Amazonas	Men	2019	5-9	14.5	11.08	18.75	53	384
Ancash	Men	2019	5-9	16.61	12.51	21.73	53	320
Apurimac	Men	2019	5-9	16.36	13.26	20.03	50	376
Arequipa	Men	2019	5-9	16.65	11.7	23.14	40	349
Ayacucho	Men	2019	5-9	13.42	10.13	17.57	58	422
Cajamarca	Men	2019	5-9	10.94	7.64	15.41	36	361
Callao	Men	2019	5-9	15.78	11.67	20.99	47	324
Cusco	Men	2019	5-9	11.83	8.84	15.65	47	363
Huancavelica	Men	2019	5-9	14.34	9.98	20.18	51	378
Huanuco	Men	2019	5-9	14.92	11.04	19.86	55	395
Ica	Men	2019	5-9	12.95	9.51	17.4	39	335
Junin	Men	2019	5-9	14.77	11.07	19.43	43	307
La Libertad	Men	2019	5-9	15.1	10.87	20.58	43	315
Lambayeque	Men	2019	5-9	15.99	11.86	21.21	49	331
Lima	Men	2019	5-9	14.08	11.84	16.66	139	1172
Loreto	Men	2019	5-9	18.94	15.06	23.53	62	368
Madre de Dios	Men	2019	5-9	16.38	12.81	20.72	54	356
Moquegua	Men	2019	5-9	17.05	12.94	22.12	62	375
Pasco	Men	2019	5-9	14.78	10.77	19.94	44	373

Piura	Men	2019	5-9	19.54	14.76	25.42	52	356
Puno	Men	2019	5-9	14.76	10.22	20.85	55	337
San Martin	Men	2019	5-9	11.95	7.86	17.75	39	401
Tacna	Men	2019	5-9	14.85	11.16	19.49	58	366
Tumbes	Men	2019	5-9	17.3	13.47	21.94	50	348
Ucayali	Men	2019	5-9	14.43	10.85	18.93	45	319
Amazonas	Women	2019	5-9	13.21	10.31	16.77	57	453
Ancash	Women	2019	5-9	13.93	10.47	18.29	61	495
Apurimac	Women	2019	5-9	12.46	9.27	16.54	51	421
Arequipa	Women	2019	5-9	11.92	8.72	16.08	37	410
Ayacucho	Women	2019	5-9	13.67	10.24	18.02	69	526
Cajamarca	Women	2019	5-9	11.27	8.43	14.91	48	474
Callao	Women	2019	5-9	11.65	8.46	15.85	44	414
Cusco	Women	2019	5-9	11.55	8.62	15.3	50	417
Huancavelica	Women	2019	5-9	15.48	12.25	19.37	88	472
Huanuco	Women	2019	5-9	10.8	7.38	15.54	49	475
Ica	Women	2019	5-9	13.58	10.24	17.78	49	437
Junin	Women	2019	5-9	13.3	9.96	17.55	43	465
La Libertad	Women	2019	5-9	14.53	10.97	19.01	52	418
Lambayeque	Women	2019	5-9	12.68	9.98	15.98	43	435
Lima	Women	2019	5-9	12.15	10.14	14.49	154	1361
Loreto	Women	2019	5-9	12.49	9.12	16.88	37	361
Madre de Dios	Women	2019	5-9	9.09	5.72	14.14	24	341
Moquegua	Women	2019	5-9	11.72	8.77	15.49	40	449
Pasco	Women	2019	5-9	12	8.42	16.82	44	372
Piura	Women	2019	5-9	12.22	9.12	16.2	48	409
Puno	Women	2019	5-9	15.98	12.21	20.64	80	492
San Martin	Women	2019	5-9	11.84	9.01	15.42	43	385
Tacna	Women	2019	5-9	12.86	9.64	16.96	50	454

Tumbes	Women	2019	5-9	8.86	6.07	12.76	32	396
Ucayali	Women	2019	5-9	10.96	7.73	15.32	38	435
Amazonas	Men	2019	10-19	6.06	4	9.06	24	384
Ancash	Men	2019	10-19	10.58	7.28	15.12	38	320
Apurimac	Men	2019	10-19	6.77	4.44	10.2	32	376
Arequipa	Men	2019	10-19	9.84	6.56	14.52	27	349
Ayacucho	Men	2019	10-19	8.72	6.06	12.4	45	422
Cajamarca	Men	2019	10-19	8.01	5.32	11.91	28	361
Callao	Men	2019	10-19	11.4	7.77	16.43	32	324
Cusco	Men	2019	10-19	6.15	3.65	10.16	22	363
Huancavelica	Men	2019	10-19	9.92	7.31	13.34	50	378
Huanuco	Men	2019	10-19	7.98	4.58	13.54	27	395
Ica	Men	2019	10-19	8.96	6.17	12.84	35	335
Junin	Men	2019	10-19	8.27	5.01	13.34	20	307
La Libertad	Men	2019	10-19	10.76	7.22	15.74	32	315
Lambayeque	Men	2019	10-19	11.76	8.67	15.75	35	331
Lima	Men	2019	10-19	10.18	8.21	12.55	104	1172
Loreto	Men	2019	10-19	4.9	3.06	7.74	20	368
Madre de Dios	Men	2019	10-19	3.46	1.92	6.14	12	356
Moquegua	Men	2019	10-19	8.36	5.12	13.38	27	375
Pasco	Men	2019	10-19	7.79	4.89	12.21	23	373
Piura	Men	2019	10-19	6.53	4.01	10.47	18	356
Puno	Men	2019	10-19	8.77	6.05	12.54	35	337
San Martin	Men	2019	10-19	7.62	4.75	12	22	401
Tacna	Men	2019	10-19	7.95	5.2	11.95	29	366
Tumbes	Men	2019	10-19	8.88	5.99	12.98	26	348
Ucayali	Men	2019	10-19	5.2	3	8.85	14	319
Amazonas	Women	2019	10-19	5.87	3.76	9.07	26	453
Ancash	Women	2019	10-19	6.47	3.91	10.53	24	495

Apurimac	Women	2019	10-19	6.74	4.12	10.83	24	421
Arequipa	Women	2019	10-19	5.29	3.27	8.45	16	410
Ayacucho	Women	2019	10-19	5.43	3.53	8.26	29	526
Cajamarca	Women	2019	10-19	5.96	4.01	8.79	31	474
Callao	Women	2019	10-19	7.08	4.9	10.12	23	414
Cusco	Women	2019	10-19	3.72	1.92	7.08	15	417
Huancavelica	Women	2019	10-19	5.35	3.33	8.47	32	472
Huanuco	Women	2019	10-19	4.43	2.45	7.87	16	475
Ica	Women	2019	10-19	3.18	1.55	6.4	9	437
Junin	Women	2019	10-19	5.22	2.99	8.96	14	465
La Libertad	Women	2019	10-19	4.83	3.07	7.52	18	418
Lambayeque	Women	2019	10-19	5.94	3.96	8.82	22	435
Lima	Women	2019	10-19	5.71	4.39	7.4	63	1361
Loreto	Women	2019	10-19	3.07	1.6	5.79	10	361
Madre de Dios	Women	2019	10-19	1.11	0.42	2.92	5	341
Moquegua	Women	2019	10-19	6.15	3.79	9.81	21	449
Pasco	Women	2019	10-19	4.31	2.16	8.42	13	372
Piura	Women	2019	10-19	5.77	3.7	8.91	19	409
Puno	Women	2019	10-19	4.99	3.28	7.54	36	492
San Martin	Women	2019	10-19	5.26	2.99	9.11	14	385
Tacna	Women	2019	10-19	4.2	2.59	6.75	19	454
Tumbes	Women	2019	10-19	3.39	1.88	6.01	12	396
Ucayali	Women	2019	10-19	3.58	2.09	6.07	13	435
Amazonas	Men	2019	20-29	1.6	0.7	3.64	6	384
Ancash	Men	2019	20-29	0.38	0.05	2.72	1	320
Apurimac	Men	2019	20-29	0.29	0.04	2.08	1	376
Arequipa	Men	2019	20-29	0.46	0.11	1.96	2	349
Ayacucho	Men	2019	20-29	0.75	0.25	2.17	4	422
Cajamarca	Men	2019	20-29	0.86	0.27	2.69	3	361

Callao	Men	2019	20-29	1.33	0.4	4.29	3	324
Cusco	Men	2019	20-29	1.22	0.38	3.83	3	363
Huancavelica	Men	2019	20-29	0.44	0.11	1.79	2	378
Huanuco	Men	2019	20-29	0.69	0.17	2.81	2	395
Ica	Men	2019	20-29	2.24	1.01	4.92	7	335
Junin	Men	2019	20-29	0	0	0	0	307
La Libertad	Men	2019	20-29	0.94	0.3	2.94	3	315
Lambayeque	Men	2019	20-29	0.29	0.04	2.08	1	331
Lima	Men	2019	20-29	0.88	0.43	1.77	11	1172
Loreto	Men	2019	20-29	0.45	0.11	1.8	2	368
Madre de Dios	Men	2019	20-29	0.79	0.25	2.53	3	356
Moquegua	Men	2019	20-29	1.6	0.57	4.43	4	375
Pasco	Men	2019	20-29	0	0	0	0	373
Piura	Men	2019	20-29	1.77	0.68	4.53	5	356
Puno	Men	2019	20-29	0.47	0.13	1.61	3	337
San Martin	Men	2019	20-29	0.85	0.27	2.63	3	401
Tacna	Men	2019	20-29	0.99	0.14	6.78	1	366
Tumbes	Men	2019	20-29	0.82	0.11	5.67	1	348
Ucayali	Men	2019	20-29	0	0	0	0	319
Amazonas	Women	2019	20-29	0	0	0	0	453
Ancash	Women	2019	20-29	0	0	0	0	495
Apurimac	Women	2019	20-29	0	0	0	0	421
Arequipa	Women	2019	20-29	0	0	0	0	410
Ayacucho	Women	2019	20-29	0	0	0	0	526
Cajamarca	Women	2019	20-29	0.35	0.08	1.5	2	474
Callao	Women	2019	20-29	0.65	0.16	2.57	2	414
Cusco	Women	2019	20-29	0	0	0	0	417
Huancavelica	Women	2019	20-29	0.21	0.05	0.91	2	472
Huanuco	Women	2019	20-29	0	0	0	0	475

Ica	Women	2019	20-29	0.54	0.07	3.76	1	437
Junin	Women	2019	20-29	0	0	0	0	465
La Libertad	Women	2019	20-29	0.35	0.08	1.55	2	418
Lambayeque	Women	2019	20-29	0.43	0.06	3.04	1	435
Lima	Women	2019	20-29	0.64	0.25	1.63	6	1361
Loreto	Women	2019	20-29	0.76	0.22	2.58	3	361
Madre de Dios	Women	2019	20-29	0	0	0	0	341
Moquegua	Women	2019	20-29	0	0	0	0	449
Pasco	Women	2019	20-29	0.08	0.01	0.59	1	372
Piura	Women	2019	20-29	0.33	0.04	2.31	1	409
Puno	Women	2019	20-29	0.37	0.05	2.83	1	492
San Martin	Women	2019	20-29	0	0	0	0	385
Tacna	Women	2019	20-29	0	0	0	0	454
Tumbes	Women	2019	20-29	0	0	0	0	396
Ucayali	Women	2019	20-29	0.56	0.08	4	1	435
Amazonas	Men	2019	30+	0	0	0	0	384
Ancash	Men	2019	30+	0	0	0	0	320
Apurimac	Men	2019	30+	0	0	0	0	376
Arequipa	Men	2019	30+	0	0	0	0	349
Ayacucho	Men	2019	30+	0	0	0	0	422
Cajamarca	Men	2019	30+	0.79	0.17	3.66	2	361
Callao	Men	2019	30+	0	0	0	0	324
Cusco	Men	2019	30+	0	0	0	0	363
Huancavelica	Men	2019	30+	0	0	0	0	378
Huanuco	Men	2019	30+	0.32	0.04	2.34	1	395
Ica	Men	2019	30+	0	0	0	0	335
Junin	Men	2019	30+	0	0	0	0	307
La Libertad	Men	2019	30+	0	0	0	0	315
Lambayeque	Men	2019	30+	0	0	0	0	331

Lima	Men	2019	30+	0.03	0.01	0.11	2	1172
Loreto	Men	2019	30+	0	0	0	0	368
Madre de Dios	Men	2019	30+	0	0	0	0	356
Moquegua	Men	2019	30+	0	0	0	0	375
Pasco	Men	2019	30+	0	0	0	0	373
Piura	Men	2019	30+	0	0	0	0	356
Puno	Men	2019	30+	0	0	0	0	337
San Martin	Men	2019	30+	0.23	0.03	1.66	1	401
Tacna	Men	2019	30+	0	0	0	0	366
Tumbes	Men	2019	30+	0	0	0	0	348
Ucayali	Men	2019	30+	0	0	0	0	319
Amazonas	Women	2019	30+	0	0	0	0	453
Ancash	Women	2019	30+	0	0	0	0	495
Apurimac	Women	2019	30+	0	0	0	0	421
Arequipa	Women	2019	30+	0	0	0	0	410
Ayacucho	Women	2019	30+	0	0	0	0	526
Cajamarca	Women	2019	30+	0	0	0	0	474
Callao	Women	2019	30+	0	0	0	0	414
Cusco	Women	2019	30+	0	0	0	0	417
Huancavelica	Women	2019	30+	0	0	0	0	472
Huanuco	Women	2019	30+	0	0	0	0	475
Ica	Women	2019	30+	0	0	0	0	437
Junin	Women	2019	30+	0	0	0	0	465
La Libertad	Women	2019	30+	0	0	0	0	418
Lambayeque	Women	2019	30+	0	0	0	0	435
Lima	Women	2019	30+	0	0	0	0	1361
Loreto	Women	2019	30+	0	0	0	0	361
Madre de Dios	Women	2019	30+	0	0	0	0	341
Moquegua	Women	2019	30+	0	0	0	0	449

Pasco	Women	2019	30+	0	0	0	0	372
Piura	Women	2019	30+	0	0	0	0	409
Puno	Women	2019	30+	0	0	0	0	492
San Martin	Women	2019	30+	0	0	0	0	385
Tacna	Women	2019	30+	0	0	0	0	454
Tumbes	Women	2019	30+	0	0	0	0	396
Ucayali	Women	2019	30+	0	0	0	0	435
Amazonas	Men	2020	<5	75.9	70.57	80.53	226	290
Ancash	Men	2020	<5	73.85	67.47	79.36	169	232
Apurimac	Men	2020	<5	78.94	70.84	85.26	218	253
Arequipa	Men	2020	<5	73.58	66.44	79.67	175	228
Ayacucho	Men	2020	<5	74.76	66.12	81.8	225	285
Cajamarca	Men	2020	<5	73.66	67.26	79.2	193	249
Callao	Men	2020	<5	69.86	63.72	75.37	198	272
Cusco	Men	2020	<5	83.21	76.06	88.55	183	218
Huancavelica	Men	2020	<5	76.51	70.15	81.86	201	260
Huanuco	Men	2020	<5	74.2	66.97	80.3	203	262
Ica	Men	2020	<5	70.42	63.58	76.44	151	216
Junin	Men	2020	<5	78.22	67.71	86.01	166	212
La Libertad	Men	2020	<5	76.37	69.76	81.91	196	234
Lambayeque	Men	2020	<5	70.62	62.77	77.41	178	233
Lima	Men	2020	<5	72.95	69.42	76.22	675	920
Loreto	Men	2020	<5	72.95	67.42	77.84	194	259
Madre de Dios	Men	2020	<5	80.68	74.84	85.43	192	248
Moquegua	Men	2020	<5	73.24	66.51	79.04	182	248
Pasco	Men	2020	<5	82.52	76.01	87.55	178	218
Piura	Men	2020	<5	72.96	66.73	78.4	221	272
Puno	Men	2020	<5	71.19	63.17	78.06	196	262
San Martin	Men	2020	<5	76.22	69.83	81.61	226	282

Tacna	Men	2020	<5	74.36	67.09	80.49	155	221
Tumbes	Men	2020	<5	71.93	65.8	77.34	188	268
Ucayali	Men	2020	<5	75.4	69.61	80.4	180	246
Amazonas	Women	2020	<5	81.37	74.58	86.67	223	256
Ancash	Women	2020	<5	74.02	66.25	80.52	222	288
Apurimac	Women	2020	<5	77.48	69.15	84.09	231	272
Arequipa	Women	2020	<5	82.94	77.08	87.54	230	267
Ayacucho	Women	2020	<5	82.3	77.7	86.12	254	311
Cajamarca	Women	2020	<5	80.23	74.55	84.9	248	285
Callao	Women	2020	<5	79.35	74.36	83.59	219	270
Cusco	Women	2020	<5	87.27	80.77	91.79	258	288
Huancavelica	Women	2020	<5	73.65	66.86	79.47	242	313
Huanuco	Women	2020	<5	82.51	76.49	87.24	271	320
Ica	Women	2020	<5	80.08	73.49	85.36	237	280
Junin	Women	2020	<5	82.19	76.13	86.98	281	325
La Libertad	Women	2020	<5	77.75	71.06	83.26	232	288
Lambayeque	Women	2020	<5	78.32	72.92	82.9	241	299
Lima	Women	2020	<5	83.71	80.92	86.16	792	964
Loreto	Women	2020	<5	84.7	79.91	88.51	231	272
Madre de Dios	Women	2020	<5	93.08	88.92	95.75	220	236
Moquegua	Women	2020	<5	85.73	79.75	90.16	254	294
Pasco	Women	2020	<5	83.05	77.11	87.69	226	268
Piura	Women	2020	<5	82.14	76	86.98	241	284
Puno	Women	2020	<5	73.45	67.95	78.3	264	336
San Martin	Women	2020	<5	86.18	80.18	90.58	250	280
Tacna	Women	2020	<5	84.94	79.82	88.95	257	304
Tumbes	Women	2020	<5	81.2	75.93	85.53	228	286
Ucayali	Women	2020	<5	84.07	78.47	88.43	252	295
Amazonas	Men	2020	5-9	17.74	13.24	23.35	42	290

Ancash	Men	2020	5-9	12.47	8.1	18.72	32	232
Apurimac	Men	2020	5-9	11.9	7.09	19.3	20	253
Arequipa	Men	2020	5-9	14.21	9.85	20.08	27	228
Ayacucho	Men	2020	5-9	19.16	13.06	27.2	40	285
Cajamarca	Men	2020	5-9	17.57	12.8	23.63	38	249
Callao	Men	2020	5-9	17.41	12.7	23.39	44	272
Cusco	Men	2020	5-9	11.21	7.53	16.38	25	218
Huancavelica	Men	2020	5-9	11.88	8.79	15.87	31	260
Huanuco	Men	2020	5-9	15.4	11.45	20.4	41	262
Ica	Men	2020	5-9	14.64	10.55	19.96	33	216
Junin	Men	2020	5-9	13.51	7.89	22.14	30	212
La Libertad	Men	2020	5-9	15.47	10.67	21.91	26	234
Lambayeque	Men	2020	5-9	18.79	13.38	25.74	35	233
Lima	Men	2020	5-9	17.96	15.24	21.04	154	920
Loreto	Men	2020	5-9	16.5	12.05	22.18	44	259
Madre de Dios	Men	2020	5-9	13.01	9.16	18.16	41	248
Moquegua	Men	2020	5-9	17.24	12.73	22.92	46	248
Pasco	Men	2020	5-9	10.81	7.19	15.93	26	218
Piura	Men	2020	5-9	17.17	12.24	23.56	33	272
Puno	Men	2020	5-9	17.5	12.71	23.61	43	262
San Martin	Men	2020	5-9	15.07	10.84	20.58	35	282
Tacna	Men	2020	5-9	17.51	12.23	24.45	44	221
Tumbes	Men	2020	5-9	18.9	14.62	24.08	57	268
Ucayali	Men	2020	5-9	19.71	15.42	24.85	55	246
Amazonas	Women	2020	5-9	10.57	6.49	16.77	18	256
Ancash	Women	2020	5-9	15.83	11.78	20.95	46	288
Apurimac	Women	2020	5-9	15.34	10.34	22.17	29	272
Arequipa	Women	2020	5-9	9.86	6.64	14.41	26	267
Ayacucho	Women	2020	5-9	12.19	9.12	16.11	44	311

Cajamarca	Women	2020	5-9	10.34	6.7	15.61	23	285
Callao	Women	2020	5-9	12.6	9.23	16.96	33	270
Cusco	Women	2020	5-9	8.93	5.67	13.79	22	288
Huancavelica	Women	2020	5-9	17.98	13.31	23.85	51	313
Huanuco	Women	2020	5-9	11.54	7.9	16.56	33	320
Ica	Women	2020	5-9	14.06	9.76	19.85	33	280
Junin	Women	2020	5-9	10.66	7.01	15.89	28	325
La Libertad	Women	2020	5-9	17.97	13.64	23.31	46	288
Lambayeque	Women	2020	5-9	15.57	11.61	20.57	40	299
Lima	Women	2020	5-9	11.2	9.17	13.62	127	964
Loreto	Women	2020	5-9	11	7.75	15.39	31	272
Madre de Dios	Women	2020	5-9	5	2.74	8.96	12	236
Moquegua	Women	2020	5-9	11.09	7.23	16.62	32	294
Pasco	Women	2020	5-9	11.53	7.71	16.91	29	268
Piura	Women	2020	5-9	12.27	8.9	16.67	33	284
Puno	Women	2020	5-9	20.19	16.21	24.86	54	336
San Martin	Women	2020	5-9	7.71	4.6	12.64	19	280
Tacna	Women	2020	5-9	10.24	6.86	15.02	33	304
Tumbes	Women	2020	5-9	14.02	10.45	18.57	45	286
Ucayali	Women	2020	5-9	13.61	9.7	18.77	38	295
Amazonas	Men	2020	10-19	5.39	3.44	8.35	19	290
Ancash	Men	2020	10-19	12.47	8.16	18.58	29	232
Apurimac	Men	2020	10-19	9.15	5.42	15.06	15	253
Arequipa	Men	2020	10-19	11.25	7.26	17.02	24	228
Ayacucho	Men	2020	10-19	5.11	3.01	8.57	18	285
Cajamarca	Men	2020	10-19	7.68	4.4	13.08	16	249
Callao	Men	2020	10-19	11.39	7.74	16.46	27	272
Cusco	Men	2020	10-19	5.58	2.68	11.23	10	218
Huancavelica	Men	2020	10-19	11.24	7.77	15.99	27	260

Huanuco	Men	2020	10-19	10.1	5.97	16.6	17	262
Ica	Men	2020	10-19	14.05	10.04	19.31	31	216
Junin	Men	2020	10-19	7.04	2.63	17.53	13	212
La Libertad	Men	2020	10-19	7.49	3.99	13.62	11	234
Lambayeque	Men	2020	10-19	9.36	5.65	15.11	18	233
Lima	Men	2020	10-19	7.7	5.81	10.13	79	920
Loreto	Men	2020	10-19	9.7	6.13	15	19	259
Madre de Dios	Men	2020	10-19	5.7	3.04	10.45	14	248
Moquegua	Men	2020	10-19	8.14	4.7	13.72	17	248
Pasco	Men	2020	10-19	5.51	3.11	9.59	13	218
Piura	Men	2020	10-19	9.22	5.58	14.85	17	272
Puno	Men	2020	10-19	10.6	5.89	18.35	21	262
San Martin	Men	2020	10-19	7.64	4.59	12.45	19	282
Tacna	Men	2020	10-19	7.29	4.38	11.89	20	221
Tumbes	Men	2020	10-19	7.85	5.2	11.69	20	268
Ucayali	Men	2020	10-19	4.88	2.38	9.74	11	246
Amazonas	Women	2020	10-19	8.06	4.5	14.01	15	256
Ancash	Women	2020	10-19	10.02	5.03	18.98	19	288
Apurimac	Women	2020	10-19	7.18	3.25	15.12	12	272
Arequipa	Women	2020	10-19	7.2	3.66	13.69	11	267
Ayacucho	Women	2020	10-19	5.51	2.84	10.41	13	311
Cajamarca	Women	2020	10-19	9.43	5.78	15.02	14	285
Callao	Women	2020	10-19	8.05	5.01	12.69	18	270
Cusco	Women	2020	10-19	3.8	1.67	8.42	8	288
Huancavelica	Women	2020	10-19	7.75	4.58	12.82	18	313
Huanuco	Women	2020	10-19	5.95	3.26	10.62	16	320
Ica	Women	2020	10-19	5.86	2.92	11.41	10	280
Junin	Women	2020	10-19	7.15	4.45	11.28	16	325
La Libertad	Women	2020	10-19	4.28	2	8.93	10	288

Lambayeque	Women	2020	10-19	6.11	3.77	9.76	18	299
Lima	Women	2020	10-19	5.09	3.62	7.1	45	964
Loreto	Women	2020	10-19	4.3	2.3	7.91	10	272
Madre de Dios	Women	2020	10-19	1.92	0.69	5.22	4	236
Moquegua	Women	2020	10-19	3.18	1.56	6.4	8	294
Pasco	Women	2020	10-19	5.42	3.17	9.11	13	268
Piura	Women	2020	10-19	5.02	1.93	12.4	9	284
Puno	Women	2020	10-19	6.36	4.09	9.76	18	336
San Martin	Women	2020	10-19	5.52	3.05	9.79	10	280
Tacna	Women	2020	10-19	4.81	2.6	8.75	14	304
Tumbes	Women	2020	10-19	4.39	2.48	7.65	12	286
Ucayali	Women	2020	10-19	2.32	0.95	5.56	5	295
Amazonas	Men	2020	20-29	0.97	0.27	3.43	3	290
Ancash	Men	2020	20-29	1.22	0.3	4.75	2	232
Apurimac	Men	2020	20-29	0	0	0	0	253
Arequipa	Men	2020	20-29	0.96	0.23	3.84	2	228
Ayacucho	Men	2020	20-29	0.97	0.22	4.22	2	285
Cajamarca	Men	2020	20-29	1.08	0.27	4.31	2	249
Callao	Men	2020	20-29	1.34	0.28	6.1	3	272
Cusco	Men	2020	20-29	0	0	0	0	218
Huancavelica	Men	2020	20-29	0.37	0.05	2.65	1	260
Huanuco	Men	2020	20-29	0.3	0.04	2.21	1	262
Ica	Men	2020	20-29	0.9	0.12	6.37	1	216
Junin	Men	2020	20-29	1.24	0.32	4.73	3	212
La Libertad	Men	2020	20-29	0.67	0.09	4.71	1	234
Lambayeque	Men	2020	20-29	1.23	0.3	4.98	2	233
Lima	Men	2020	20-29	1.39	0.74	2.59	12	920
Loreto	Men	2020	20-29	0.85	0.19	3.79	2	259
Madre de Dios	Men	2020	20-29	0.61	0.08	4.33	1	248

Moquegua	Men	2020	20-29	1.38	0.45	4.19	3	248
Pasco	Men	2020	20-29	1.17	0.16	8.13	1	218
Piura	Men	2020	20-29	0.65	0.09	4.58	1	272
Puno	Men	2020	20-29	0.71	0.18	2.82	2	262
San Martin	Men	2020	20-29	0.63	0.08	4.53	1	282
Tacna	Men	2020	20-29	0.84	0.13	5.06	2	221
Tumbes	Men	2020	20-29	1.31	0.41	4.12	3	268
Ucayali	Men	2020	20-29	0	0	0	0	246
Amazonas	Women	2020	20-29	0	0	0	0	256
Ancash	Women	2020	20-29	0.13	0.02	0.96	1	288
Apurimac	Women	2020	20-29	0	0	0	0	272
Arequipa	Women	2020	20-29	0	0	0	0	267
Ayacucho	Women	2020	20-29	0	0	0	0	311
Cajamarca	Women	2020	20-29	0	0	0	0	285
Callao	Women	2020	20-29	0	0	0	0	270
Cusco	Women	2020	20-29	0	0	0	0	288
Huancavelica	Women	2020	20-29	0.63	0.13	2.88	2	313
Huanuco	Women	2020	20-29	0	0	0	0	320
Ica	Women	2020	20-29	0	0	0	0	280
Junin	Women	2020	20-29	0	0	0	0	325
La Libertad	Women	2020	20-29	0	0	0	0	288
Lambayeque	Women	2020	20-29	0	0	0	0	299
Lima	Women	2020	20-29	0	0	0	0	964
Loreto	Women	2020	20-29	0	0	0	0	272
Madre de Dios	Women	2020	20-29	0	0	0	0	236
Moquegua	Women	2020	20-29	0	0	0	0	294
Pasco	Women	2020	20-29	0	0	0	0	268
Piura	Women	2020	20-29	0.57	0.08	4.07	1	284
Puno	Women	2020	20-29	0	0	0	0	336

San Martin	Women	2020	20-29	0.59	0.08	4.16	1	280
Tacna	Women	2020	20-29	0	0	0	0	304
Tumbes	Women	2020	20-29	0.39	0.05	2.88	1	286
Ucayali	Women	2020	20-29	0	0	0	0	295
Amazonas	Men	2020	30+	0	0	0	0	290
Ancash	Men	2020	30+	0	0	0	0	232
Apurimac	Men	2020	30+	0	0	0	0	253
Arequipa	Men	2020	30+	0	0	0	0	228
Ayacucho	Men	2020	30+	0	0	0	0	285
Cajamarca	Men	2020	30+	0	0	0	0	249
Callao	Men	2020	30+	0	0	0	0	272
Cusco	Men	2020	30+	0	0	0	0	218
Huancavelica	Men	2020	30+	0	0	0	0	260
Huanuco	Men	2020	30+	0	0	0	0	262
Ica	Men	2020	30+	0	0	0	0	216
Junin	Men	2020	30+	0	0	0	0	212
La Libertad	Men	2020	30+	0	0	0	0	234
Lambayeque	Men	2020	30+	0	0	0	0	233
Lima	Men	2020	30+	0	0	0	0	920
Loreto	Men	2020	30+	0	0	0	0	259
Madre de Dios	Men	2020	30+	0	0	0	0	248
Moquegua	Men	2020	30+	0	0	0	0	248
Pasco	Men	2020	30+	0	0	0	0	218
Piura	Men	2020	30+	0	0	0	0	272
Puno	Men	2020	30+	0	0	0	0	262
San Martin	Men	2020	30+	0.43	0.06	3.1	1	282
Tacna	Men	2020	30+	0	0	0	0	221
Tumbes	Men	2020	30+	0	0	0	0	268
Ucayali	Men	2020	30+	0	0	0	0	246

Amazonas	Women	2020	30+	0	0	0	0	256
Ancash	Women	2020	30+	0	0	0	0	288
Apurimac	Women	2020	30+	0	0	0	0	272
Arequipa	Women	2020	30+	0	0	0	0	267
Ayacucho	Women	2020	30+	0	0	0	0	311
Cajamarca	Women	2020	30+	0	0	0	0	285
Callao	Women	2020	30+	0	0	0	0	270
Cusco	Women	2020	30+	0	0	0	0	288
Huancavelica	Women	2020	30+	0	0	0	0	313
Huanuco	Women	2020	30+	0	0	0	0	320
Ica	Women	2020	30+	0	0	0	0	280
Junin	Women	2020	30+	0	0	0	0	325
La Libertad	Women	2020	30+	0	0	0	0	288
Lambayeque	Women	2020	30+	0	0	0	0	299
Lima	Women	2020	30+	0	0	0	0	964
Loreto	Women	2020	30+	0	0	0	0	272
Madre de Dios	Women	2020	30+	0	0	0	0	236
Moquegua	Women	2020	30+	0	0	0	0	294
Pasco	Women	2020	30+	0	0	0	0	268
Piura	Women	2020	30+	0	0	0	0	284
Puno	Women	2020	30+	0	0	0	0	336
San Martin	Women	2020	30+	0	0	0	0	280
Tacna	Women	2020	30+	0	0	0	0	304
Tumbes	Women	2020	30+	0	0	0	0	286
Ucayali	Women	2020	30+	0	0	0	0	295

The numerator and denominator will not exactly match the proportion (%) because the proportion was computed accounting for the complex survey design, whereas the numerator denominator are observed data.

Supplementary Table 6. Proportion of eligible and non-eligible subjects for antihypertensive treatment according to the selected guidelines by year sex, and age group (national level)

Year	Sex	Age	Eligible antihypertensive treatment	Guidelines	Proportion with antihypertensive treatment	Proportion with antihypertensive treatment lower	Proportion with antihypertensive treatment upper	Numerator	Denominator
2015	Men	<60	No	MINSA	1.25	0.93	1.66	95	7029
2015	Men	<60	Yes	MINSA	13.47	10.36	17.35	119	966
2015	Women	<60	No	MINSA	4.29	3.6	5.11	277	8171
2015	Women	<60	Yes	MINSA	33.91	28.88	39.33	174	554
2015	Men	60+	No	MINSA	8.76	6.65	11.46	93	1104
2015	Men	60+	Yes	MINSA	33.15	27.66	39.14	178	559
2015	Women	60+	No	MINSA	15.41	13.08	18.07	214	1408
2015	Women	60+	Yes	MINSA	46.39	41.15	51.71	305	696
2016	Men	<60	No	MINSA	1.37	1.01	1.86	78	6587
2016	Men	<60	Yes	MINSA	13.68	10.36	17.85	130	945
2016	Women	<60	No	MINSA	3.48	2.97	4.09	267	8309
2016	Women	<60	Yes	MINSA	28.72	23.55	34.52	165	539
2016	Men	60+	No	MINSA	8.98	6.94	11.54	99	1187
2016	Men	60+	Yes	MINSA	34.02	29.27	39.11	207	647
2016	Women	60+	No	MINSA	15.75	13.21	18.68	195	1408
2016	Women	60+	Yes	MINSA	42.64	37.58	47.86	289	659
2017	Men	<60	No	MINSA	1.44	1.03	2.02	84	6648
2017	Men	<60	Yes	MINSA	8.07	6	10.78	95	958
2017	Women	<60	No	MINSA	3.27	2.68	4	245	8401
2017	Women	<60	Yes	MINSA	27.39	21.91	33.65	162	590
2017	Men	60+	No	MINSA	9.22	6.67	12.61	102	1269
2017	Men	60+	Yes	MINSA	27.45	22.73	32.73	188	701

2017	Women	60+	No	MINSA	14.78	12.01	18.05	205	1513
2017	Women	60+	Yes	MINSA	44.85	38.94	50.9	299	707
2018	Men	<60	No	MINSA	1.52	1.16	2	115	6900
2018	Men	<60	Yes	MINSA	9.48	7.51	11.91	132	1255
2018	Women	<60	No	MINSA	3.33	2.78	3.99	280	9059
2018	Women	<60	Yes	MINSA	27.53	22.89	32.71	187	715
2018	Men	60+	No	MINSA	7.68	5.62	10.4	84	1286
2018	Men	60+	Yes	MINSA	35.44	30.35	40.88	228	704
2018	Women	60+	No	MINSA	16.06	13.5	19	224	1603
2018	Women	60+	Yes	MINSA	49.51	43.86	55.16	326	715
2019	Men	<60	No	MINSA	1.87	1.31	2.68	103	6597
2019	Men	<60	Yes	MINSA	13.48	10.58	17.02	125	1096
2019	Women	<60	No	MINSA	3.55	2.92	4.31	263	8709
2019	Women	<60	Yes	MINSA	32.84	27.12	39.12	188	632
2019	Men	60+	No	MINSA	13.02	9.9	16.94	126	1331
2019	Men	60+	Yes	MINSA	37.59	32.16	43.36	236	707
2019	Women	60+	No	MINSA	17.18	14.45	20.32	242	1667
2019	Women	60+	Yes	MINSA	46.91	41.33	52.56	345	759
2020	Men	<60	No	MINSA	1.92	1.31	2.79	61	4579
2020	Men	<60	Yes	MINSA	10.64	7.76	14.41	84	846
2020	Women	<60	No	MINSA	4.1	3.32	5.06	214	5670
2020	Women	<60	Yes	MINSA	23.68	18.55	29.72	119	518
2020	Men	60+	No	MINSA	7.12	4.88	10.27	59	941
2020	Men	60+	Yes	MINSA	34.75	28.53	41.53	159	522
2020	Women	60+	No	MINSA	20.93	17.45	24.89	181	1136
2020	Women	60+	Yes	MINSA	41.43	35.78	47.32	204	557
2015	Men	<60	No	WHO	2.12	1.67	2.7	159	7813
2015	Men	<60	Yes	WHO	33.04	23.56	44.13	55	182
2015	Women	<60	No	WHO	5.64	4.88	6.52	383	8586

2015	Women	<60	Yes	WHO	53.43	42.52	64.02	68	139
2015	Men	60+	No	WHO	10.14	8.03	12.73	119	1260
2015	Men	60+	Yes	WHO	37.59	30.95	44.73	152	403
2015	Women	60+	No	WHO	18.53	16.16	21.16	304	1689
2015	Women	60+	Yes	WHO	55.4	48.48	62.12	215	415
2016	Men	<60	No	WHO	2.14	1.71	2.67	151	7350
2016	Men	<60	Yes	WHO	32.43	21.71	45.38	57	182
2016	Women	<60	No	WHO	4.47	3.89	5.13	365	8708
2016	Women	<60	Yes	WHO	51.13	39.28	62.86	67	140
2016	Men	60+	No	WHO	9.73	7.75	12.16	129	1362
2016	Men	60+	Yes	WHO	40.6	34.74	46.73	177	472
2016	Women	60+	No	WHO	16.83	14.42	19.54	259	1644
2016	Women	60+	Yes	WHO	52.1	45.38	58.74	225	423
2017	Men	<60	No	WHO	1.93	1.49	2.5	137	7420
2017	Men	<60	Yes	WHO	16.56	10.44	25.25	42	186
2017	Women	<60	No	WHO	4.43	3.75	5.24	333	8827
2017	Women	<60	Yes	WHO	45.49	34.06	57.42	74	164
2017	Men	60+	No	WHO	9.02	6.67	12.1	116	1431
2017	Men	60+	Yes	WHO	33.47	27.69	39.78	174	539
2017	Women	60+	No	WHO	17.46	14.78	20.52	281	1764
2017	Women	60+	Yes	WHO	52.48	44.74	60.11	223	456
2018	Men	<60	No	WHO	2.11	1.72	2.59	174	7868
2018	Men	<60	Yes	WHO	21.69	15.75	29.1	73	287
2018	Women	<60	No	WHO	4.41	3.77	5.16	374	9582
2018	Women	<60	Yes	WHO	45.89	35.66	56.48	93	192
2018	Men	60+	No	WHO	9.43	7.25	12.17	109	1458
2018	Men	60+	Yes	WHO	39.76	33.82	46.03	203	532
2018	Women	60+	No	WHO	18.68	16.13	21.54	313	1885
2018	Women	60+	Yes	WHO	58.73	51.18	65.89	237	433

2019	Men	<60	No	WHO	2.68	2.07	3.47	161	7448
2019	Men	<60	Yes	WHO	31.89	23.25	41.99	67	245
2019	Women	<60	No	WHO	4.65	3.93	5.49	360	9160
2019	Women	<60	Yes	WHO	52.39	41.52	63.04	91	181
2019	Men	60+	No	WHO	14.34	11.32	18	155	1508
2019	Men	60+	Yes	WHO	42.67	35.98	49.62	207	530
2019	Women	60+	No	WHO	19.49	16.85	22.43	338	1967
2019	Women	60+	Yes	WHO	56.09	48.88	63.06	249	459
2020	Men	<60	No	WHO	3.07	2.31	4.06	110	5236
2020	Men	<60	Yes	WHO	17.68	10.72	27.75	35	189
2020	Women	<60	No	WHO	5.15	4.29	6.16	279	6042
2020	Women	<60	Yes	WHO	39.56	28.09	52.31	54	146
2020	Men	60+	No	WHO	8.43	6.2	11.37	82	1091
2020	Men	60+	Yes	WHO	42.79	35.32	50.59	136	372
2020	Women	60+	No	WHO	22.17	19.12	25.55	238	1369
2020	Women	60+	Yes	WHO	49.19	41.79	56.63	147	324

The numerator and denominator will not exactly match the proportion (%) because the proportion was computed accounting for the complex survey design, whereas the numerator denominator are observed data.

Supplementary Table 7. Proportion of eligible and non-eligible subjects for antihypertensive treatment according to the selected guidelines by year and region (sub-national level)

Region	Year	Guidelines	Eligible antihypertensive treatment	Proportion with treatment	Proportion with treatment lower	Proportion with treatment upper	Numerator	Denominator
Amazonas	2015	MINSA	No	3.03	1.79	5.09	20	669
Amazonas	2015	MINSA	Yes	28.19	20.52	37.37	34	122
Ancash	2015	MINSA	No	3.07	1.89	4.96	19	660
Ancash	2015	MINSA	Yes	23.96	15.72	34.72	31	108
Apurimac	2015	MINSA	No	3.03	1.82	4.99	18	570
Apurimac	2015	MINSA	Yes	22.15	14.18	32.9	21	96
Arequipa	2015	MINSA	No	5.25	3.54	7.73	28	677
Arequipa	2015	MINSA	Yes	31.05	21.08	43.16	25	88
Ayacucho	2015	MINSA	No	3.56	2.31	5.45	24	783
Ayacucho	2015	MINSA	Yes	22.51	13.63	34.86	23	100
Cajamarca	2015	MINSA	No	4.45	2.9	6.78	20	614
Cajamarca	2015	MINSA	Yes	20.82	13.61	30.49	17	92
Callao	2015	MINSA	No	5.97	3.96	8.91	28	641
Callao	2015	MINSA	Yes	31.61	23.85	40.55	43	143
Cusco	2015	MINSA	No	4.38	2.95	6.45	23	630
Cusco	2015	MINSA	Yes	14.32	7	27.05	10	80
Huancavelica	2015	MINSA	No	4.34	2.82	6.63	25	624
Huancavelica	2015	MINSA	Yes	17.46	10.18	28.31	17	78
Huanuco	2015	MINSA	No	2.56	1.49	4.36	19	751
Huanuco	2015	MINSA	Yes	19.67	11.7	31.16	16	94
Ica	2015	MINSA	No	6.91	4.72	10.01	37	664
Ica	2015	MINSA	Yes	35.86	27.42	45.28	50	139

Junin	2015	MINSA	No	3.37	2.08	5.41	22	651
Junin	2015	MINSA	Yes	16.81	9.67	27.61	14	73
La Libertad	2015	MINSA	No	3.89	2.1	7.09	17	648
La Libertad	2015	MINSA	Yes	34.03	23.81	45.99	35	101
Lambayeque	2015	MINSA	No	5.6	3.78	8.23	38	680
Lambayeque	2015	MINSA	Yes	35.76	26.31	46.47	40	111
Lima	2015	MINSA	No	4.85	3.68	6.36	74	2007
Lima	2015	MINSA	Yes	34.95	28.83	41.62	126	391
Loreto	2015	MINSA	No	7.91	5.87	10.58	50	656
Loreto	2015	MINSA	Yes	35.3	25.17	46.95	32	91
Madre de Dios	2015	MINSA	No	5.86	3.66	9.26	31	611
Madre de Dios	2015	MINSA	Yes	23.79	14.11	37.25	14	56
Moquegua	2015	MINSA	No	3.87	2.46	6.05	21	649
Moquegua	2015	MINSA	Yes	28.31	18.99	39.95	28	96
Pasco	2015	MINSA	No	1.31	0.62	2.75	8	620
Pasco	2015	MINSA	Yes	16.75	9.61	27.57	13	79
Piura	2015	MINSA	No	3.66	2.34	5.69	20	621
Piura	2015	MINSA	Yes	41.72	33.1	50.88	56	154
Puno	2015	MINSA	No	2.02	1.04	3.88	13	632
Puno	2015	MINSA	Yes	10.11	4.25	22.19	8	85
San Martin	2015	MINSA	No	5.7	3.95	8.16	37	677
San Martin	2015	MINSA	Yes	37.31	26.93	49.01	41	109
Tacna	2015	MINSA	No	4.18	2.66	6.5	21	608
Tacna	2015	MINSA	Yes	18.33	12.37	26.29	23	123
Tumbes	2015	MINSA	No	6.53	4.45	9.49	34	684
Tumbes	2015	MINSA	Yes	38.5	29.09	48.86	39	104
Ucayali	2015	MINSA	No	5.65	3.68	8.56	32	685
Ucayali	2015	MINSA	Yes	31.48	18.93	47.48	20	62
Amazonas	2016	MINSA	No	2.57	1.3	5.01	14	672

Amazonas	2016	MINSA	Yes	32.89	22.94	44.66	44	130
Ancash	2016	MINSA	No	3.48	2.14	5.6	20	612
Ancash	2016	MINSA	Yes	22.82	14.21	34.56	25	105
Apurimac	2016	MINSA	No	4.8	3.18	7.17	27	601
Apurimac	2016	MINSA	Yes	19.25	12.07	29.29	17	85
Arequipa	2016	MINSA	No	5.81	3.9	8.56	30	642
Arequipa	2016	MINSA	Yes	23.21	13.85	36.24	17	84
Ayacucho	2016	MINSA	No	3.67	2.32	5.76	21	798
Ayacucho	2016	MINSA	Yes	23.47	15.47	33.93	25	111
Cajamarca	2016	MINSA	No	4.57	3.07	6.75	22	564
Cajamarca	2016	MINSA	Yes	30.75	22.23	40.81	32	106
Callao	2016	MINSA	No	5.59	3.69	8.37	28	615
Callao	2016	MINSA	Yes	35.44	28.41	43.17	45	134
Cusco	2016	MINSA	No	3	1.8	4.97	17	649
Cusco	2016	MINSA	Yes	21.83	11.41	37.69	18	79
Huancavelica	2016	MINSA	No	3.3	2.07	5.21	20	615
Huancavelica	2016	MINSA	Yes	23.93	14.31	37.21	17	78
Huanuco	2016	MINSA	No	3.57	2.14	5.9	22	776
Huanuco	2016	MINSA	Yes	22.92	14.55	34.16	20	86
Ica	2016	MINSA	No	5.68	3.37	9.4	24	622
Ica	2016	MINSA	Yes	31.53	23.64	40.65	46	141
Junin	2016	MINSA	No	2.84	1.39	5.74	12	668
Junin	2016	MINSA	Yes	21.92	13.29	33.97	18	84
La Libertad	2016	MINSA	No	4.39	2.72	7.02	20	608
La Libertad	2016	MINSA	Yes	30.01	21.03	40.84	25	86
Lambayeque	2016	MINSA	No	5.25	3.74	7.34	37	707
Lambayeque	2016	MINSA	Yes	34.74	26.43	44.1	48	127
Lima	2016	MINSA	No	4.19	3.17	5.51	66	1868
Lima	2016	MINSA	Yes	28.66	23.64	34.27	112	402

Loreto	2016	MINSA	No	4.29	2.92	6.26	26	619
Loreto	2016	MINSA	Yes	30.2	22.13	39.72	42	128
Madre de Dios	2016	MINSA	No	6.22	3.79	10.03	30	615
Madre de Dios	2016	MINSA	Yes	32.7	18.97	50.19	16	57
Moquegua	2016	MINSA	No	6.46	4.24	9.74	27	660
Moquegua	2016	MINSA	Yes	32.56	21.44	46.07	33	88
Pasco	2016	MINSA	No	3.53	2.04	6.02	20	621
Pasco	2016	MINSA	Yes	10.54	5.48	19.31	11	83
Piura	2016	MINSA	No	6.73	4.75	9.44	35	640
Piura	2016	MINSA	Yes	30.16	21.67	40.26	43	140
Puno	2016	MINSA	No	1.61	0.8	3.2	9	647
Puno	2016	MINSA	Yes	20.09	10.85	34.2	11	72
San Martin	2016	MINSA	No	4.08	2.61	6.33	27	668
San Martin	2016	MINSA	Yes	40.72	30.81	51.44	39	100
Tacna	2016	MINSA	No	3.66	2.28	5.83	22	665
Tacna	2016	MINSA	Yes	23.4	15.82	33.18	29	125
Tumbes	2016	MINSA	No	6.42	4.11	9.9	34	671
Tumbes	2016	MINSA	Yes	35.14	24.41	47.62	36	98
Ucayali	2016	MINSA	No	4.48	3.02	6.6	29	668
Ucayali	2016	MINSA	Yes	29.47	18.46	43.55	22	61
Amazonas	2017	MINSA	No	4.35	2.93	6.43	30	672
Amazonas	2017	MINSA	Yes	29.53	21.38	39.23	41	138
Ancash	2017	MINSA	No	3.92	2.41	6.31	21	672
Ancash	2017	MINSA	Yes	27.1	17.97	38.68	26	111
Apurimac	2017	MINSA	No	2.39	1.31	4.31	14	653
Apurimac	2017	MINSA	Yes	15.42	8.62	26.04	14	102
Arequipa	2017	MINSA	No	5.68	3.98	8.05	32	682
Arequipa	2017	MINSA	Yes	26.84	17.94	38.12	22	89
Ayacucho	2017	MINSA	No	3.02	2	4.55	27	762

Ayacucho	2017	MINSA	Yes	24.34	17.29	33.1	31	131
Cajamarca	2017	MINSA	No	2.86	1.75	4.63	17	616
Cajamarca	2017	MINSA	Yes	32.01	23.68	41.66	40	136
Callao	2017	MINSA	No	5.7	4.05	7.96	32	607
Callao	2017	MINSA	Yes	30.01	22.76	38.43	41	139
Cusco	2017	MINSA	No	3.67	2.08	6.4	18	626
Cusco	2017	MINSA	Yes	27.79	17.27	41.52	19	75
Huancavelica	2017	MINSA	No	2.31	1.34	3.94	16	703
Huancavelica	2017	MINSA	Yes	26.15	15.87	39.93	24	92
Huanuco	2017	MINSA	No	2.53	1.57	4.05	19	760
Huanuco	2017	MINSA	Yes	12.91	6.32	24.57	9	80
Ica	2017	MINSA	No	4.91	3.37	7.11	31	687
Ica	2017	MINSA	Yes	26.27	19.45	34.45	44	165
Junin	2017	MINSA	No	2.5	1.49	4.18	18	662
Junin	2017	MINSA	Yes	19.79	11.32	32.28	16	84
La Libertad	2017	MINSA	No	4.48	3.05	6.53	27	655
La Libertad	2017	MINSA	Yes	24.17	15.47	35.69	22	89
Lambayeque	2017	MINSA	No	5.91	4	8.66	34	707
Lambayeque	2017	MINSA	Yes	38.41	28.26	49.69	37	97
Lima	2017	MINSA	No	4.15	3.11	5.51	61	1873
Lima	2017	MINSA	Yes	21.81	17.27	27.15	88	406
Loreto	2017	MINSA	No	4.93	3.4	7.11	30	627
Loreto	2017	MINSA	Yes	34.33	25.9	43.88	37	119
Madre de Dios	2017	MINSA	No	4.77	3.08	7.32	30	637
Madre de Dios	2017	MINSA	Yes	29.77	20.08	41.7	21	71
Moquegua	2017	MINSA	No	4.33	2.63	7.02	20	689
Moquegua	2017	MINSA	Yes	23.36	15.22	34.11	26	109
Pasco	2017	MINSA	No	1.97	0.98	3.95	12	624
Pasco	2017	MINSA	Yes	22.39	13.25	35.28	14	69

Piura	2017	MINSA	No	3.63	2.29	5.71	22	653
Piura	2017	MINSA	Yes	26.04	16.97	37.76	29	128
Puno	2017	MINSA	No	2.89	1.7	4.86	21	661
Puno	2017	MINSA	Yes	14.67	7.22	27.51	13	98
San Martin	2017	MINSA	No	3.55	2.3	5.46	22	646
San Martin	2017	MINSA	Yes	31.12	22.87	40.76	37	114
Tacna	2017	MINSA	No	3.51	2.13	5.75	18	629
Tacna	2017	MINSA	Yes	29.41	22.13	37.92	38	145
Tumbes	2017	MINSA	No	6.63	4.65	9.38	38	684
Tumbes	2017	MINSA	Yes	26.96	18.54	37.46	27	99
Ucayali	2017	MINSA	No	3.89	2.57	5.86	26	644
Ucayali	2017	MINSA	Yes	39.18	26.39	53.65	28	70
Amazonas	2018	MINSA	No	4.28	2.65	6.87	26	750
Amazonas	2018	MINSA	Yes	29.69	22.87	37.55	35	148
Ancash	2018	MINSA	No	4.28	2.69	6.74	24	707
Ancash	2018	MINSA	Yes	35.75	26.89	45.7	37	118
Apurimac	2018	MINSA	No	3.77	2.34	6.01	23	709
Apurimac	2018	MINSA	Yes	22.95	15.35	32.86	24	108
Arequipa	2018	MINSA	No	3.97	2.67	5.87	23	657
Arequipa	2018	MINSA	Yes	23.1	17.03	30.54	33	134
Ayacucho	2018	MINSA	No	2.84	1.84	4.37	21	809
Ayacucho	2018	MINSA	Yes	18.89	12.94	26.74	23	120
Cajamarca	2018	MINSA	No	2.74	1.67	4.47	19	732
Cajamarca	2018	MINSA	Yes	34.79	26.07	44.67	44	130
Callao	2018	MINSA	No	6.22	4.29	8.93	33	623
Callao	2018	MINSA	Yes	30.67	22.88	39.74	44	146
Cusco	2018	MINSA	No	3.77	2.41	5.84	24	682
Cusco	2018	MINSA	Yes	19.03	10.34	32.41	16	87
Huancavelica	2018	MINSA	No	3.68	2.46	5.49	26	765

Huancavelica	2018	MINSA	Yes	16.67	9.41	27.8	16	91
Huanuco	2018	MINSA	No	3.2	2.09	4.88	26	776
Huanuco	2018	MINSA	Yes	20.74	13.8	29.96	20	101
Ica	2018	MINSA	No	6.65	4.83	9.09	40	680
Ica	2018	MINSA	Yes	23.23	16.4	31.82	36	151
Junin	2018	MINSA	No	2.47	1.38	4.36	13	653
Junin	2018	MINSA	Yes	13.52	7.62	22.87	13	97
La Libertad	2018	MINSA	No	4.11	2.77	6.07	23	670
La Libertad	2018	MINSA	Yes	38.75	30.13	48.14	41	111
Lambayeque	2018	MINSA	No	4.63	3.15	6.74	31	734
Lambayeque	2018	MINSA	Yes	37	28.91	45.9	48	134
Lima	2018	MINSA	No	3.87	3	4.97	78	2228
Lima	2018	MINSA	Yes	27.19	23	31.84	129	509
Loreto	2018	MINSA	No	8.1	5.75	11.31	49	618
Loreto	2018	MINSA	Yes	35.5	26.49	45.68	46	136
Madre de Dios	2018	MINSA	No	3.77	2.32	6.07	23	609
Madre de Dios	2018	MINSA	Yes	24.17	15	36.54	19	89
Moquegua	2018	MINSA	No	4.57	3	6.91	27	719
Moquegua	2018	MINSA	Yes	31.88	22.45	43.06	31	107
Pasco	2018	MINSA	No	3.41	2	5.76	18	619
Pasco	2018	MINSA	Yes	13.79	7.98	22.78	14	106
Piura	2018	MINSA	No	5.49	3.6	8.28	25	673
Piura	2018	MINSA	Yes	24.16	17.52	32.32	38	159
Puno	2018	MINSA	No	3.87	2.49	5.96	26	723
Puno	2018	MINSA	Yes	25.07	15.62	37.69	21	105
San Martin	2018	MINSA	No	3.5	2.25	5.42	22	656
San Martin	2018	MINSA	Yes	29.91	21.25	40.28	34	127
Tacna	2018	MINSA	No	3.14	1.4	6.87	16	708
Tacna	2018	MINSA	Yes	25.73	18.32	34.87	32	144

Tumbes	2018	MINSA	No	6.47	4.53	9.15	37	661
Tumbes	2018	MINSA	Yes	33.9	26.07	42.72	44	138
Ucayali	2018	MINSA	No	5.16	3.59	7.35	30	687
Ucayali	2018	MINSA	Yes	37.54	27.49	48.78	35	93
Amazonas	2019	MINSA	No	3.87	2.58	5.77	26	696
Amazonas	2019	MINSA	Yes	31.52	23.28	41.12	45	141
Ancash	2019	MINSA	No	4.34	2.9	6.46	28	691
Ancash	2019	MINSA	Yes	24.49	18.01	32.39	32	124
Apurimac	2019	MINSA	No	2.55	1.4	4.61	19	694
Apurimac	2019	MINSA	Yes	24.42	15.24	36.74	23	103
Arequipa	2019	MINSA	No	3.81	2.36	6.1	21	629
Arequipa	2019	MINSA	Yes	31.96	23.56	41.71	38	130
Ayacucho	2019	MINSA	No	3.69	2.46	5.5	30	806
Ayacucho	2019	MINSA	Yes	20.34	13.73	29.06	26	142
Cajamarca	2019	MINSA	No	3.48	2.1	5.73	20	730
Cajamarca	2019	MINSA	Yes	27.03	18.47	37.71	28	105
Callao	2019	MINSA	No	7.15	5.23	9.7	38	585
Callao	2019	MINSA	Yes	34.76	27.56	42.73	51	153
Cusco	2019	MINSA	No	4.99	3.53	7.02	36	699
Cusco	2019	MINSA	Yes	19.52	9.98	34.66	16	81
Huancavelica	2019	MINSA	No	4.03	2.62	6.13	33	751
Huancavelica	2019	MINSA	Yes	18.73	11.97	28.09	24	99
Huanuco	2019	MINSA	No	4.06	2.53	6.44	23	755
Huanuco	2019	MINSA	Yes	28.15	17.74	41.59	24	115
Ica	2019	MINSA	No	4.19	2.83	6.16	25	635
Ica	2019	MINSA	Yes	31.84	23.45	41.61	45	137
Junin	2019	MINSA	No	2.94	1.82	4.73	17	688
Junin	2019	MINSA	Yes	20.45	13.12	30.42	17	84
La Libertad	2019	MINSA	No	4.11	2.81	5.98	24	630

La Libertad	2019	MINSA	Yes	36.82	27.67	47.03	41	103
Lambayeque	2019	MINSA	No	4.78	3.26	6.95	27	672
Lambayeque	2019	MINSA	Yes	41.83	32.52	51.76	38	94
Lima	2019	MINSA	No	5.74	4.49	7.31	81	2067
Lima	2019	MINSA	Yes	32.21	27.38	37.46	138	466
Loreto	2019	MINSA	No	8.81	6.71	11.49	48	604
Loreto	2019	MINSA	Yes	31.01	22.3	41.32	39	125
Madre de Dios	2019	MINSA	No	5.46	3.68	8.04	33	610
Madre de Dios	2019	MINSA	Yes	29.05	19.13	41.46	24	87
Moquegua	2019	MINSA	No	5.44	3.66	8.01	25	723
Moquegua	2019	MINSA	Yes	23.15	15.17	33.65	23	101
Pasco	2019	MINSA	No	3.28	1.88	5.68	14	661
Pasco	2019	MINSA	Yes	14.9	7.8	26.6	12	84
Piura	2019	MINSA	No	4.5	3.05	6.58	27	618
Piura	2019	MINSA	Yes	24.15	17.65	32.12	35	147
Puno	2019	MINSA	No	1.75	0.97	3.12	15	725
Puno	2019	MINSA	Yes	17.38	9.73	29.11	17	104
San Martin	2019	MINSA	No	6.27	4.32	9	33	660
San Martin	2019	MINSA	Yes	39.24	31	48.13	53	126
Tacna	2019	MINSA	No	3.53	2.28	5.44	21	660
Tacna	2019	MINSA	Yes	20.59	14.23	28.83	32	160
Tumbes	2019	MINSA	No	7.12	5.2	9.68	38	620
Tumbes	2019	MINSA	Yes	40.14	30.44	50.67	46	124
Ucayali	2019	MINSA	No	4.94	3.47	6.99	32	695
Ucayali	2019	MINSA	Yes	45.34	33.26	57.99	27	59
Amazonas	2020	MINSA	No	2.81	1.65	4.74	13	469
Amazonas	2020	MINSA	Yes	31.9	20.8	45.52	22	77
Ancash	2020	MINSA	No	1.82	0.84	3.93	9	424
Ancash	2020	MINSA	Yes	33.5	20.9	48.99	22	96

Apurimac	2020	MINSA	No	3.8	2.05	6.92	18	468
Apurimac	2020	MINSA	Yes	25.9	12.37	46.4	10	57
Arequipa	2020	MINSA	No	5.34	3.4	8.3	21	412
Arequipa	2020	MINSA	Yes	25.88	16.69	37.84	20	83
Ayacucho	2020	MINSA	No	4.12	2.64	6.37	18	526
Ayacucho	2020	MINSA	Yes	22.52	13.24	35.64	13	70
Cajamarca	2020	MINSA	No	2.74	1.58	4.71	12	438
Cajamarca	2020	MINSA	Yes	17.04	11.09	25.27	13	96
Callao	2020	MINSA	No	5.53	3.57	8.48	20	387
Callao	2020	MINSA	Yes	27.69	20.66	36.02	40	155
Cusco	2020	MINSA	No	3.69	2.1	6.39	16	455
Cusco	2020	MINSA	Yes	17.5	8.34	33.1	10	51
Huancavelica	2020	MINSA	No	3.72	2.33	5.89	19	492
Huancavelica	2020	MINSA	Yes	26.1	16.76	38.26	20	81
Huanuco	2020	MINSA	No	3.97	2.18	7.14	13	503
Huanuco	2020	MINSA	Yes	17.54	9.45	30.23	12	79
Ica	2020	MINSA	No	5.53	3.39	8.87	19	410
Ica	2020	MINSA	Yes	24.5	17.39	33.35	20	86
Junin	2020	MINSA	No	3.75	1.76	7.8	13	476
Junin	2020	MINSA	Yes	25.86	14.61	41.54	15	61
La Libertad	2020	MINSA	No	4.95	3	8.07	17	452
La Libertad	2020	MINSA	Yes	29.77	19.6	42.44	21	70
Lambayeque	2020	MINSA	No	7.12	4.9	10.23	26	449
Lambayeque	2020	MINSA	Yes	35.3	24.87	47.34	27	83
Lima	2020	MINSA	No	5.69	4.44	7.28	70	1436
Lima	2020	MINSA	Yes	22.23	18.3	26.74	88	448
Loreto	2020	MINSA	No	6.8	4.77	9.6	32	424
Loreto	2020	MINSA	Yes	28.13	19.84	38.22	30	107
Madre de Dios	2020	MINSA	No	5.68	3.48	9.15	23	437

Madre de Dios	2020	MINSA	Yes	19.56	10.21	34.2	11	47
Moquegua	2020	MINSA	No	2.36	1.01	5.4	9	432
Moquegua	2020	MINSA	Yes	20.19	12.89	30.19	22	110
Pasco	2020	MINSA	No	1.31	0.47	3.61	5	425
Pasco	2020	MINSA	Yes	16.15	7.49	31.4	9	61
Piura	2020	MINSA	No	6.3	4.11	9.55	24	459
Piura	2020	MINSA	Yes	33.41	23.27	45.36	28	97
Puno	2020	MINSA	No	6.15	4.24	8.85	29	529
Puno	2020	MINSA	Yes	26.96	16.88	40.17	14	69
San Martin	2020	MINSA	No	6.02	4.08	8.79	26	474
San Martin	2020	MINSA	Yes	36.01	26.64	46.59	27	88
Tacna	2020	MINSA	No	3.99	2.41	6.55	18	425
Tacna	2020	MINSA	Yes	24.26	16.46	34.24	22	100
Tumbes	2020	MINSA	No	6.9	4.86	9.73	28	441
Tumbes	2020	MINSA	Yes	29.83	21.44	39.84	36	113
Ucayali	2020	MINSA	No	3.94	2.33	6.59	17	483
Ucayali	2020	MINSA	Yes	23.41	12.55	39.44	14	58
Amazonas	2015	WHO	No	3.54	2.3	5.41	29	740
Amazonas	2015	WHO	Yes	48.87	35.81	62.09	25	51
Ancash	2015	WHO	No	4.01	2.67	5.99	29	720
Ancash	2015	WHO	Yes	40.87	26.29	57.25	21	48
Apurimac	2015	WHO	No	4.11	2.59	6.45	26	634
Apurimac	2015	WHO	Yes	39.43	23.26	58.3	13	32
Arequipa	2015	WHO	No	6.69	4.56	9.72	41	737
Arequipa	2015	WHO	Yes	46.13	28.05	65.29	12	28
Ayacucho	2015	WHO	No	4.07	2.75	6	30	836
Ayacucho	2015	WHO	Yes	32.97	19.25	50.38	17	47
Cajamarca	2015	WHO	No	5.4	3.74	7.73	26	667
Cajamarca	2015	WHO	Yes	27.27	14.56	45.19	11	39

Callao	2015	WHO	No	6.91	4.99	9.49	41	724
Callao	2015	WHO	Yes	51.93	38.47	65.11	30	60
Cusco	2015	WHO	No	4.58	3.07	6.78	26	676
Cusco	2015	WHO	Yes	21.2	8.55	43.66	7	34
Huancavelica	2015	WHO	No	4.83	3.33	6.94	31	669
Huancavelica	2015	WHO	Yes	25.95	13.03	45.05	11	33
Huanuco	2015	WHO	No	3.5	2.21	5.5	26	805
Huanuco	2015	WHO	Yes	27.85	14.03	47.73	9	40
Ica	2015	WHO	No	9.05	6.81	11.94	57	747
Ica	2015	WHO	Yes	58.64	41.65	73.8	30	56
Junin	2015	WHO	No	4.13	2.73	6.19	29	700
Junin	2015	WHO	Yes	26.85	11.92	49.89	7	24
La Libertad	2015	WHO	No	5.24	3.49	7.78	33	704
La Libertad	2015	WHO	Yes	50.43	32.1	68.64	19	45
Lambayeque	2015	WHO	No	6.73	4.65	9.64	48	738
Lambayeque	2015	WHO	Yes	51.27	37.03	65.31	30	53
Lima	2015	WHO	No	7.07	5.65	8.82	122	2235
Lima	2015	WHO	Yes	49.11	39.62	58.67	78	163
Loreto	2015	WHO	No	9.35	7.25	11.99	66	712
Loreto	2015	WHO	Yes	47.67	31.07	64.8	16	35
Madre de Dios	2015	WHO	No	6.17	3.88	9.67	35	645
Madre de Dios	2015	WHO	Yes	42.41	20.05	68.37	10	22
Moquegua	2015	WHO	No	4.85	3.4	6.86	30	700
Moquegua	2015	WHO	Yes	43.16	28.83	58.74	19	45
Pasco	2015	WHO	No	1.62	0.87	3	11	663
Pasco	2015	WHO	Yes	29.71	14.9	50.51	10	36
Piura	2015	WHO	No	6.92	4.81	9.87	39	710
Piura	2015	WHO	Yes	59.59	43	74.25	37	65
Puno	2015	WHO	No	2.05	1.09	3.82	15	684

Puno	2015	WHO	Yes	20.41	7.51	44.73	6	33
San Martin	2015	WHO	No	6.66	4.77	9.22	48	735
San Martin	2015	WHO	Yes	60.59	43.35	75.54	30	51
Tacna	2015	WHO	No	5.16	3.45	7.64	33	696
Tacna	2015	WHO	Yes	33.77	20.67	49.93	11	35
Tumbes	2015	WHO	No	8.95	6.5	12.19	54	749
Tumbes	2015	WHO	Yes	48.04	30.34	66.24	19	39
Ucayali	2015	WHO	No	6.42	4.41	9.25	40	722
Ucayali	2015	WHO	Yes	47.34	25.46	70.3	12	25
Amazonas	2016	WHO	No	3.64	2.03	6.42	22	733
Amazonas	2016	WHO	Yes	47.34	34.02	61.05	36	69
Ancash	2016	WHO	No	4.68	3.1	7.02	30	677
Ancash	2016	WHO	Yes	35.45	22.86	50.43	15	40
Apurimac	2016	WHO	No	5.75	4.03	8.12	36	654
Apurimac	2016	WHO	Yes	23.28	10.79	43.23	8	32
Arequipa	2016	WHO	No	5.45	3.66	8.04	31	694
Arequipa	2016	WHO	Yes	49.05	31.45	66.89	16	32
Ayacucho	2016	WHO	No	4.32	2.82	6.55	28	852
Ayacucho	2016	WHO	Yes	31.64	19.99	46.17	18	57
Cajamarca	2016	WHO	No	5.63	3.92	8.02	32	619
Cajamarca	2016	WHO	Yes	43.73	30.35	58.08	22	51
Callao	2016	WHO	No	8.07	5.87	11	48	689
Callao	2016	WHO	Yes	42.22	31.11	54.17	25	60
Cusco	2016	WHO	No	3.28	2.08	5.15	21	700
Cusco	2016	WHO	Yes	46.03	23.96	69.77	14	28
Huancavelica	2016	WHO	No	4.23	2.66	6.66	25	657
Huancavelica	2016	WHO	Yes	32.61	17.5	52.49	12	36
Huanuco	2016	WHO	No	4.35	2.88	6.52	31	828
Huanuco	2016	WHO	Yes	37.76	22.2	56.33	11	34

Ica	2016	WHO	No	6.53	4.16	10.1	34	687
Ica	2016	WHO	Yes	46.23	35.74	57.07	36	76
Junin	2016	WHO	No	3.34	1.86	5.93	17	716
Junin	2016	WHO	Yes	41.64	24.18	61.49	13	36
La Libertad	2016	WHO	No	5.56	3.65	8.39	29	655
La Libertad	2016	WHO	Yes	45.34	26.73	65.35	16	39
Lambayeque	2016	WHO	No	5.96	4.38	8.06	47	769
Lambayeque	2016	WHO	Yes	53.97	40.49	66.9	38	65
Lima	2016	WHO	No	4.75	3.72	6.05	85	2082
Lima	2016	WHO	Yes	48.26	39.67	56.96	93	188
Loreto	2016	WHO	No	7.56	5.58	10.15	49	701
Loreto	2016	WHO	Yes	31.61	18.64	48.26	19	46
Madre de Dios	2016	WHO	No	6.84	4.28	10.76	36	652
Madre de Dios	2016	WHO	Yes	55.96	26.1	82.05	10	20
Moquegua	2016	WHO	No	8.43	6.06	11.61	44	714
Moquegua	2016	WHO	Yes	39.08	21.79	59.63	16	34
Pasco	2016	WHO	No	3.72	2.26	6.08	23	664
Pasco	2016	WHO	Yes	14.79	6.28	31.02	8	40
Piura	2016	WHO	No	8.13	5.98	10.96	49	714
Piura	2016	WHO	Yes	42.2	30.52	54.82	29	66
Puno	2016	WHO	No	2.42	1.37	4.23	13	691
Puno	2016	WHO	Yes	32.56	13.51	59.87	7	28
San Martin	2016	WHO	No	6.03	4.25	8.49	42	723
San Martin	2016	WHO	Yes	58.02	41.02	73.3	24	45
Tacna	2016	WHO	No	5.81	4.02	8.34	39	756
Tacna	2016	WHO	Yes	31.52	17.29	50.35	12	34
Tumbes	2016	WHO	No	8.72	6.15	12.22	52	727
Tumbes	2016	WHO	Yes	38.65	23.69	56.12	18	42
Ucayali	2016	WHO	No	5.53	3.96	7.66	41	710

Ucayali	2016	WHO	Yes	46.02	24.33	69.34	10	19
Amazonas	2017	WHO	No	5.42	3.91	7.48	43	751
Amazonas	2017	WHO	Yes	50.94	37.71	64.04	28	59
Ancash	2017	WHO	No	5.1	3.48	7.41	29	727
Ancash	2017	WHO	Yes	37.67	22.74	55.38	18	56
Apurimac	2017	WHO	No	2.95	1.74	4.97	17	711
Apurimac	2017	WHO	Yes	25.22	12.8	43.66	11	44
Arequipa	2017	WHO	No	5.65	4.04	7.85	35	730
Arequipa	2017	WHO	Yes	50.25	33.35	67.1	19	41
Ayacucho	2017	WHO	No	4	2.85	5.58	36	833
Ayacucho	2017	WHO	Yes	38.32	25.89	52.48	22	60
Cajamarca	2017	WHO	No	4.2	2.85	6.13	28	684
Cajamarca	2017	WHO	Yes	46.45	33.08	60.34	29	68
Callao	2017	WHO	No	6.21	4.63	8.3	40	686
Callao	2017	WHO	Yes	53.27	40.04	66.05	33	60
Cusco	2017	WHO	No	4.4	2.68	7.14	22	659
Cusco	2017	WHO	Yes	37.24	20.56	57.63	15	42
Huancavelica	2017	WHO	No	3.31	2.14	5.08	24	746
Huancavelica	2017	WHO	Yes	34.1	18.56	54.02	16	49
Huanuco	2017	WHO	No	3.21	2.01	5.09	23	810
Huanuco	2017	WHO	Yes	14.81	5.89	32.58	5	30
Ica	2017	WHO	No	6.5	4.83	8.68	48	778
Ica	2017	WHO	Yes	37.52	26.34	50.21	27	74
Junin	2017	WHO	No	3.3	2.13	5.09	24	711
Junin	2017	WHO	Yes	28.55	13.77	49.99	10	35
La Libertad	2017	WHO	No	4.79	3.26	7	32	697
La Libertad	2017	WHO	Yes	37.56	22.88	54.94	17	47
Lambayeque	2017	WHO	No	6.94	4.99	9.59	45	756
Lambayeque	2017	WHO	Yes	55.23	40.18	69.38	26	48

Lima	2017	WHO	No	5.34	4.22	6.74	90	2102
Lima	2017	WHO	Yes	32.61	24.8	41.51	59	177
Loreto	2017	WHO	No	6.73	4.99	9.03	44	693
Loreto	2017	WHO	Yes	49.2	34.33	64.21	23	53
Madre de Dios	2017	WHO	No	5.73	3.88	8.4	40	680
Madre de Dios	2017	WHO	Yes	47.27	28.3	67.07	11	28
Moquegua	2017	WHO	No	5.32	3.55	7.91	29	751
Moquegua	2017	WHO	Yes	34.89	19.23	54.67	17	47
Pasco	2017	WHO	No	2.78	1.59	4.83	17	668
Pasco	2017	WHO	Yes	39.47	19.54	63.65	9	25
Piura	2017	WHO	No	4.5	2.91	6.87	28	725
Piura	2017	WHO	Yes	44.56	28.16	62.24	23	56
Puno	2017	WHO	No	3.54	2.27	5.5	26	710
Puno	2017	WHO	Yes	18.26	7.74	37.29	8	49
San Martin	2017	WHO	No	5.33	3.78	7.46	36	703
San Martin	2017	WHO	Yes	40.2	27.76	54.04	23	57
Tacna	2017	WHO	No	5.21	3.58	7.53	28	713
Tacna	2017	WHO	Yes	47.73	33.26	62.59	28	61
Tumbes	2017	WHO	No	6.81	4.89	9.42	46	739
Tumbes	2017	WHO	Yes	45.01	28.65	62.52	19	44
Ucayali	2017	WHO	No	5.74	4.04	8.09	37	679
Ucayali	2017	WHO	Yes	47.44	29.61	65.95	17	35
Amazonas	2018	WHO	No	5.6	3.85	8.06	37	839
Amazonas	2018	WHO	Yes	44.05	31.87	57	24	59
Ancash	2018	WHO	No	5.89	4.03	8.53	35	776
Ancash	2018	WHO	Yes	56.14	40.63	70.54	26	49
Apurimac	2018	WHO	No	4.92	3.34	7.19	34	773
Apurimac	2018	WHO	Yes	32.59	17.66	52.15	13	44
Arequipa	2018	WHO	No	5.16	3.75	7.05	36	733

Arequipa	2018	WHO	Yes	35.98	24.38	49.49	20	58
Ayacucho	2018	WHO	No	3.17	2.17	4.62	26	876
Ayacucho	2018	WHO	Yes	30.9	19.48	45.27	18	53
Cajamarca	2018	WHO	No	3.7	2.46	5.54	26	795
Cajamarca	2018	WHO	Yes	53.72	38.45	68.32	37	67
Callao	2018	WHO	No	7.01	4.93	9.88	43	701
Callao	2018	WHO	Yes	49.3	37.19	61.5	34	68
Cusco	2018	WHO	No	4.58	3.03	6.88	30	732
Cusco	2018	WHO	Yes	24.98	10.48	48.63	10	37
Huancavelica	2018	WHO	No	4.02	2.83	5.7	31	808
Huancavelica	2018	WHO	Yes	26.6	13.55	45.58	11	48
Huanuco	2018	WHO	No	4.11	2.89	5.81	34	840
Huanuco	2018	WHO	Yes	34.94	19.62	54.15	12	37
Ica	2018	WHO	No	7.41	5.71	9.55	50	763
Ica	2018	WHO	Yes	37.08	24.68	51.46	26	68
Junin	2018	WHO	No	3.5	2.19	5.54	21	722
Junin	2018	WHO	Yes	19.02	6.96	42.45	5	28
La Libertad	2018	WHO	No	5.76	4.28	7.7	35	724
La Libertad	2018	WHO	Yes	51.93	38.98	64.63	29	57
Lambayeque	2018	WHO	No	5.94	4.33	8.08	42	806
Lambayeque	2018	WHO	Yes	57.45	43.22	70.54	37	62
Lima	2018	WHO	No	5	4.02	6.21	112	2511
Lima	2018	WHO	Yes	41.98	34.83	49.47	95	226
Loreto	2018	WHO	No	8.87	6.5	11.99	60	696
Loreto	2018	WHO	Yes	61.32	45.62	74.98	35	58
Madre de Dios	2018	WHO	No	4.4	2.84	6.74	29	659
Madre de Dios	2018	WHO	Yes	40.74	24.1	59.81	13	39
Moquegua	2018	WHO	No	6	4.29	8.32	37	778
Moquegua	2018	WHO	Yes	46.86	30.2	64.25	21	48

Pasco	2018	WHO	No	3.6	2.22	5.8	21	689
Pasco	2018	WHO	Yes	32.77	18.33	51.43	11	36
Piura	2018	WHO	No	6.96	5.03	9.57	41	775
Piura	2018	WHO	Yes	37.13	24.96	51.19	22	57
Puno	2018	WHO	No	4.52	3.05	6.66	31	781
Puno	2018	WHO	Yes	40.88	23.86	60.41	16	47
San Martin	2018	WHO	No	5.26	3.66	7.52	34	728
San Martin	2018	WHO	Yes	42.03	28.31	57.1	22	55
Tacna	2018	WHO	No	4.68	2.54	8.47	28	807
Tacna	2018	WHO	Yes	47.8	32.11	63.93	20	45
Tumbes	2018	WHO	No	9.35	7.03	12.32	59	749
Tumbes	2018	WHO	Yes	41.52	27.29	57.31	22	50
Ucayali	2018	WHO	No	5.94	4.27	8.19	38	732
Ucayali	2018	WHO	Yes	60.29	44.74	74	27	48
Amazonas	2019	WHO	No	5.23	3.81	7.15	39	774
Amazonas	2019	WHO	Yes	50.03	36.06	63.99	32	63
Ancash	2019	WHO	No	6.3	4.52	8.71	44	761
Ancash	2019	WHO	Yes	26.22	16.11	39.66	16	54
Apurimac	2019	WHO	No	3.86	2.43	6.08	30	752
Apurimac	2019	WHO	Yes	33.81	18.05	54.22	12	45
Arequipa	2019	WHO	No	4.96	3.39	7.2	32	703
Arequipa	2019	WHO	Yes	54.01	40.2	67.23	27	56
Ayacucho	2019	WHO	No	4.19	2.86	6.1	34	893
Ayacucho	2019	WHO	Yes	43.66	29.8	58.58	22	55
Cajamarca	2019	WHO	No	3.83	2.45	5.95	24	779
Cajamarca	2019	WHO	Yes	43.72	30.55	57.84	24	56
Callao	2019	WHO	No	8.19	6.23	10.71	51	668
Callao	2019	WHO	Yes	55.61	44.33	66.33	38	70
Cusco	2019	WHO	No	5.37	3.91	7.34	41	744

Cusco	2019	WHO	Yes	33.21	18.01	52.96	11	36
Huancavelica	2019	WHO	No	4.5	3	6.68	38	791
Huancavelica	2019	WHO	Yes	23.04	13.07	37.36	19	59
Huanuco	2019	WHO	No	5.16	3.47	7.59	33	824
Huanuco	2019	WHO	Yes	44.2	25.49	64.72	14	46
Ica	2019	WHO	No	5.79	4.23	7.87	35	707
Ica	2019	WHO	Yes	49.58	36.77	62.45	35	65
Junin	2019	WHO	No	3.77	2.46	5.73	24	733
Junin	2019	WHO	Yes	26.03	14.38	42.46	10	39
La Libertad	2019	WHO	No	5.36	3.91	7.31	35	678
La Libertad	2019	WHO	Yes	52.52	37.52	67.08	30	55
Lambayeque	2019	WHO	No	6.07	4.37	8.38	38	712
Lambayeque	2019	WHO	Yes	53.69	40.59	66.3	27	54
Lima	2019	WHO	No	7.22	5.93	8.76	122	2327
Lima	2019	WHO	Yes	48.25	40.09	56.5	97	206
Loreto	2019	WHO	No	10.65	8.46	13.33	64	683
Loreto	2019	WHO	Yes	47.34	31.03	64.24	23	46
Madre de Dios	2019	WHO	No	6.93	4.94	9.64	44	663
Madre de Dios	2019	WHO	Yes	38.75	21.77	58.99	13	34
Moquegua	2019	WHO	No	6.12	4.38	8.5	33	789
Moquegua	2019	WHO	Yes	42.69	25.62	61.7	15	35
Pasco	2019	WHO	No	3.87	2.39	6.21	20	716
Pasco	2019	WHO	Yes	21.8	8.17	46.65	6	29
Piura	2019	WHO	No	4.93	3.53	6.84	33	700
Piura	2019	WHO	Yes	42.15	30.48	54.77	29	65
Puno	2019	WHO	No	3.03	1.87	4.85	23	784
Puno	2019	WHO	Yes	16.64	6.23	37.47	9	45
San Martin	2019	WHO	No	7.48	5.5	10.08	48	727
San Martin	2019	WHO	Yes	59.56	45.19	72.47	38	59

Tacna	2019	WHO	No	4.15	2.85	6.02	31	758
Tacna	2019	WHO	Yes	38.21	24.78	53.73	22	62
Tumbes	2019	WHO	No	9.3	7.09	12.09	56	689
Tumbes	2019	WHO	Yes	53.13	36.8	68.82	28	55
Ucayali	2019	WHO	No	6.05	4.37	8.32	42	728
Ucayali	2019	WHO	Yes	65.82	43.24	82.96	17	26
Amazonas	2020	WHO	No	3.9	2.44	6.19	19	507
Amazonas	2020	WHO	Yes	48.37	30.39	66.79	16	39
Ancash	2020	WHO	No	4.16	2.29	7.46	17	479
Ancash	2020	WHO	Yes	50.16	25.78	74.46	14	41
Apurimac	2020	WHO	No	3.78	2.16	6.55	19	498
Apurimac	2020	WHO	Yes	45.69	22.83	70.52	9	27
Arequipa	2020	WHO	No	7.1	4.79	10.41	31	464
Arequipa	2020	WHO	Yes	35	17.98	56.94	10	31
Ayacucho	2020	WHO	No	4.31	2.73	6.72	20	562
Ayacucho	2020	WHO	Yes	35.92	18.23	58.49	11	34
Cajamarca	2020	WHO	No	3.15	2.05	4.81	16	493
Cajamarca	2020	WHO	Yes	27.11	16.68	40.87	9	41
Callao	2020	WHO	No	8.13	6.06	10.83	35	476
Callao	2020	WHO	Yes	37.78	26.13	51.03	25	66
Cusco	2020	WHO	No	3.88	2.28	6.54	19	489
Cusco	2020	WHO	Yes	36.34	11.44	71.6	7	17
Huancavelica	2020	WHO	No	4.53	2.97	6.85	24	530
Huancavelica	2020	WHO	Yes	37.26	21.78	55.89	15	43
Huanuco	2020	WHO	No	3.88	2.2	6.73	14	545
Huanuco	2020	WHO	Yes	31.92	16.91	51.93	11	37
Ica	2020	WHO	No	6.56	4.35	9.78	25	450
Ica	2020	WHO	Yes	31.14	19.55	45.69	14	46
Junin	2020	WHO	No	4.35	2.13	8.68	18	512

Junin	2020	WHO	Yes	40.42	22.88	60.8	10	25
La Libertad	2020	WHO	No	5.49	3.57	8.36	22	494
La Libertad	2020	WHO	Yes	58.56	36.52	77.63	16	28
Lambayeque	2020	WHO	No	8.55	6.18	11.73	36	494
Lambayeque	2020	WHO	Yes	49.18	32.87	65.67	17	38
Lima	2020	WHO	No	6.68	5.42	8.2	98	1705
Lima	2020	WHO	Yes	37.8	30.12	46.14	60	179
Loreto	2020	WHO	No	9.23	6.74	12.51	49	491
Loreto	2020	WHO	Yes	32.64	18.75	50.43	13	40
Madre de Dios	2020	WHO	No	6.03	3.82	9.41	27	466
Madre de Dios	2020	WHO	Yes	32.34	14.89	56.62	7	18
Moquegua	2020	WHO	No	3.01	1.61	5.55	15	509
Moquegua	2020	WHO	Yes	50.06	30.99	69.11	16	33
Pasco	2020	WHO	No	2.12	0.89	4.97	9	458
Pasco	2020	WHO	Yes	19.49	6.18	47.07	5	28
Piura	2020	WHO	No	8.99	6.42	12.45	39	526
Piura	2020	WHO	Yes	47.18	27.99	67.24	13	30
Puno	2020	WHO	No	6.76	4.79	9.45	34	567
Puno	2020	WHO	Yes	39.68	23.34	58.7	9	31
San Martin	2020	WHO	No	7.93	5.64	11.05	35	520
San Martin	2020	WHO	Yes	47.33	34.4	60.63	18	42
Tacna	2020	WHO	No	5.8	4.04	8.27	28	484
Tacna	2020	WHO	Yes	35.65	19.88	55.3	12	41
Tumbes	2020	WHO	No	7.64	5.41	10.67	37	496
Tumbes	2020	WHO	Yes	43.58	31.32	56.67	27	58
Ucayali	2020	WHO	No	4.82	3.21	7.2	23	523
Ucayali	2020	WHO	Yes	45.85	20.47	73.59	8	18

The numerator and denominator will not exactly match the proportion (%) because the proportion was computed accounting for the complex survey design, whereas the numerator denominator are observed data.

Supplementary Figure 1. Percentage of people receiving antihypertensive medication among not eligible subjects by sex and age at the national level

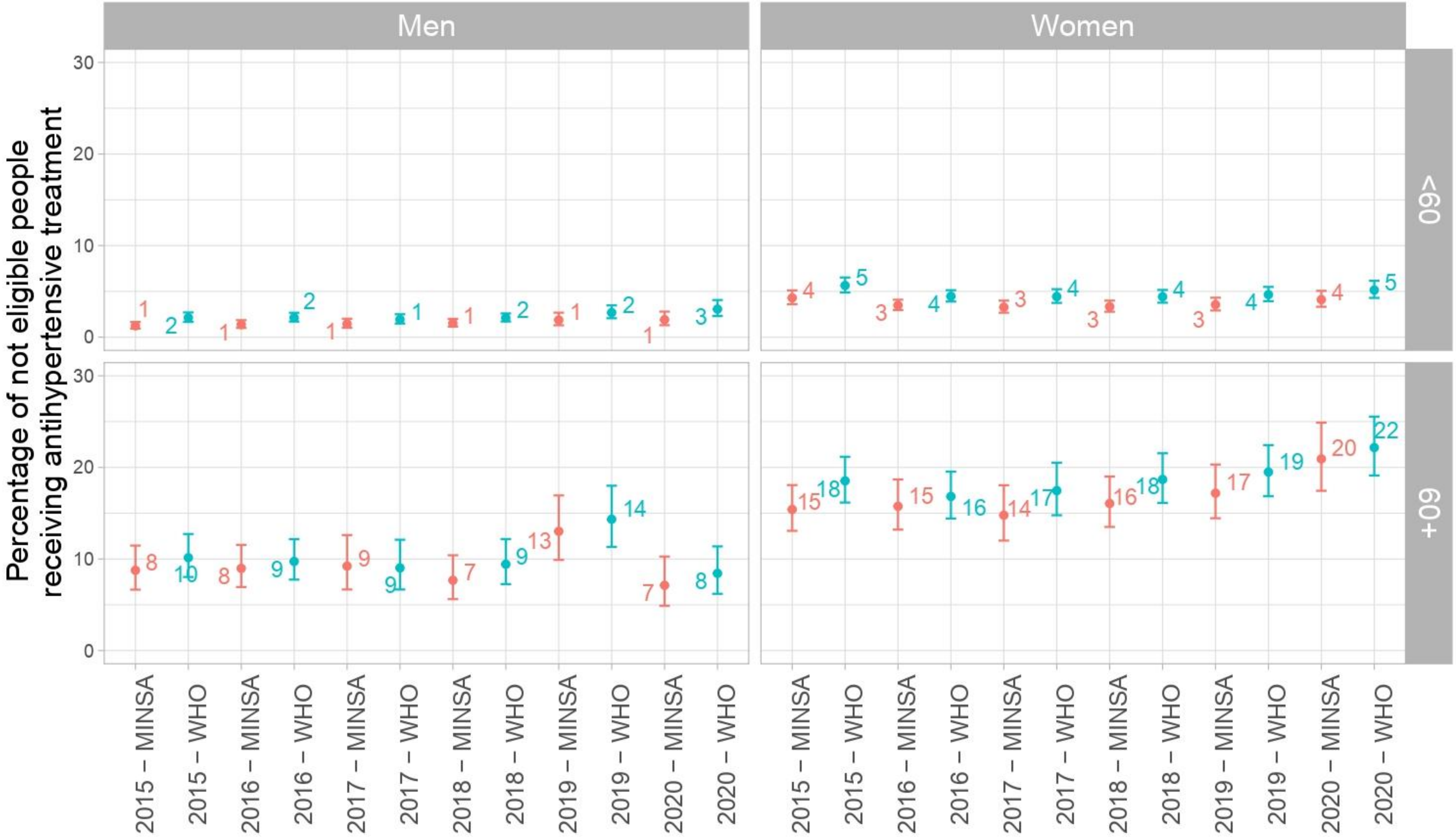

Supplementary Figure 2. Percentage of people receiving antihypertensive medication among eligible subjects by sex and macro-region

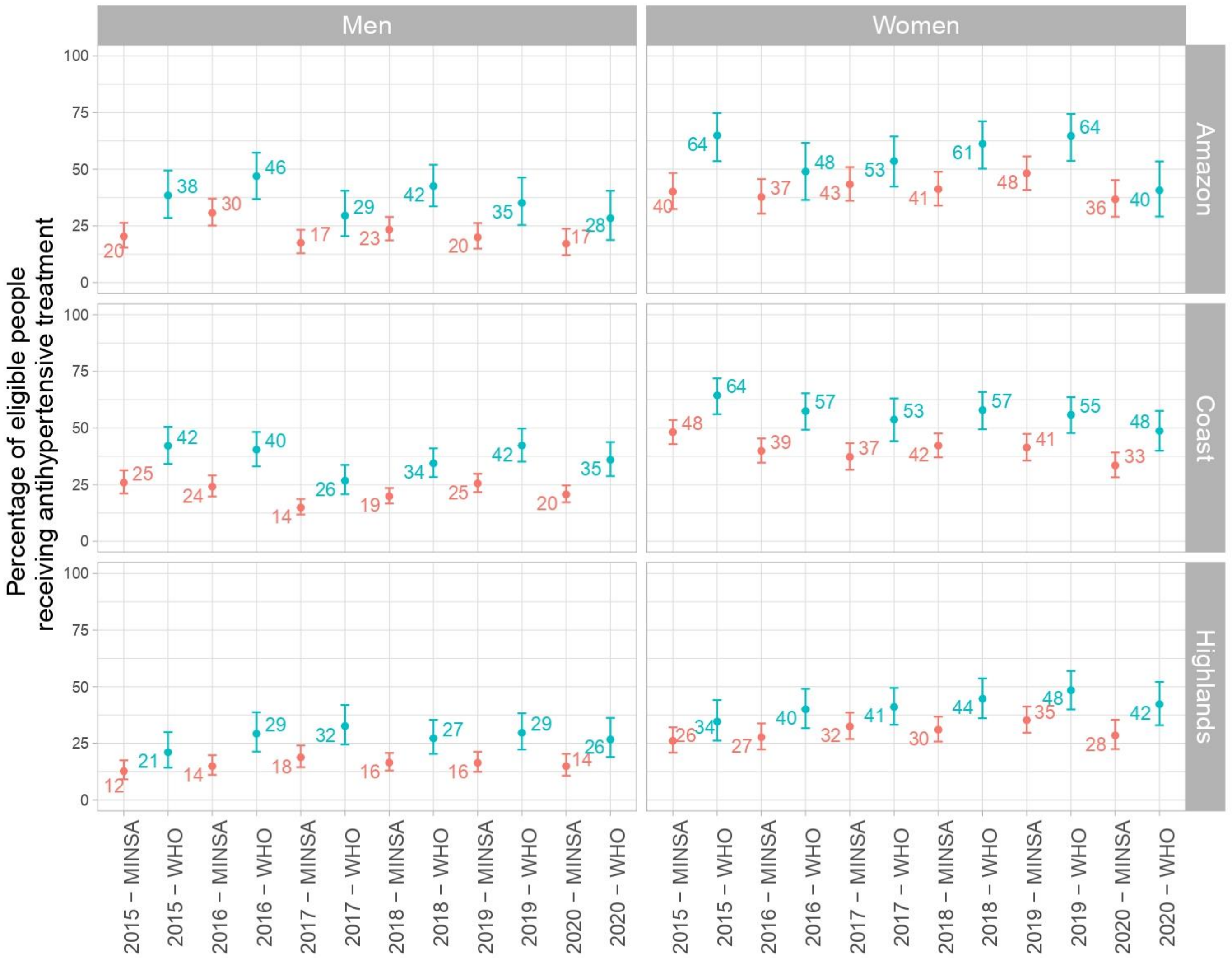

Supplementary Figure 3. Percentage of people receiving antihypertensive medication among eligible subjects by sex and macro-region

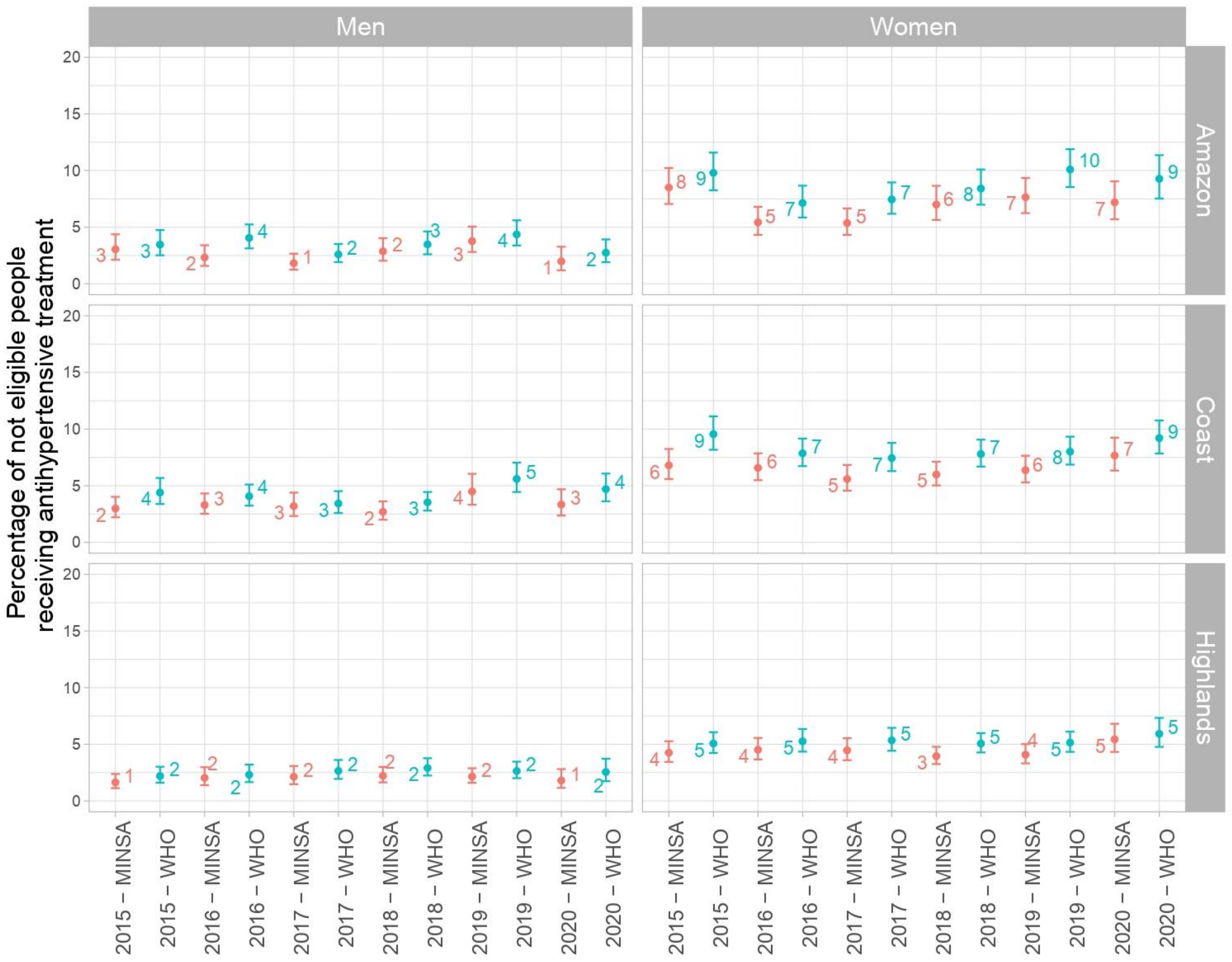

Supplementary Figure 4. Percentage of people receiving antihypertensive medication among eligible subjects at the sub-national level

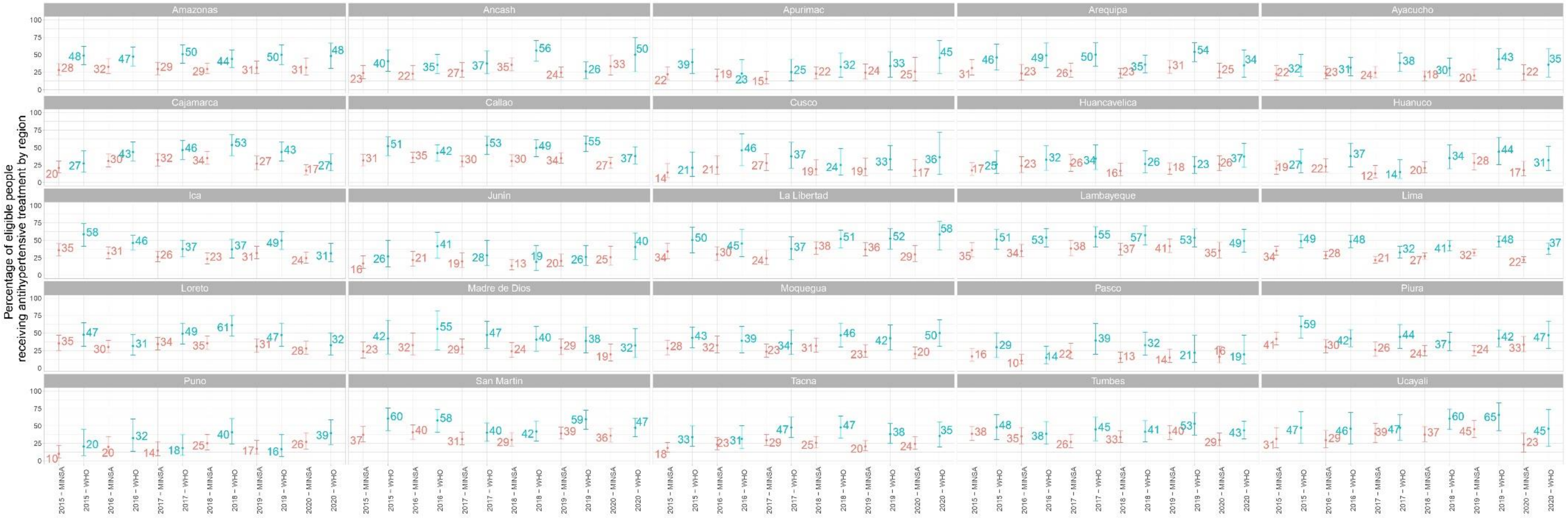

Supplementary Figure 5. Percentage of people receiving antihypertensive medication among not eligible subjects at the sub-national level

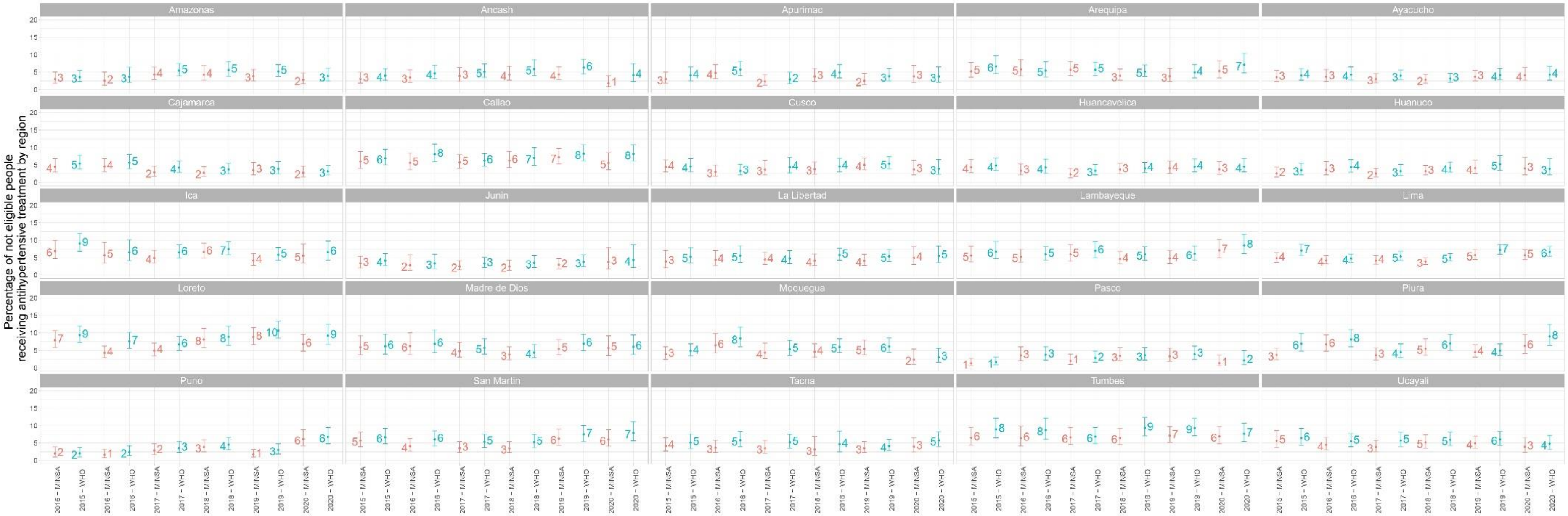

STROBE Statement—Checklist of items that should be included in reports of *cross-sectional studies*

	Item No	Recommendation
**Title and abstract**	1	(*a*) Indicate the study's design with a commonly used term in the title or the abstract (p. 01)
		(*b*) Provide in the abstract an informative and balanced summary of what was done and what was found (p. 03)
**Introduction**		
Background/rationale	2	Explain the scientific background and rationale for the investigation being reported (p. 04)
Objectives	3	State specific objectives, including any prespecified hypotheses (p. 04)
**Methods**		
Study design	4	Present key elements of study design early in the paper (p. 04)
Setting	5	Describe the setting, locations, and relevant dates, including periods of recruitment, exposure, follow-up, and data collection
Participants	6	(*a*) Give the eligibility criteria, and the sources and methods of selection of participants (p. 05)
Variables	7	Clearly define all outcomes, exposures, predictors, potential confounders, and effect modifiers. Give diagnostic criteria, if applicable (pp. 05-07)
Data sources/ measurement	8*	For each variable of interest, give sources of data and details of methods of assessment (measurement). Describe comparability of assessment methods if there is more than one group (p. 04)
Bias	9	Describe any efforts to address potential sources of bias
Study size	10	Explain how the study size was arrived at
Quantitative variables	11	Explain how quantitative variables were handled in the analyses. If applicable, describe which groupings were chosen and why
Statistical methods	12	(*a*) Describe all statistical methods, including those used to control for confounding (p. 07)
		(*b*) Describe any methods used to examine subgroups and interactions
		(*c*) Explain how missing data were addressed
		(*d*) If applicable, describe analytical methods taking account of sampling strategy (p. 04)
		(*e*) Describe any sensitivity analyses
**Results**		
Participants	13*	(a) Report numbers of individuals at each stage of study—eg numbers potentially eligible, examined for eligibility, confirmed eligible, included in the study, completing follow-up, and analysed (p. 09)
		(b) Give reasons for non-participation at each stage
		(c) Consider use of a flow diagram
Descriptive data	14*	(a) Give characteristics of study participants (eg demographic, clinical, social) and information on exposures and potential confounders (p. 09)
		(b) Indicate number of participants with missing data for each variable of interest
Outcome data	15*	Report numbers of outcome events or summary measures (p. 09-10)
Main results	16	(*a*) Give unadjusted estimates and, if applicable, confounder-adjusted estimates and their precision (eg, 95% confidence interval). Make clear which confounders were adjusted for and why they were included (p. 10)

		(*b*) Report category boundaries when continuous variables were categorized (p. 10)
		(*c*) If relevant, consider translating estimates of relative risk into absolute risk for a meaningful time period
Other analyses	17	Report other analyses done—eg analyses of subgroups and interactions, and sensitivity analyses
**Discussion**		
Key results	18	Summarise key results with reference to study objectives (p. 10)
Limitations	19	Discuss limitations of the study, taking into account sources of potential bias or imprecision. Discuss both direction and magnitude of any potential bias (pp. 14-15)
Interpretation	20	Give a cautious overall interpretation of results considering objectives, limitations, multiplicity of analyses, results from similar studies, and other relevant evidence (p. 10)
Generalisability	21	Discuss the generalisability (external validity) of the study results
**Other information**		
Funding	22	Give the source of funding and the role of the funders for the present study and, if applicable, for the original study on which the present article is based (p. 03)

*Give information separately for exposed and unexposed groups.
